# Dissecting the Polygenic Basis of Primary Hypertension: Identification of Key Pathway-Specific Components

**DOI:** 10.3389/fcvm.2022.814502

**Published:** 2022-02-16

**Authors:** Carlo Maj, Erika Salvi, Lorena Citterio, Oleg Borisov, Marco Simonini, Valeria Glorioso, Cristina Barlassina, Nicola Glorioso, Lutgarde Thijs, Tatiana Kuznetsova, Francesco P. Cappuccio, Zhen-Yu Zhang, Jan A. Staessen, Daniele Cusi, Chiara Lanzani, Paolo Manunta

**Affiliations:** ^1^Institute for Genomic Statistics and Bioinformatics, Medical Faculty, University of Bonn, Bonn, Germany; ^2^Neuroalgology Unit, Fondazione IRCCS Istituto Neurologico “Carlo Besta”, Milan, Italy; ^3^Genomics of Renal Diseases and Hypertension Unit, Istituto di Ricovero e Cura a Carattere Scientifico IRCCS San Raffaele Scientific Institute, Vita-Salute San Raffaele University, Milan, Italy; ^4^Department of Statistics and Quantitative Methods, University of Milano-Bicocca, Milan, Italy; ^5^Department of Health Sciences, University of Milan, Milan, Italy; ^6^Department of Clinical and Experimental Medicine, Hypertension and Related Diseases Centre, University of Sassari, Sassari, Italy; ^7^Research Unit Hypertension and Cardiovascular Epidemiology, KU Leuven Department of Cardiovascular Sciences, University of Leuven, Leuven, Belgium; ^8^Warwick Medical School, and UHCW NHS Trust, University of Warwick, Coventry, United Kingdom; ^9^Research Institute Alliance for the Promotion of Preventive Medicine (APPREMED), Mechelen, Belgium; ^10^Biomedical Science Group, Faculty of Medicine, University of Leuven, Leuven, Belgium; ^11^Institute of Biomedical Technologies Milano National Research Council of Italy (CNR), Milano, Italy; ^12^Bio4Dreams Scientific Unit, Bio4Dreams-Business Nursery for Life Sciences, Milano, Italy

**Keywords:** hypertension, genome-wide association studies, polygenic risk score, prediction, pathway analysis

## Abstract

**Introduction and Objectives:**

Genome-wide association studies have identified a high number of genetic loci associated with hypertension suggesting the presence of an underlying polygenic architecture. In this study, we aimed to dissect the polygenic component of primary hypertension searching also for pathway-specific components.

**Methods:**

The polygenic risk score (PRS) models, based on the UK biobank genetic signals for hypertension status, were obtained on a target Italian case/control cohort including 561 cases and 731 hyper-normal controls from HYPERGENES, and were then applied to an independent validation cohort composed by multi-countries European-based samples including 1,284 cases and 960 hyper-normal controls.

**Results:**

The resulting genome-wide PRS was capable of stratifying the individuals for hypertension risk by comparing between individuals in the last PRS decile and the median decile: we observed an odds ratio (OR) of 3.62, CI = [2.01, 6.32] (*P* = 9.01E-07) and 3.22, 95% CI = [2.06, 5.10] (*P* = 6.47E-08) in the target and validation cohorts, respectively. The relatively high case/control ORs across PRS quantiles corroborates the presence of strong polygenic components which could be driven by an enrichment of risk alleles within the cases but also by potential enrichment of protective alleles in the old normotensive controls. Moreover, novel pathway-specific PRS revealed an enrichment of the polygenic signal attributable to specific biological pathways. Among those the most significantly associated with hypertension status was the calcium signaling pathway together with other mainly related such as the phosphatidylinositol/inositol phosphate pathways.

**Conclusions:**

The development of pathway-specific PRS could prioritize biological mechanisms, according to their contribution to the genetic susceptibility, whose regulations might be a potential pharmacological preventive target.

## Introduction

Essential hypertension is the major cardiovascular risk factor and its prevalence is constantly increasing worldwide in middle or old age (1). Currently hypertension is responsible for an estimated 7.8 million deaths and 143 million disability life years lost worldwide in 2015 (2). Multiple genetic drivers interacting with environmental and lifestyle facilitators underlie the heterogeneous pathophysiology of this complex trait (3, 4) and contribute to the unpredictable responses to antihypertensive drug treatment (5, 6).

In a complex multifactorial disorder, individual gene variants with small effect *per se* are generally not informative for assessing disease risk. Advances in understanding the genetics of blood pressure (BP) confirmed that individual genetic loci have modest effects on BP (<1.0 mmHg) and no clinical meaning in the population (6, 7). However, recent genome-wide association studies (GWAS) consortia increased power to identify different new loci for BP by expanding the numbers of studies included in meta-analyses, leading to a higher number of genetic determinants associated with various BP-related traits (8, 9). A genetic loading conferred by the combined set of risk variants was successfully used to obtain a measure to discriminate high risk subjects (10). The information across loci can be combined through the polygenic risk score (PRS), a weighted sum of the number of risk alleles carried by each individual (11, 12). The combination of all BP elevating alleles could increase SBP by approximately 10 mmHg between the top and bottom quintiles of the PRS distribution, substantially increasing risk of cardiovascular events with ORs of about 1.5 (8) in two large cohorts of European ancestry. Recently, PRS associated with incident hypertension in a population based-cohort with the highest 2.5% PRS had a 2.3-fold risk of hypertension and 10.6 years earlier hypertension onset compared to individuals with an average PRS (13) and with a higher incidence of adverse cardiovascular events (14). PRS are usually derived from several variants across the genome without investigation on the underlying biological mechanism (15). However, the computation of pathway-specific PRS investigates to which extent the genetic variability located in genes belonging to specific biological processes is driving the genome-wide PRS associations (16, 17). While a classic genome-wide PRS combining the effects of all hypertension-associated SNPs may be more powerful for corroborating the presence of a highly polygenic genetic signal for hypertension, a pathway-specific PRS may be a more powerful predictor of specific biomarkers contributing to an enriched component of the underlying hypertension pathology, thus leading to a greater comprehension of individual hypertension predisposition.

In the present work, we computed both a genome-wide and a Kyoto Encyclopedia of Genes and Genomes (KEGG) derived pathway-specific PRS for hypertension in a Caucasian case/control target cohort. The derived models were then applied to an independent validation cohort in association with hypertension status. Furthermore, the analysis of hypertension pathway-specific PRS in the target and in the validation HYPERGENES cohorts revealed some pathways characterized by a prominent genetic association.

## Methods

### Study Design and Participants

Data originate from the multicenter international study “HYPERGENES–European Network for Genetic-Epidemiological Studies: building a method to dissect complex genetic traits, using essential hypertension as a disease model,” funded by the European Union 7th Framework Programme (grant HEALTH-2007-201550) (18). For the present study, we selected primary hypertensive cases having diastolic BP (DBP) ≥ 90 mmHg or systolic BP (SBP) ≥ 140 mmHg or under antihypertensive treatment before the age of 50. On the contrary, normotensives had never been treated for hypertension, presenting DBP < 90 mmHg and SBP <140 mmHg with more than 50 years of age. As target sample, 1,292 samples from Italy cohorts were selected, divided into 561 hypertensives (43.4%) and 731 normotensives (56.6%) (Table 1). As validation cohort, an independent multi-countries European-based dataset was used including 1,284 cases (57.2%) and 960 controls (42.8%) (Table 1). For both datasets, as expected by the inclusion criteria, the mean age was significantly higher in normotensives than in hypertensives. Since aging is associated with an increased prevalence of hypertension, we selected exclusively hyper-controls older than 50-year-old allowing for the exclusion of subjects that developed hypertension at a later age. For this reason, the distribution of age was bimodal and we excluded it as an explanatory variable in the PRS. Moreover, the DBP and SBP pressures were also significantly higher in hypertensives with respect to controls. All participants were unrelated and of European ancestry.

**Table 1:** Demographic and phenotypic characteristics of target and validation cohorts.

	**Target (*****N*** **= 1,292)**			**Validation (*****N*** **= 2,244)**		
**Variable**	**Hypertensives (*N* = 561, 43.4%)**	**Normotensives (*N* =731, 56.6%)**	***P*-value**	**Hypertensives (*N* = 1,284, 57.2%)**	**Normotensives (*N* = 960, 42.8%)**	***P*-value**
Male	432 (77.0%)	426 (58.3%)	<0.001	801 (62.4%)	575 (59.5%)	0.499
Age (Years)	45.5 (±7.97)	59.5 (±6.1)	<0.001	49.6 (±9.8)	62.6 (±9.96)	<0.001
BMI (kg/m^2^)	26.4 (±3.02)	25.1 (±3.3)	<0.001	27.6 (±4.3)	26.1 (±3.8)	<0.001
Diastolic BP (mmHg)	96.5 (±5.9)	79.5 (±4.6)	<0.001	98.8 (±9.7)	76.1 (±6.8)	<0.001
Systolic BP (mmHg)	148.6 (±8.93)	124.9 (±6.8)	<0.001	154.8 (±14.9)	122.5 (±10.04)	<0.001

*Continuous variables are presented as mean and standard deviation, categorical variables as numbers and percentages. Between-groups comparisons of continuous variables were performed using t-test. Categorical data were compared between groups using the chi-squared test*.

### Genotyping Data

Samples were genotyped using Illumina 1M-Duo array. As the data were already used for a GWAS on hypertension we refer the readers to the previous work for details on genotyping data generation (18). In the present work data were re-imputed using V3 of 1,000 genomes project (19). Details on genotype data processing can be found in the Supplementary Material.

### Polygenic Risk Score

PRS was computed using a clumping-thresholding approach by means of PRSice software (20). The pipeline generates clumps around SNPs (by considering flanking genomic regions of 250 kb) with *P*-value of associations lower than a given threshold and retain only variants that are not in linkage with the indexed variants (variants with an r2 of correlations > 0.1 were considered in linkage). The process is repeated iteratively through all index SNPs, starting from the smallest *P*-value and allowing each SNP to appear only in one clump. A PRS so defined contains the most significantly phenotype-associated SNPs (according to the reference GWAS) for each linkage-disequilibrium (LD) filtered clump across the entire genome. The PRS is then computed as the weighted sum of the risk alleles, where the weights represent the strength of association with the phenotype as represented by the beta coefficients of the reference genome-wide summary statistics (i.e., reference base associations for the PRS).

Polygenic risk score for hypertension were built according to the variant effect sizes retrieved from the GWAS on hypertension performed on UK Biobank dataset including 144,793 cases and 313,761 controls (21). The summary statistics from UK Biobank GWAS were used as base associations (i.e., to match variant names and corresponding genomic coordinates and to derive the betas from the GWAS to be used as weights of the tested alleles). Different PRS were created over a range of five *P*-values thresholds, from genome wide to full model, namely (1.0, 0.5, 0.05, 1E-4, 1E-6, 5E-8).

**Figure 1:**
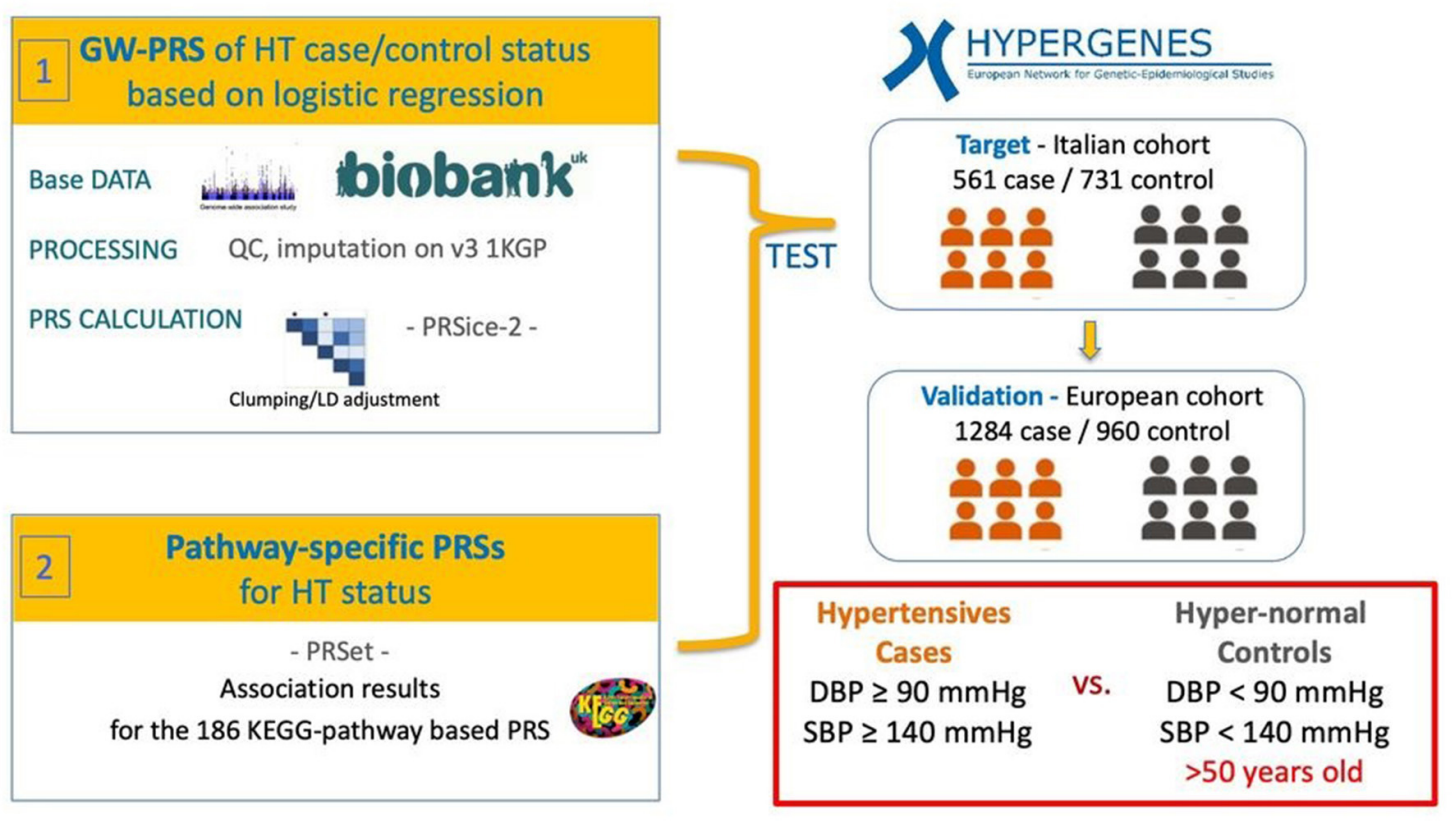
Workflow of the analysis. Hypertension-PRS based on a reference hypertension-GWAS performed on the UK Biobank were calculated in the Italian hypertension (HT) case/control target cohort from the HYPERGENES dataset. The models were then validated in an independent European-wide HT case/control cohort within the HYPERGENES dataset. The analysis was performed both for genome-wide PRS and for pathway-specific PRS according to 186 KEGG-pathways.

The PRS models are based on logistic regression analysis of hypertension case/control status considering PRS as explanatory variable and including sex, BMI and the first 4 principal components as covariates to adjust for potential residual population stratification. In order to assess the relative importance of PRS the partial Nagelkerke's pseudo-R2 is computed as the difference between the R2 of the full model including covariates and PRS and the only covariates model. The best fitting model was selected according to the higher partial R2. The flowchart for the study methodology is available as Figure 1.

In order to test for the prediction performance of the models the receiving operating curves (ROC) were derived and the corresponding area under the curves (AUC) were computed. The DeLong's test was applied to compare the AUC of the full models and of the covariate-models and thus to assess the additional specific contribution in the prediction performance attributable to the PRS.

### Pathway-Specific PRS

Beside genome-wide PRS also pathway-specific PRS were generated by associating variants to genes according to chromosome coordinates as implemented in PRSet (20), http://www.prsice.info/prset_detail/). Both exonic and intronic variants were mapped to genes and considered in the pathway-specific PRS (intergenic variants were excluded). The genes associated with essential hypertension were analyzed for functional significance in the various metabolic pathways using the KEGG (Kyoto Encyclopedia of Genes and Genomes-http://www.genome.jp/kegg/) (22). A total of 186 KEGG pathways were considered in the analysis using the Molecular Signatures Database v7.1 (https://www.gsea-msigdb.org/gsea/msigdb/).

Since pathway-specific PRS are based on a small number of variants, full models (i.e., no *P*-value filtering) after clumping were generated for each pathway so to capture the overall genetic signal present in the pathway, an approach proved to be effective to obtain to study pathways-specific associations (23). False discovery rate was applied to correct for multiple-testing. Beside self-contained *P*-values also pathway-specific competitive *P*-values were generated through a permutation based-approach aimed at the evaluation of a potential enrichment in the genetic signal attributable to specific gene-sets. Namely a background set of SNPs is defined by considering all variants belonging to any genes and thus included for pathway-specific PRS. The pathway specific *P*-value is compared to the null distribution of *P*-values obtained by randomly selecting n times (where n is the number of permutations) k variants from the background set (where k is number of variants in the analyzed pathway specific PRS).

In the present work 10 k permutations were applied to derive the empirical *P*-values (i.e., competitive *P*-values) used to assess to which extent the genetic signal is enriched in a given pathway with respect to the underlying genome-wide association signal.

The z-score for each pathway was derived as the ratio between the PRS effect size and standard error of the corresponding logistic regression while the Pearson correlation coefficient of the z-scores was used to assess the correlation of the pathway-specific PRS in the target and in the validation cohort.

## Results

### Polygenic Risk Score

**Figure 2:**
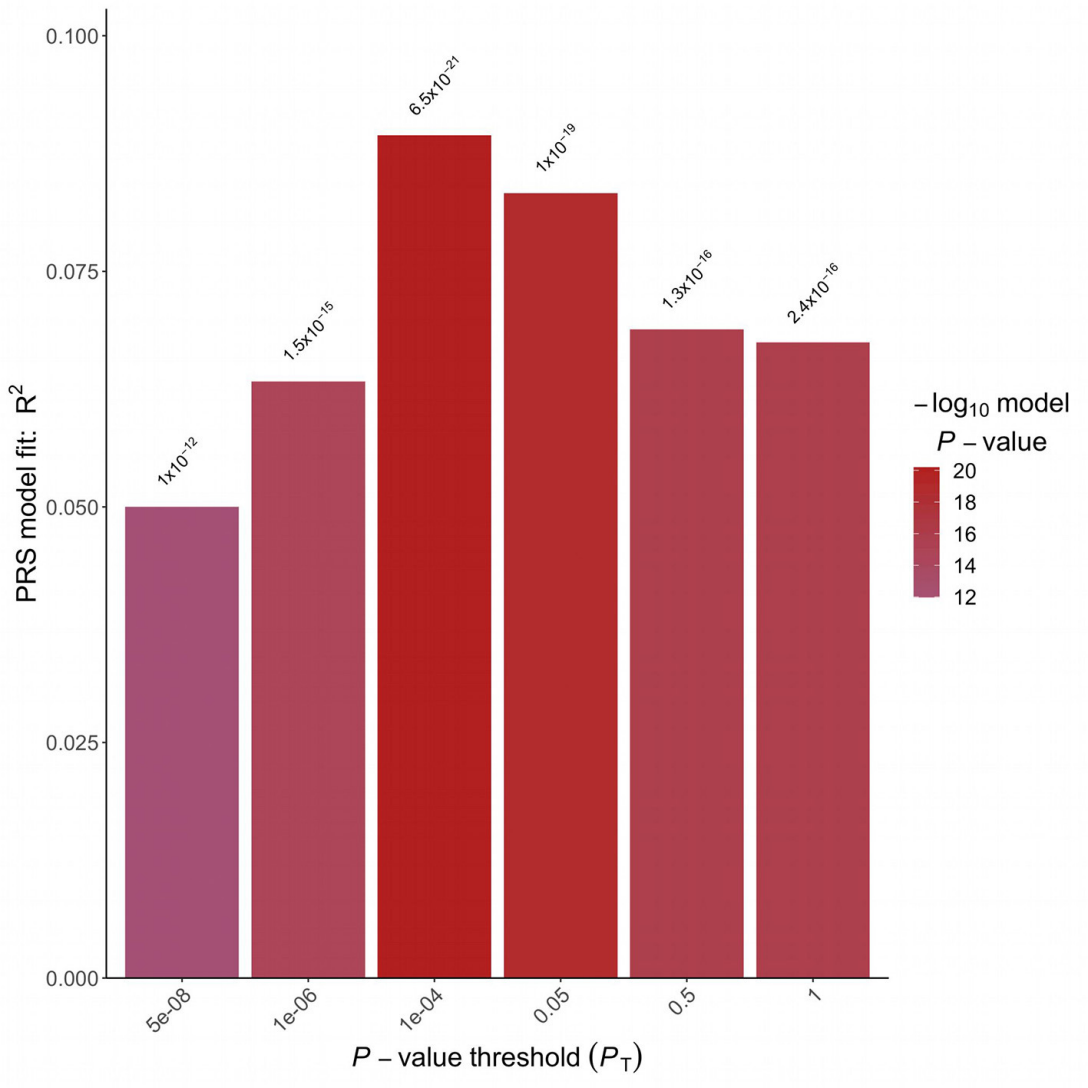
Polygenic risk score analysis. Bar plot displaying the model fit of the PRS at *P-value* threshold. In the x-axis are reported the *P*-value thresholds used to filter for the variants to be included in the PRS computation. Above the bar are reported the *P*-values of the logistic association test.

A logistic regression analysis including PRS and adjusted for sex, BMI and 4 Principal Components was implemented. A strong association between the PRS and hypertension status in the target Italian cohort was detected considering all the analyzed *P*-value thresholds (Figure 2). The best fitting model was based on 2,166 clumped variants (Supplementary Table 1) and reached a *P*-value of associations of 6.46 E-21 and a partial Nagelkerke R2 of 9.46%. The model was then applied to the validation cohort and the association between PRS and hypertension status was replicated (*P*-value = 2.92E-41, R2 = 11.64%). The distribution of the PRS in the two cohorts is shown in Supplementary Figure 1.

**Figure 3:**
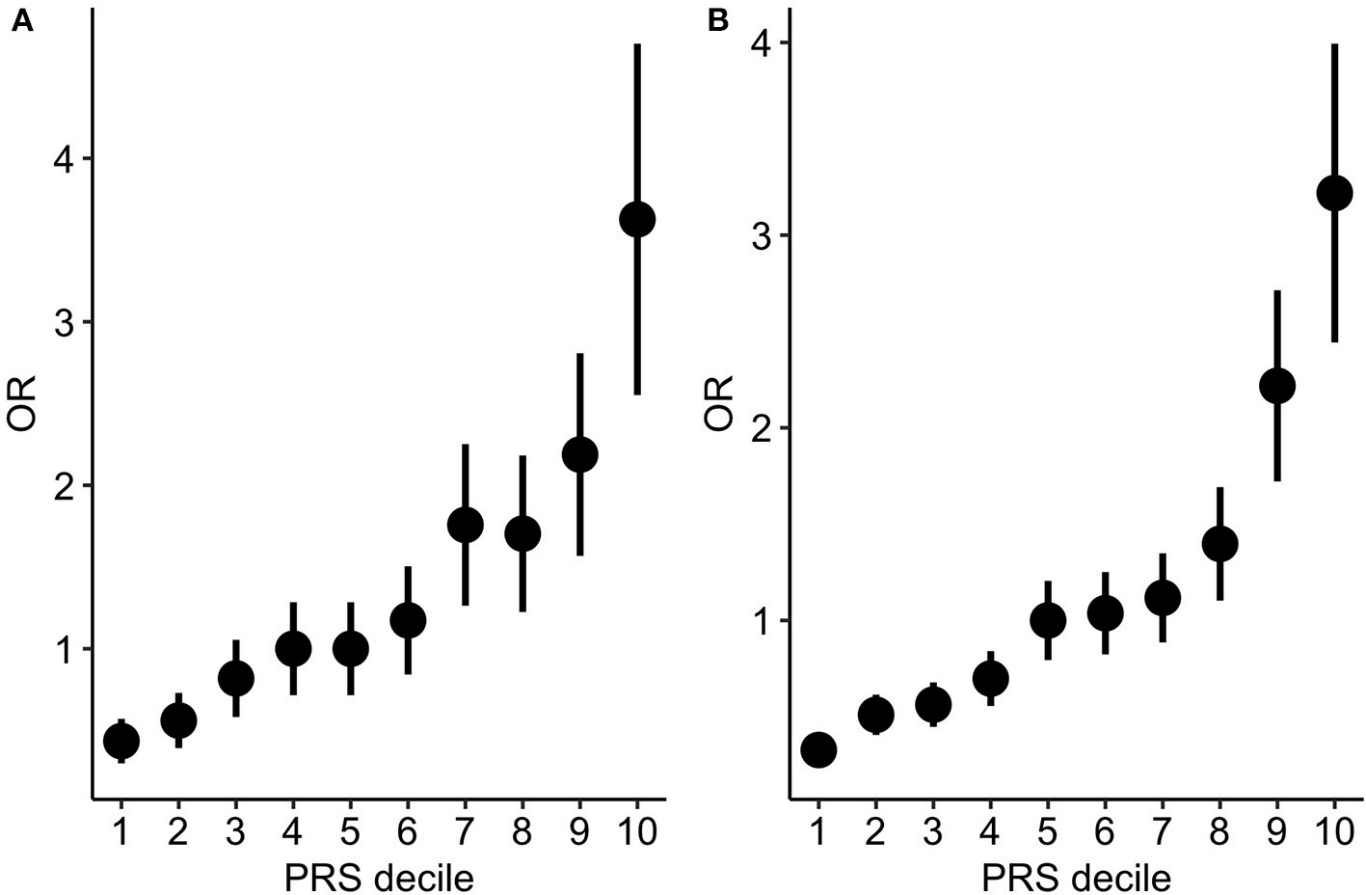
PRS stratification for hypertension risk. ORs between hypertensive cases and normotensive controls by grouping the samples according to the PRS deciles (**A**: target cohort; **B**: validation cohort). The points represent the OR point estimates and the lines represent the corresponding standard errors.

In order to test to which extent PRS can differentiate individuals for the hypertension risk we stratified the individuals in deciles according to the PRS and we compared the OR across the different groups. Both in the target and in the validation cohort we observed that the higher the decile the higher the OR (Table 2, Figure 3).

**Table 2:** OR for hypertension across PRS deciles.

**PRS decile**	***OR* target**	***N* controls target**	***N* cases target**	***P* target**	***OR* validation**	***N* controls validation**	***N* cases validation**	***P* validation**
1	0.44	102	28	2.85E-03	0.32	155	70	1.03E-08
2	0.56	96	34	3.4E-02	0.51	132	93	4.73E-04
3	0.82	85	44	0.52	0.56	126	98	3.35E-03
4	1	79	50	1	0.69	114	110	0.071
5	1	79	50	1	1	94	130	1
6	1.17	74	55	0.61	1.03	92	132	0.92
7	1.76	61	68	3.34E-03	1.11	88	136	0.63
8	1.70	62	67	4.51E-02	1.39	76	147	0.096
9	2.18	54	75	2.72E-03	2.21	55	169	1.31E-04
10	3.62	39	90	9.13E-07	3.21	41	183	6.47E-08

*Number of controls and cases across PRS decile and corresponding OR and P-values in the target and in the validation cohort*.

**Figure 4:**
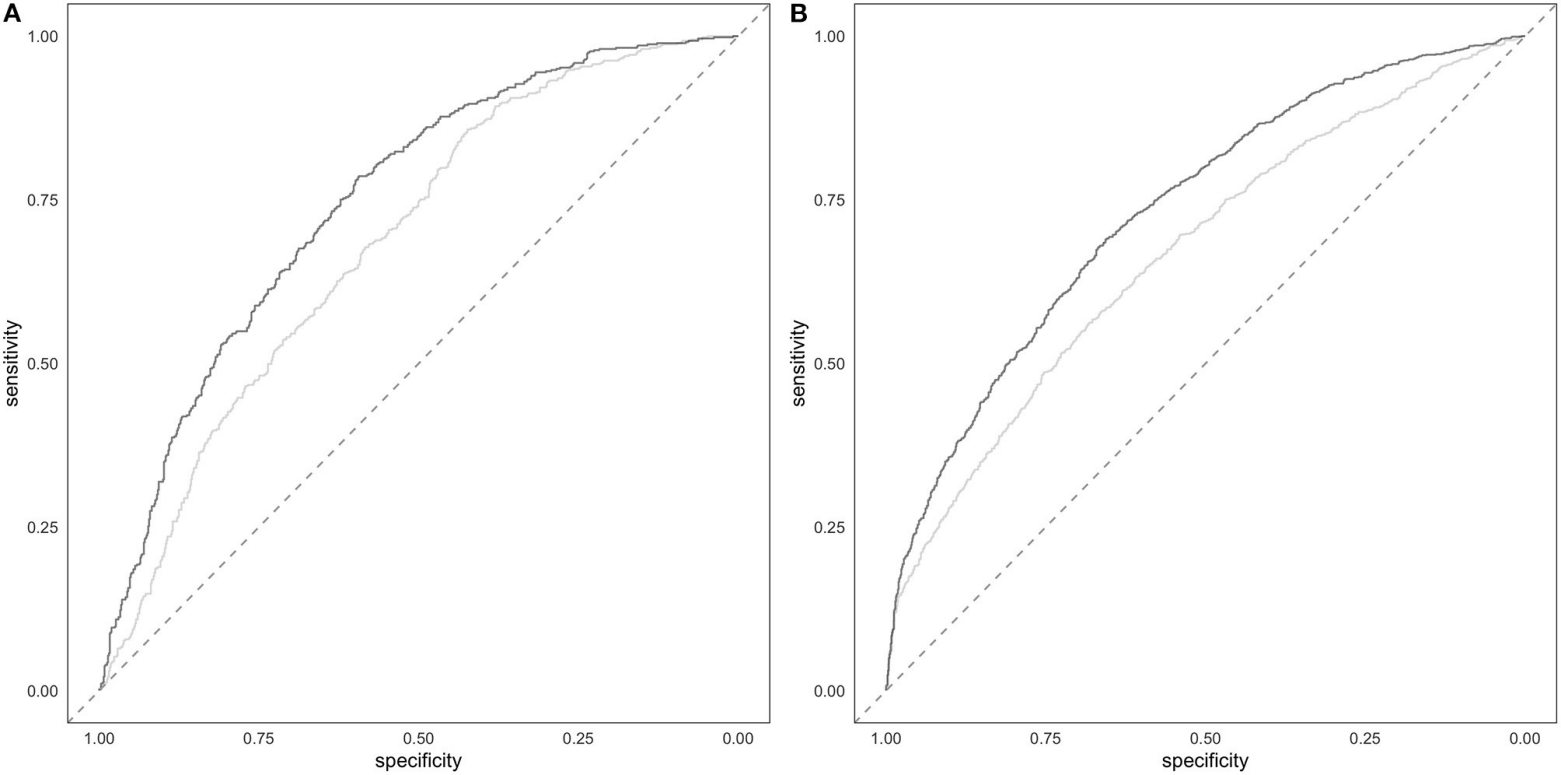
Prediction of hypertension. Target **(A)** and validation **(B)** ROC of the full model including PRS and covariates (darker line) and of the covariates-only (i.e., BMI, sex and 4PC) model (lighter line).

The comparison of the ROC in full model and in the covariate-only model in both cohorts showed that PRS improves the discrimination between cases and controls (Figure 4). The AUC of the full model including PRS was significantly greater than the AUC reached by the only covariates model (DeLong's test *P* = 2.47E-09 and *P* = 8.97E-12 in the target and in the validation cohort, respectively). Specifically, the AUC of the covariates-only model (thus including BMI, sex and 4PC) reached and AUC of 0.68 and 0.66 in the target and validation cohort, respectively. The inclusion of PRS in the model led to an AUC of 0.75 and 0.73 in the target and in the validation cohort, respectively.

### Pathway-Specific PRS

We further examined the pathway-specific PRS for enrichment of the polygenic signal by analyzing the polygenic signal that can be attributed to KEGG pathways by considering variants located in gene-region. A total of 175,488 variants across 11,950 genes were included in pathway analysis. Pathway-specific PRS in the target cohort detected a total of 21 significant (FDR adjusted) associations out of the 186 analyzed KEGG pathways derived-PRS (see Supplementary Table 2). The identified 21 pathway-based PRS were also significantly enriched with respect to the background PRS signal as assessed by the empirical-competitive (Emp_P) *P*-value. Pathway-specific PRS were also computed in the multi-countries validation cohort by considering the same variants.

A moderate correlation has been observed in the Z-scores of pathways-specific PRS in the target cohort (Supplementary Table 2) and in the validation cohort with the Person correlation coefficient equal to 0.34, 95% CI [0.21, 0.34], *P*-value = 1.67E-07). Of the 21 significant pathways identified in the target cohort, 7 were replicated (see Table 3, Supplementary Table 2). Among those the most significantly associated with hypertension status was the calcium signaling pathway (*P*_FDR_ target = 0.0010, *P*_FDR_ validation = 0.0012). We also found evidence of enrichment for pathways involved in the phosphatidylinositol signaling system and the inositol phosphate metabolism, in the mammalian target of rapamycin (mTOR) and the vascular endothelial growth factor (VEGF) signaling pathways, and in the focal adhesion and the Huntington disease pathways (Table 2, Supplementary Table 2). Gene-enrichment analysis showed a total of 675 univocal genes (Supplementary Table 3), some of which were shared among five pathways (*n* = 4), four (*n* = 14), three (*n* = 18) or between two pathways (*n* = 89). The most common genes among the pathways were related to the phosphatidylinositol-4,5-bisphosphate 3-kinase, the phosphoinositide-3-kinase regulatory subunit, the inositol-trisphosphate 3-kinase gene families as well as the phospholipase C isoforms and the Akt serine/threonine kinases.

**Table 3:** Significant pathway-specific PRS in both target and validation cohorts.

**Set**	***P*_FDR_ target**	**Zscore target**	***P*_FDR_ validation**	**Zscore validation**	***N* SNP**
Calcium signaling	0.001	4.40	0.0012	4.32	1,669
VEGF signaling	0.021	3.17	0.0035	3.96	439
Focal adhesion	0.0097	3.54	0.014	3.46	1,575
Phosphatidylinositol signaling system	0.01	3.43	0.027	3.10	758
Huntington's disease	0.037	2.91	0.027	3.12	799
MTOR signaling	0.045	2.78	0.033	2.95	278
Inositol phosphate metabolism	0.01	3.45	0.0349	2.94	439

*P_FDR_ target and P_FDR_ validation refer to P-value in the target cohort and in the validation cohort, respectively; Z score target and Z score validation refer to represent the Z-value of the PRS regression coefficients; N SNP indicates the number of SNPs included to compute the pathway-specific PRS*.

## Discussion

In the present study a strong association between PRS based on UK Biobank data and the hypertension status was detected in two independent case/control cohorts. The best fitting model was obtained considering 2,167 clumped variants confirming that hypertension is a highly polygenic trait as already verified in other larger cohorts (8, 9).

A recent work describing the association of PRS for BP with incident hypertension in >200,000 of the FinnGenn population based-cohort showed that individuals with the highest 2.5% PRS have a 2.3-fold risk of hypertension and 10.6 years earlier hypertension onset compared to individuals with an average PRS (13). Another PRS for hypertension based on 858 independent SNPs was found to be associated with a higher incidence of adverse cardiovascular events (14) even if to date, PRS are based on data and cohorts composed of largely European-ancestry individuals, and the transferability of their performance for other ethnicities is a particularly important challenge. More recently, PRS for cardiometabolic traits in sub-Saharan Africans in comparison with African Americans and European Americans showed a greater predictive utility for BP traits among Europeans and a poorer performance in Africans (24), maybe reflecting the choice of SNPs and weights for PRS differing between populations due to LD and allele frequency patterns differences.

The PRS analysis for hypertension reported in the present work regarded a target and validation case/control samples from HYPERGENES, collected over many years in different European regions. Compared to the above previous results in this framework, the peculiarity of the current samples was the inclusion of older hyper-controls in this cohort previously analyzed for a classical GWAS but not yet for a potential polygenic component (18). Despite the relative small sample size the presence of individuals with early-onset hypertension and old normotensive controls represent an optimal cohort for a PRS analysis as a prominent genetic component can be expected for a trait otherwise characterized by a predominant environmental component (25). The relatively high case/control ORs across PRS quantiles corroborates the presence of strong polygenic components which could be driven by an enrichment of risk alleles within the cases but also by potential enrichment of protective alleles in the old normotensive controls (26). Furthermore, PRS may identify individuals missed by canonical clinical risk prediction models, particularly those at high risk for early-onset disease, as already seen for most cardiovascular diseases where risk calculators have been trained with data limited on middle-aged individuals (27). In line with this hypothesis, we observed that in HYPERGENES dataset a prediction model for hypertension combining both clinical risk factors (i.e., sex and BMI) and PRS reached a significantly higher AUC with respect to a model based on only covariates.

Beside the primary purpose in the definition of disease status, PRS may be also informative to gain insight into the potential disease-related biological pathways by addressing their genetic contribution in PRS (28).

Our findings reporting the analysis of hypertension pathway-specific PRS in the target and in the validation HYPERGENES cohorts revealed some pathways characterized by a prominent genetic association. Strikingly, calcium signaling and phosphatidylinositol/inositol phosphate pathways were intuitive candidates that have been extensively implicated in hypertension, whereas others such as the mTOR, the VEGF, the focal adhesion signaling pathways, and particularly the Huntington disease pathways are unexpected. In hypertension, many of the mechanisms regulating intracellular calcium homeostasis are perturbed. Calcium intracellular concentration and signaling control the key functions of vascular smooth muscle cells (VSMCs), leading to activation of the phospholipase C (PLC)-mediated phosphoinositide hydrolysis, inositol 1,4,5-trisphosphate (IP3) and diacylglycerol (DAG) (PLC-IP3-DAG) pathway (29, 30), but also influence activity of many transcription factors and proteins, transporters, channels and exchangers thereby impacting the cellular phenotype and function. Other calcium-independent processes regulating VSMCs contraction influence the calcium sensitization, and include the DAG-PLC-PKC pathway and the RhoA-Rho kinase (ROCK) pathway (31).

The phosphatidylinositol 4,5-bisphosphate 3-kinases (PI3Ks) are common to the inositol phosphate metabolism and to the phosphatidylinositol signaling system. These enzymes mediate intracellular transduction pathways by catalyzing the phosphorylation of phosphatidylinositol 4,5-bisphosphate (PIP2) upon several stimuli (32). Furthermore, PI3K signaling has been shown to be involved in many mechanisms related to vascular function, by the activation of protein kinase B (Akt) (33). The second messenger IP3 may activate the endoplasmic reticulum calcium-release channel inositol trisphosphate receptor (IP3R) (34).

In this study, the KEGG pathway enrichment analysis showed that also VEGF pathway was associated with hypertension, generally considered to be the main pathway responsible for angiogenesis (35). The VEGF inhibition is known to exert multiple actions that are detrimental to the cardiovascular system. For example, VEGF blockade results in endothelial dysfunction, leading to a decrease in nitric oxide formation in endothelial cells, and subsequently to impaired vasorelaxation (36). Moreover, increases in BP occur almost universally with VEGF inhibitor (VEGFi) therapy, the most common cardiovascular toxicity of this therapy, occurring in up to 80% of patients (37), together with proteinuria (38).

Focal adhesions (FAs) are integrin-containing, multi-protein structures that form mechanical links between intracellular actin bundles and the extracellular matrix (ECM) in many cell types (39), and also serve as signal sensors to concentrate and direct numerous signaling proteins at sites of integrin binding and clustering (40). FA and ECM formation is affected by the primary effector hormone, angiotensin II (Ang II) by promoting the association of scaffolding proteins (41), while an increase in intracellular calcium ion concentration activating enzymes like calcineurin and calpain mediates FA disassembly (42, 43). Phosphorylation of focal adhesion kinase and its subcellular translocation to FAs require the coordination of mTOR complex 1 (mTORC1) activation and microfilament remodeling (44).

The mTOR, a conserved serine/threonine protein (42, 43) is among the validated pathways and its signaling is involved in a variety of processes including cell survival, proliferation, differentiation and migration. mTORC1 seems to integrate vascular-related signaling arising from oxidative stress, pro-inflammatory cytokines, and the renin-angiotensin system all of which can lead to vascular dysfunction (45, 46). mTORC1 signaling is also a critical regulator of cardiovascular function as a major downstream component of PI3K/Akt pathway involved in regulation of cardiac growth in the response to pressure overload (47).

In this study the most apparently hypertension-unrelated KEGG pathway is the Huntington's disease (HD), as it is a monogenic neurodegenerative disease but also influenced by genetic and environmental modifiers (48). Interestingly, a relationship of HD with hypertension and age at onset of motor symptoms was recently found on the Enroll-HD dataset by two parallel studies. Hypertension was associated with a more rapid decline in motor scores and total functional capacity (49), and with a higher annualized hazard of onset of motor HD in the presence of hypertension, by examining prospectively the relationship between the reported diagnosis of hypertension (50).

Different limitations characterizes the present study aimed at dissecting the polygenic component of hypertension. The analysis has been performed only on European individuals from the UK Biobank and HYPERGENES projects (18). However, previous observations showed that the genetic effects on BP of common SNPs genetic effects may be consistent between populations (9, 51). Nonetheless, due to the difference in LD and allele frequencies we expect that the PRS will not have the same accuracy in non-European population (52). Furthermore, as the analyzed genotyping data are based on post-imputed genotyping datasets the selected variants for the best-PRS might be a proxy in LD with the true causal variants.

In conclusion, the PRS reported here, which sum up the effect of numerous genetic variants, are capable of stratifying the individuals for hypertension status both in a target and a validation cohort. These findings provide insights into the clinically relevant performance of PRS for case–control discrimination, possibly before the clinical manifestation, as the genetic make-up of an individual is largely stable from birth; therefore, genetic information can potentially be used as an early risk predictor (53). Moreover, we computed pathway-specific PRS to investigate to which extent the genetic variability located in genes belonging to specific biological processes is driving the current genome-wide PRS associations, thus prioritizing biological mechanisms overlapping putative core biological previously studied and newly mechanisms. The advent of clinically relevant polygenic scores and the related biological specific-pathways mechanisms also purposes novel backgrounds for potential pharmacological preventive targeting in several pathologies related to hypertension. Further studies of personalized treatment of each individual could allow randomization to clinical trials and biomarker studies to be stratified based on evidence of involvement of specific biological pathways.

## Data Availability Statement

The original contributions presented in the study are included in the article/Supplementary Material, further inquiries can be directed to the corresponding author/s.

## Author Contributions

CM, ES, LC, CL, and PM conceived and designed the project. MS, VG, CB, NG, LT, TK, FC, Z-YZ, JS, DC, CL, and PM recruited the subjects and performed the assessments of patients. CB performed the genotyping. CM and OB analyzed data. CM, ES, and LC wrote the manuscript. CL and PM revised critically the manuscript content. All the authors read and approved the final manuscript.

## Funding

This work was supported by the HYPERGENES project (European Network for Genetic-Epidemiological Studies: building a method to dissect complex genetic traits, using essential hypertension as a disease model), grant HEALTH-F4-2007-201550, funded by EU within the FP7. CM receives support by the BONFOR-program of the Medical Faculty, University of Bonn (Grant Number O-147.0002.1). APPREMED (URL: http://www.appremed.org) received a non-binding grant from Omron Healthcare Co., Ltd., Kyoto, Japan.

## Conflict of Interest

The authors declare that the research was conducted in the absence of any commercial or financial relationships that could be construed as a potential conflict of interest.

## Publisher's Note

All claims expressed in this article are solely those of the authors and do not necessarily represent those of their affiliated organizations, or those of the publisher, the editors and the reviewers. Any product that may be evaluated in this article, or claim that may be made by its manufacturer, is not guaranteed or endorsed by the publisher.
