# Supplementary material for: Dissecting the Polygenic Basis of Primary Hypertension: Identification of Key Pathway-Specific Components
Source: Front Cardiovasc Med. 2022 Feb 16;9:814502. doi: 10.3389/fcvm.2022.814502 (PMC8888857; doi:10.3389/fcvm.2022.814502)
Supplement: Supplementary file 1 [file Data_Sheet_1.PDF]

Supplementary Material

1 SUPPLEMENTARY DATA

1.1 Genotype data processing

Quality control (QC) on genotyping data was applied. Markers with minor allele frequency <1% or with a call rate <0.98 were excluded from the analysis. In addition, markers were excluded if there was deviation from Hardy–Weinberg equilibrium at $p<10^{-8}$ in controls or $p<10^{-10}$ in patients. Exclusion criteria for samples were sex-mismatched between documented and genotyped sex and a call rate <0.98. Un-relatedness between individuals was evaluated by checking kinship coefficients using the program KING.[1]

Principal component analysis was performed to check for population stratification after pruning to remove potential biases due to linkage disequilibrium. Pruning and principal component analysis was performed with PLINK.[2] Pruning was performed considering the option --indep 50 5 2 within PLINK while the first 10 Principal Components (PC) were computed. All samples were from European origin as confirmed by principal component analysis with respect to the five super populations of 1000 genomes. Outliers detection was performed considering 6 standard deviations from the mean of the top 10 inferred axis in a sequential manner until no outliers are detected. No case/control stratification was observed in the PCA subspace as confirmed by t-test applied on eigenvectors (minimum P value=0.12).

The post-QC data dataset was subjected to imputation using 1000 genome Genomes Project V3 (https://www.internationalgenome.org/category/phase-3/). Shape-IT tool22 was used for genotype phasing while impute2 was used for imputation of phased haplotypes.[3] Rare and not well imputed variants were excluded for the PRS analysis. Namely variants with a post-imputation INFO score<0.4 and a Minor Allele Frequency (MAF)<1% were removed.

1 Manichaikul A, Mychaleckyj JC, Rich SS, Daly K, Sale M, Chen W-M. Robust relationship inference in genome-wide association studies. *Bioinformatics* 2010; 26:2867.

2 Purcell S, Neale B, Todd-Brown K, Thomas L, Ferreira MAR, Bender D, *et al.* PLINK: A tool set for whole-genome association and population-based linkage analyses. *Am J Hum Genet* 2007; 81:559–575.

3 Delaneau O, Marchini J, Zagury J-F. A linear complexity phasing method for thousands of genomes. *Nat Methods 2011 92* 2011; 9:179–181.

2 SUPPLEMENTARY TABLES AND FIGURES

2.1 Figures

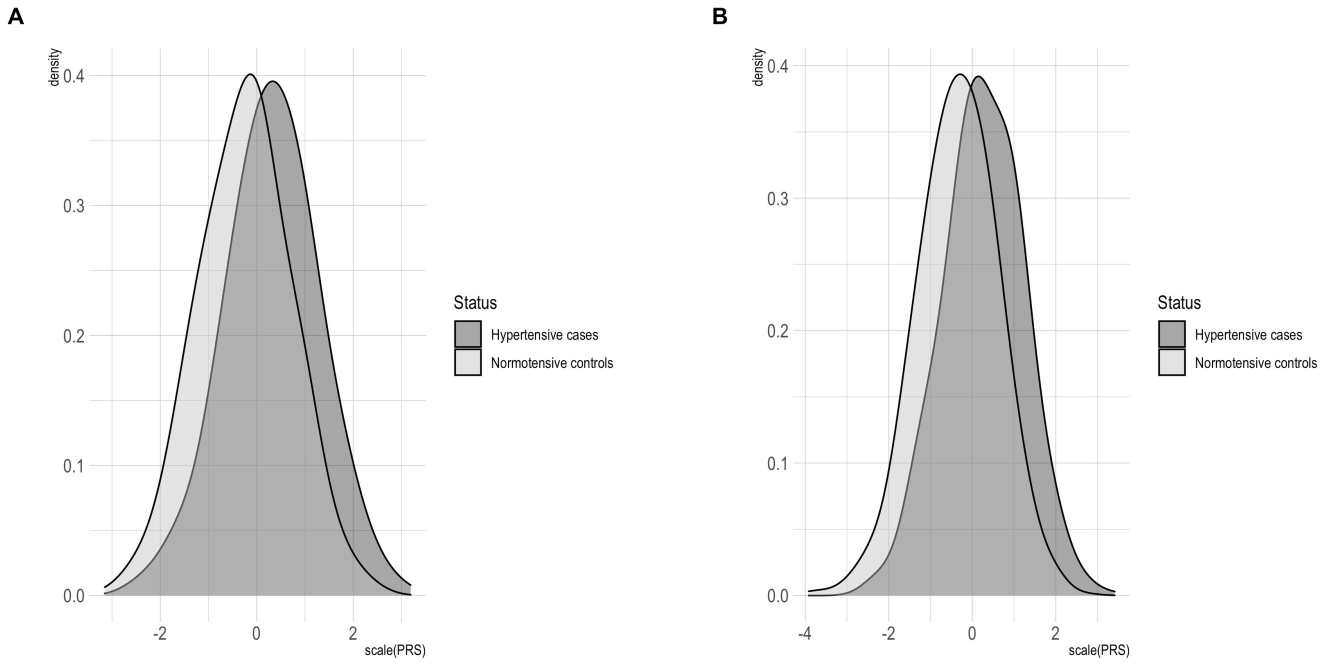

Figure S1. The distribution of the PRS in A) target and B) validation cohorts.

2.2 Tables

SNPid	CHR	pos	Effect/other alleles	Effect allele Freq	beta	SE	pval	closest gene
rs1887320	20	10965998	G/A	0.476	-0.048	0.004	2.00E-38	LOC101929413-C20orf187
rs35429	12	115555867	A/G	0.385	0.05	0.004	9.00E-38	TBX3-MED13L
rs740746	10	115792787	G/A	0.267	-0.054	0.004	2.00E-36	NHLRC2-ADRB1
rs57866767	10	96023077	T/C	0.438	0.043	0.004	6.00E-30	PLCE1
rs111663960	6	26082220	A/G	0.12	-0.063	0.006	2.00E-27	LOC108783645,H1-2-LOC108783645
rs10832571	11	16261224	C/T	0.202	-0.046	0.005	4.00E-24	SOX6
rs11105381	12	90106835	A/G	0.195	-0.048	0.005	9.00E-24	ATP2B1,ATP2B1-AS1,ATP2B1-AS1-LINC02399
rs56352102	11	10268593	C/T	0.185	-0.042	0.005	4.00E-19	SBF2
rs2240042	7	27242617	T/C	0.095	-0.057	0.006	5.00E-19	HOXA13,HOTTIP
rs11212104	11	107126353	G/A	0.44	-0.031	0.004	5.00E-16	GUCY1A2-CWF19L2
rs10061288	5	114389826	A/G	0.48	0.031	0.004	5.00E-16	LOC101927078-TRIM36
rs6077983	20	10942704	T/C	0.203	0.037	0.005	7.00E-15	LOC101929413-C20orf187
rs2301597	17	43173273	T/C	0.432	0.028	0.004	2.00E-13	NMT1
rs72804586	5	157886144	C/T	0.124	-0.041	0.006	4.00E-13	LINC02227-EBF1
rs34550591	1	11973523	A/C	0.048	0.063	0.009	6.00E-13	RNU5E-1-KIAA2013
rs4845843	1	10801585	G/A	0.14	-0.039	0.005	7.00E-13	CASZ1
rs2606735	3	11400714	A/C	0.334	-0.029	0.004	1.00E-12	ATG7
rs17282218	16	49766909	C/T	0.288	-0.029	0.004	4.00E-12	ZNF423
rs75290815	11	47197153	C/T	0.065	-0.052	0.007	6.00E-12	PACSIN3,MIR6745,ARFGAP2
rs11676969	2	113696838	G/A	0.21	0.031	0.005	6.00E-12	IL37-IL36G
rs1002226	11	17405617	C/T	0.376	0.026	0.004	8.00E-12	KCNJ11,NCR3LG1-KCNJ11
rs7221079	17	75345027	T/C	0.251	0.03	0.004	8.00E-12	SEPTIN9
rs6840119	4	38379495	G/A	0.306	0.028	0.004	1.00E-11	LINC02513
rs6453401	5	77829440	A/C	0.453	0.025	0.004	1.00E-11	LHFPL2
rs8070737	17	3981066	G/T	0.18	-0.031	0.005	4.00E-11	ZZEF1
rs10883543	10	102552752	G/T	0.106	-0.039	0.006	5.00E-11	PAX2
rs76821272	12	112009141	A/G	0.028	0.073	0.011	6.00E-11	ATXN2
rs6141328	20	31225752	G/A	0.154	-0.034	0.005	9.00E-11	C20orf203
rs1051533	14	69259662	C/A	0.215	-0.029	0.005	1.00E-10	ZFP36L1
rs1463486	17	73943942	G/A	0.222	0.03	0.005	1.00E-10	ACOX1
rs2968862	7	150650975	T/C	0.384	0.024	0.004	3.00E-10	KCNH2
rs6085527	20	6366990	A/G	0.413	-0.023	0.004	5.00E-10	FERMT1-CASC20
rs2251828	6	15479363	G/A	0.244	-0.026	0.004	6.00E-10	JARID2
rs7794284	7	74098056	A/G	0.239	-0.027	0.004	6.00E-10	GTF2I
rs68085857	1	217737629	C/T	0.229	-0.026	0.004	2.00E-09	GPATCH2
rs2728604	12	20168678	C/A	0.137	0.032	0.006	3.00E-09	LINC02398

SNPid	CHR	pos	Effect/other alleles	Effect allele Freq	beta	SE	pval	closest gene
rs7132012	12	8832203	A/G	0.323	0.024	0.004	3.00E-09	MFAP5-RIMKLB
rs2128416	1	10700448	T/C	0.134	-0.033	0.005	3.00E-09	CASZ1
rs1009358	2	65276452	T/C	0.379	0.024	0.004	3.00E-09	SLC1A4-CEP68
rs4705986	5	132349654	T/G	0.059	0.046	0.008	3.00E-09	ZCCHC10
rs7831859	8	101676642	T/C	0.432	0.022	0.004	3.00E-09	SNX31-PABPC1
rs1549306	16	75415341	T/C	0.41	-0.023	0.004	4.00E-09	CFDP1
rs7818891	8	110042172	G/A	0.423	0.021	0.004	8.00E-09	TMEM74-TRHR
rs77848533	6	150938023	C/T	0.129	0.032	0.006	9.00E-09	PLEKHG1,PLEKHG1
rs12602456	17	4273483	C/T	0.232	-0.024	0.004	2.00E-08	UBE2G1,UBE2G1-SPNS3
rs7264385	20	57789801	A/G	0.183	-0.027	0.005	2.00E-08	ZNF831
rs2497814	10	18363924	G/A	0.114	-0.033	0.006	3.00E-08	SLC39A12-CACNB2
rs1274640	14	68668528	C/T	0.144	0.03	0.005	3.00E-08	RAD51B
rs12917120	15	75329091	T/C	0.337	0.022	0.004	3.00E-08	PPCDC
rs1014988	1	1680219	G/A	0.498	0.021	0.004	3.00E-08	SLC35E2A,NADK,SLC35E2A-NADK
rs75655984	20	57704925	G/A	0.086	0.037	0.007	3.00E-08	SLMO2-ATP5E-ZNF831
rs7540957	1	15492032	A/G	0.385	0.022	0.004	4.00E-08	C1orf195,TMEM51,C1orf195
rs7694643	4	109017528	G/A	0.358	0.021	0.004	5.00E-08	LEF1
rs79349787	20	10719144	T/C	0.071	-0.039	0.007	7.00E-08	JAG1-LINC01752
rs12517393	5	113881183	T/G	0.168	-0.027	0.005	9.00E-08	LOC101927078
rs3027160	17	8057572	T/C	0.226	0.023	0.004	1.00E-07	PER1,VAMP2,PER1-VAMP2
rs12069420	1	94727624	G/A	0.23	-0.023	0.004	2.00E-07	ARHGAP29-ABCD3
rs16905190	8	135598265	T/C	0.369	-0.02	0.004	2.00E-07	ZFAT
rs7095308	10	121426312	G/A	0.271	0.022	0.004	3.00E-07	BAG3
rs10410594	19	37477758	C/T	0.157	0.027	0.005	3.00E-07	ZNF568
rs56189237	6	31328518	G/A	0.129	-0.033	0.007	3.00E-07	HLA-B,HLA-B-MICA-AS1
rs1621	7	116437606	G/A	0.325	0.021	0.004	3.00E-07	MET
rs2108790	7	7255479	C/T	0.386	-0.02	0.004	3.00E-07	C1GALT1
rs1982754	15	81081617	G/A	0.243	-0.022	0.004	4.00E-07	CEMIP
rs4788591	16	72020323	A/G	0.22	-0.023	0.005	4.00E-07	PKD1L3
rs6133612	20	8711169	G/T	0.153	0.026	0.005	4.00E-07	PLCB1
rs61325058	6	50971153	G/A	0.338	-0.02	0.004	4.00E-07	TFAP2B-PKHD1
rs3219211	12	109536559	A/C	0.181	0.025	0.005	5.00E-07	UNG
rs28925904	4	144359490	C/T	0.024	-0.059	0.012	5.00E-07	GAB1
rs6784935	3	21745877	A/C	0.35	0.02	0.004	6.00E-07	ZNF385D
rs72742734	5	32773275	A/G	0.049	0.043	0.009	6.00E-07	NPR3
rs12549801	8	142441642	A/G	0.238	-0.021	0.004	8.00E-07	PTP4A3,MROH5
rs6474859	9	14829712	T/C	0.387	0.02	0.004	8.00E-07	FREM1
rs17743054	18	42900892	T/C	0.288	0.019	0.004	9.00E-07	SLC14A2
rs9839629	3	11719413	C/A	0.314	-0.019	0.004	9.00E-07	VGLL4
rs2691159	10	18914096	C/T	0.469	-0.018	0.004	1.00E-06	NSUN6

SNPid	CHR	pos	Effect/other alleles	Effect allele Freq	beta	SE	pval	closest gene
rs1486400	12	116026433	G/A	0.433	-0.018	0.004	1.00E-06	TBX3-MED13L
rs4687477	3	185348351	T/C	0.391	-0.019	0.004	1.00E-06	SENP2
rs371708	5	87514004	G/T	0.258	0.021	0.004	1.00E-06	TMEM161B
rs7760564	6	15530908	A/G	0.215	0.022	0.005	1.00E-06	DTNBP1
rs10110464	8	110109084	C/T	0.051	-0.04	0.008	1.00E-06	TRHR
rs139562826	11	50050097	A/C	0.076	-0.034	0.007	2.00E-06	OR4C12-LOC441601
rs650198	12	69674595	C/T	0.271	0.02	0.004	2.00E-06	CPSF6-LYZ
rs112450872	17	62201863	T/C	0.065	-0.037	0.008	2.00E-06	ERN1
rs568	1	203713133	G/A	0.198	-0.022	0.005	2.00E-06	ATP2B4
rs12537548	7	26385709	T/C	0.08	-0.033	0.007	2.00E-06	SNX10
rs2074580	7	27833853	A/G	0.22	-0.021	0.005	2.00E-06	TAX1BP1
rs628480	10	106014467	G/A	0.024	0.06	0.012	3.00E-06	GSTO1,GSTO1
rs11076171	16	56912517	G/T	0.091	0.03	0.006	3.00E-06	SLC12A3
rs574626	18	55882192	C/T	0.341	-0.019	0.004	3.00E-06	NEDD4L
rs6019562	20	47584822	G/T	0.272	-0.019	0.004	3.00E-06	ARFGEF2
rs4722596	7	26491234	C/T	0.104	-0.027	0.006	3.00E-06	LOC441204
rs115555393	8	51984309	C/T	0.01	-0.085	0.019	3.00E-06	SNTG1-PXDNL
rs72924707	11	65576803	T/C	0.025	0.055	0.012	4.00E-06	OVOL1-SNX32
rs12563597	1	56760177	C/T	0.436	0.017	0.004	4.00E-06	LINC01755-LINC01767
rs12509142	4	102655378	T/G	0.465	0.017	0.004	4.00E-06	FLJ20021-BANK1
rs75915153	6	15733655	C/T	0.063	0.034	0.008	4.00E-06	DTNBP1-MYLIP
rs7748938	6	25200414	T/C	0.286	0.019	0.004	4.00E-06	CMAHP-LOC101928663
rs7941828	11	30430331	C/T	0.355	0.018	0.004	5.00E-06	MPPED2,MPPED2
rs112591226	12	90431828	C/A	0.021	0.059	0.013	5.00E-06	LINC02399-LINC02392
rs13061156	3	49747560	C/T	0.057	0.036	0.008	5.00E-06	RNF123
rs11007130	10	28786282	C/T	0.246	-0.02	0.004	6.00E-06	LINC02652,LINC02652-WAC-AS1
rs2728632	12	20146527	C/A	0.227	-0.02	0.005	6.00E-06	AEBP2-LINC02398
rs505066	1	96882671	C/A	0.277	-0.019	0.004	6.00E-06	LINC01787-PTBP2
rs12413013	10	17261762	G/A	0.302	-0.019	0.004	7.00E-06	VIM-AS1
rs619551	18	69107033	C/A	0.26	0.019	0.004	7.00E-06	GTSCR1-LINC01541
rs10794668	1	25322777	A/G	0.272	-0.019	0.004	7.00E-06	RUNX3-MIR4425
rs2736224	8	116832332	G/A	0.21	-0.021	0.005	7.00E-06	TRPS1-LINC00536
rs36013966	8	89302448	G/A	0.235	0.019	0.004	7.00E-06	MMP16
rs7047384	9	102066983	C/T	0.255	0.019	0.004	7.00E-06	SEC61B-NAMA
rs7913222	10	14101112	T/C	0.042	0.039	0.009	8.00E-06	FRMD4A
rs17519764	10	96346949	C/T	0.152	-0.023	0.005	8.00E-06	HELLS
rs12595176	15	66825689	T/C	0.156	-0.023	0.005	8.00E-06	ZWILCH
rs9848950	3	158330831	T/C	0.098	0.027	0.006	8.00E-06	MLF1-GFM1
rs6790228	3	56985697	C/A	0.421	0.017	0.004	8.00E-06	ARHGEF3,ARHGEF3-AS1
rs17241375	13	20278520	T/G	0.125	-0.024	0.006	9.00E-06	PSPC1
rs76360526	2	170481727	G/A	0.163	0.024	0.005	9.00E-06	PPIG

SNPid	CHR	pos	Effect/other alleles	Effect allele Freq	beta	SE	pval	closest gene
rs9509664	13	21932459	G/A	0.311	-0.018	0.004	1.00E-05	MIPEPP3
rs8005165	14	61629053	A/G	0.303	0.018	0.004	1.00E-05	SLC38A6-TMEM30B
rs411888	17	29963016	T/C	0.424	0.018	0.004	1.00E-05	MIR365B-COPRS
rs55901551	18	74375320	G/A	0.463	-0.017	0.004	1.00E-05	LINC01927-LINC01879
rs59053029	19	7225866	C/T	0.154	-0.023	0.005	1.00E-05	INSR
rs113156082	1	28414244	T/G	0.057	0.035	0.008	1.00E-05	EYA3
rs6108695	20	10743024	T/G	0.474	0.016	0.004	1.00E-05	LINC01752-LOC101929413
rs9613569	22	28541025	C/A	0.068	0.033	0.007	1.00E-05	TTC28
rs2119021	2	122007378	A/C	0.447	-0.017	0.004	1.00E-05	TFCP2L1
rs10865184	2	42895816	G/A	0.18	0.021	0.005	1.00E-05	MTA3
rs6755887	2	50242834	A/G	0.469	0.017	0.004	1.00E-05	NRXN1
rs4715428	6	53709740	C/T	0.493	0.016	0.004	1.00E-05	LRRC1
rs1988599	6	9551250	A/G	0.297	0.018	0.004	1.00E-05	LOC100506207-TFAP2A
rs11998686	8	51615893	T/G	0.196	-0.02	0.005	1.00E-05	SNTG1
rs4877803	9	86339006	C/T	0.35	0.017	0.004	1.00E-05	LOC105376114-GKAP1
rs17875473	10	115800294	C/T	0.078	-0.029	0.007	2.00E-05	ADRB1,NHLRC2-ADRB1
rs414299	19	49155758	T/C	0.486	0.016	0.004	2.00E-05	SEC1P
rs12058910	1	25333264	G/A	0.064	0.034	0.008	2.00E-05	RUNX3-MIR4425
rs7598027	2	38066227	T/C	0.258	-0.018	0.004	2.00E-05	LINC00211
rs9998440	4	38607801	A/C	0.208	-0.019	0.005	2.00E-05	LINC02278-KLF3-AS1
rs114111069	5	111934250	T/C	0.035	0.042	0.01	2.00E-05	EPB41L4A-DT-LINC02200
rs10519336	5	112480104	G/A	0.262	-0.019	0.004	2.00E-05	MCC
rs10491506	5	114067221	G/A	0.025	-0.051	0.012	2.00E-05	LOC101927078
rs6880509	5	132645858	A/G	0.15	0.022	0.005	2.00E-05	FSTL4
rs39298	7	21460818	C/T	0.207	0.02	0.005	2.00E-05	LINC01162-SP4
rs6999120	8	62650548	A/G	0.453	-0.016	0.004	2.00E-05	MIR4470-LINC02155
rs73667299	9	109621966	A/G	0.139	0.024	0.005	2.00E-05	ZNF462,LINC01505-ZNF462
rs17574227	9	122184572	C/T	0.14	0.023	0.005	2.00E-05	BRINP1-LINC01613
rs7864548	9	35902236	G/A	0.408	-0.016	0.004	2.00E-05	HRCT1,OR13J1-HRCT1
rs112903652	10	18368179	C/T	0.024	0.051	0.012	3.00E-05	SLC39A12-CACNB2
rs3099156	10	60614620	T/C	0.215	-0.019	0.005	3.00E-05	BICC1-LINC00844
rs1603481	11	91850764	T/C	0.49	0.016	0.004	3.00E-05	DISC1FP1-FAT3
rs10459190	12	97599429	G/A	0.477	0.016	0.004	3.00E-05	NEDD1-RMST
rs1012919	14	35884639	T/G	0.347	-0.016	0.004	3.00E-05	NFKBIA-INSM2
rs1684608	16	4676852	C/A	0.195	-0.02	0.005	3.00E-05	MGRN1
rs3746499	20	44509762	T/C	0.378	0.016	0.004	3.00E-05	ZSWIM1,ZSWIM3,ZSWIM3-ZSWIM1
rs55830103	20	57465943	T/G	0.167	-0.022	0.005	3.00E-05	LOC101927932,GNAS
rs12483320	21	47964259	A/G	0.176	0.02	0.005	3.00E-05	DIP2A
rs13003093	2	205966901	G/A	0.308	0.017	0.004	3.00E-05	PARD3B
rs1909583	3	124643551	G/T	0.258	0.018	0.004	3.00E-05	MUC13

SNPid	CHR	pos	Effect/other alleles	Effect allele Freq	beta	SE	pval	closest gene
rs11726084	4	143509808	C/A	0.075	-0.03	0.007	3.00E-05	INPP4B
rs9393292	6	22445545	C/T	0.316	-0.017	0.004	3.00E-05	PRL-HDGFL1
rs788711	7	46010714	T/C	0.273	0.017	0.004	3.00E-05	IGFBP3-LOC730338
rs75368319	8	64573196	C/T	0.044	0.037	0.009	3.00E-05	LOC102724612-LINC01289
rs7470757	9	86770499	G/T	0.041	0.04	0.009	3.00E-05	LOC101927575-SLC28A3
rs12943620	17	78444658	T/G	0.224	-0.018	0.005	4.00E-05	NPTX1
rs4890499	18	42585761	G/A	0.254	-0.017	0.004	4.00E-05	SETBP1
rs41286767	1	91485955	T/C	0.131	0.022	0.006	4.00E-05	ZNF644
rs34828267	2	228194653	C/T	0.324	-0.017	0.004	4.00E-05	MFF-DT,MFF
rs1399913	3	161375580	T/C	0.46	-0.015	0.004	4.00E-05	OTOL1-LINC01192
rs112933728	3	177016610	G/A	0.04	-0.038	0.009	4.00E-05	LINC00501
rs34324285	3	45415678	A/C	0.306	0.016	0.004	4.00E-05	TMEM158-LARS2
rs2866888	4	107622500	G/T	0.168	0.02	0.005	4.00E-05	LINC02173-DKK2
rs7723144	5	101050780	G/A	0.269	-0.018	0.004	4.00E-05	ST8SIA4-SLCO4C1
rs485434	6	147511721	G/A	0.476	-0.015	0.004	4.00E-05	STXBP5-AS1
rs9482094	6	98364895	A/G	0.368	-0.016	0.004	4.00E-05	LOC101927314-MIR2113
rs17818981	8	25894943	C/T	0.287	-0.018	0.004	4.00E-05	EBF2
rs57359666	8	39636863	C/A	0.137	-0.023	0.006	4.00E-05	ADAM2
rs8065492	17	13494023	A/G	0.157	-0.02	0.005	5.00E-05	HS3ST3A1
rs2849253	18	48317203	T/C	0.495	0.015	0.004	5.00E-05	MRO,MAPK4-MRO
rs1984694	19	15028093	A/G	0.206	-0.019	0.005	5.00E-05	OR7A17-OR7C2
rs78356357	1	89143642	C/T	0.046	0.035	0.009	5.00E-05	PKN2-AS1
rs7364197	22	23887749	C/T	0.121	0.023	0.006	5.00E-05	PCAT14
rs2710246	2	219533894	T/C	0.39	-0.015	0.004	5.00E-05	STK36,RNF25
rs4864410	4	138258752	A/C	0.184	0.019	0.005	5.00E-05	LINC02511-PCDH18
rs6897940	5	50535522	C/T	0.348	0.016	0.004	5.00E-05	LINC02106-LOC642366
rs11754129	6	126892049	C/A	0.032	-0.04	0.011	5.00E-05	MIR588-RSPO3
rs141770119	8	105879395	A/G	0.023	0.05	0.012	5.00E-05	LRP12-ZFPM2
rs1359847	9	13899978	C/T	0.23	0.018	0.004	5.00E-05	LINC01235-LINC00583
rs12580702	12	51331917	G/A	0.332	-0.016	0.004	6.00E-05	METTL7A-HIGD1C
rs850651	14	57193476	T/C	0.048	-0.035	0.009	6.00E-05	TMEM260-OTX2
rs2287842	19	47588325	G/A	0.288	-0.016	0.004	6.00E-05	ZC3H4
rs6738097	2	101578542	T/C	0.143	-0.021	0.005	6.00E-05	NPAS2
rs6728459	2	28999336	A/G	0.289	0.017	0.004	6.00E-05	PPP1CB
rs2161956	5	51717417	G/A	0.497	0.015	0.004	6.00E-05	LINC02118-PELO
rs74505362	6	120387516	T/C	0.147	0.022	0.005	6.00E-05	MIR3144-TBC1D32
rs9363976	6	69635627	C/A	0.315	-0.016	0.004	6.00E-05	ADGRB3
rs2811899	9	86231801	C/T	0.246	0.018	0.004	6.00E-05	FRMD3-IDNK
rs688413	10	84385896	A/C	0.198	0.02	0.005	7.00E-05	NRG3
rs78215522	1	163261918	C/T	0.072	0.027	0.007	7.00E-05	RGS5
rs11709103	3	27421024	G/A	0.139	0.022	0.005	7.00E-05	SLC4A7

SNPid	CHR	pos	Effect/other alleles	Effect allele Freq	beta	SE	pval	closest gene
rs13137622	4	89062513	G/T	0.304	-0.017	0.004	7.00E-05	ABCG2
rs4699413	4	96161724	G/A	0.352	-0.016	0.004	7.00E-05	UNC5C
rs4410607	5	60126266	T/C	0.185	0.019	0.005	7.00E-05	ELOVL7
rs850501	7	145308055	G/A	0.474	0.016	0.004	7.00E-05	TPK1-CNTNAP2
rs334495	7	47545795	T/C	0.262	0.017	0.004	7.00E-05	TNS3
rs10259976	7	94364483	G/A	0.297	-0.016	0.004	7.00E-05	PEG10-PPP1R9A
rs687428	9	75510376	A/G	0.221	0.018	0.005	7.00E-05	LINC01474-ALDH1A1
rs2512923	11	55807977	G/A	0.368	-0.016	0.004	8.00E-05	OR5AS1-OR8I2
rs458522	16	3330179	C/T	0.243	0.017	0.004	8.00E-05	ZNF263,LINC00921-ZNF263
rs4795637	17	30027452	T/C	0.229	-0.019	0.004	8.00E-05	MIR365B-COPRS
rs4822211	22	43267831	G/T	0.454	-0.015	0.004	8.00E-05	PACSIN2
rs115466119	3	35141990	A/C	0.043	0.034	0.009	8.00E-05	LOC101928135
rs34208976	4	102790037	G/T	0.318	0.016	0.004	8.00E-05	BANK1
rs114814118	5	118639462	T/C	0.022	0.051	0.013	8.00E-05	TNFAIP8
rs6569457	6	126573579	T/G	0.184	-0.018	0.005	8.00E-05	TRMT11-CENPW
rs13194446	6	160610763	T/G	0.079	-0.027	0.007	8.00E-05	SLC22A1-SLC22A2
rs2526376	17	56427142	A/C	0.394	-0.015	0.004	9.00E-05	RNF43,TSPOAP1-AS1,SUPT4H1
rs9849022	3	41297523	G/T	0.477	-0.015	0.004	9.00E-05	ULK4
rs1166178	6	117534328	T/C	0.159	0.019	0.005	9.00E-05	RFX6-VGLL2
rs112628506	10	18461921	C/T	0.05	-0.033	0.009	1.00E-04	CACNB2
rs4503429	10	25045907	C/A	0.435	0.014	0.004	1.00E-04	ARHGAP21-PRTFDC1
rs12421951	11	17194104	T/C	0.043	-0.033	0.009	1.00E-04	PIK3C2A
rs4902778	14	70580236	C/T	0.439	0.014	0.004	1.00E-04	SLC8A3
rs28453685	15	60896778	C/T	0.226	-0.017	0.005	1.00E-04	RORA,RORA-AS1
rs45626947	19	11559489	C/T	0.05	0.032	0.009	1.00E-04	ELAVL3,PRKCSH
rs4951068	1	204318997	C/T	0.323	0.016	0.004	1.00E-04	PLEKHA6
rs3138159	1	6520024	C/A	0.072	-0.029	0.007	1.00E-04	TNFRSF25,ESPN
rs79024651	20	40008056	G/A	0.034	0.036	0.01	1.00E-04	EMILIN3-CHD6
rs6012542	20	47477706	C/T	0.329	0.015	0.004	1.00E-04	PREX1-ARFGEF2
rs2103653	20	8551344	G/A	0.36	-0.014	0.004	1.00E-04	PLCB1
rs71329073	21	35910350	G/T	0.11	0.023	0.006	1.00E-04	RCAN1
rs74815345	22	25165022	T/G	0.068	0.029	0.007	1.00E-04	TOP1P2,PIWIL3
rs137739	22	44037064	C/A	0.414	0.016	0.004	1.00E-04	EFCAB6
rs6432195	2	11590579	A/G	0.367	-0.014	0.004	1.00E-04	E2F6
rs10497599	2	183279530	G/A	0.13	0.021	0.005	1.00E-04	PDE1A
rs17215596	2	207932371	A/G	0.285	0.016	0.004	1.00E-04	CPO-KLF7
rs11719633	3	20402881	G/A	0.092	0.024	0.006	1.00E-04	LOC101927829-VENTXP7
rs55801703	3	21782561	T/G	0.221	-0.017	0.005	1.00E-04	ZNF385D
rs11915711	3	70506833	G/A	0.422	-0.015	0.004	1.00E-04	MDFIC2-FOXP1
rs12522730	5	4226713	C/T	0.224	0.018	0.005	1.00E-04	IRX1-LINC02114
rs10275721	7	69290396	T/G	0.055	-0.034	0.008	1.00E-04	AUTS2

SNPid	CHR	pos	Effect/other alleles	Effect allele Freq	beta	SE	pval	closest gene
rs45446698	7	99332948	T/G	0.04	0.035	0.009	1.00E-04	CYP3A7-CYP3A51P,CYP3A7,CYP3A7-CYP3A51P-CYP3A4
rs2069199	8	106326772	C/A	0.434	0.015	0.004	1.00E-04	ZFPM2,LRP12-ZFPM2
rs10105003	8	127654213	C/T	0.255	0.017	0.004	1.00E-04	LRATD2-PCAT1
rs117632084	8	89161084	G/A	0.07	0.029	0.007	1.00E-04	MMP16
rs34576091	9	33896077	A/G	0.102	-0.025	0.006	1.00E-04	UBE2R2
rs72820803	10	64761387	C/T	0.317	-0.018	0.004	1.1e-05	EGR2-NRBF2
rs484934	11	122021210	G/T	0.326	0.018	0.004	1.1e-05	MIRLET7A2,MIR10526,MIR100,MIR100HG
rs57257053	11	84292548	G/A	0.115	-0.025	0.006	1.1e-05	DLG2
rs12582526	12	53430509	C/T	0.198	0.021	0.005	1.1e-05	EIF4B
rs36160390	13	114447797	G/A	0.363	-0.017	0.004	1.1e-05	LINC00552,GRK1-LINC00552
rs556979	13	32225060	A/G	0.302	-0.018	0.004	1.1e-05	B3GLCT-RXFP2
rs4839566	1	111331000	C/A	0.218	0.02	0.005	1.1e-05	KCNA3-CD53
rs3124989	1	13798945	T/C	0.399	0.017	0.004	1.1e-05	LRRC38,PRAMEF20-LRRC38
rs12757371	1	203126010	C/T	0.062	0.035	0.008	1.1e-05	ADORA1
rs77037886	1	210313031	A/G	0.181	-0.022	0.005	1.1e-05	SYT14
rs78720247	1	227521432	G/A	0.106	-0.025	0.006	1.1e-05	CDC42BPA-ZNF678
rs6686661	1	46638537	T/C	0.393	0.017	0.004	1.1e-05	TSPAN1,P3R3URF,LOC110117498-PIK3R3,PIK3R3
rs6748298	2	164833680	A/G	0.334	0.017	0.004	1.1e-05	FIGN-GRB14
rs2601097	2	169291012	G/T	0.48	-0.015	0.004	1.1e-05	STK39-CERS6
rs72854468	2	28330071	A/G	0.07	-0.032	0.007	1.1e-05	BABAM2
rs143867864	3	49435706	C/T	0.054	0.035	0.008	1.1e-05	RHOA
rs17499351	5	88470330	T/C	0.048	0.038	0.008	1.1e-05	MEF2C-AS1
rs112796887	6	128190534	T/C	0.028	0.048	0.011	1.1e-05	THEMIS
rs1923170	6	24379225	A/G	0.106	-0.025	0.006	1.1e-05	DCDC2
rs7774614	6	50260308	G/T	0.085	-0.029	0.007	1.1e-05	DEFB112-TFAP2D
rs118033050	7	150586206	G/T	0.078	0.029	0.007	1.1e-05	AOC1-KCNH2
rs1064807	8	26236978	C/T	0.295	0.019	0.004	1.1e-05	BNIP3L,SDAD1P1
rs1815303	9	36014678	A/G	0.152	-0.023	0.005	1.1e-05	OR2S2-RECK
rs57769625	9	86640705	C/T	0.274	-0.019	0.004	1.1e-05	RMI1-LOC101927575
rs11224409	11	100592656	T/C	0.103	-0.029	0.006	1.1e-06	ARHGAP42
rs4544018	11	58103667	T/C	0.377	-0.019	0.004	1.1e-06	OR10W1-OR5B17
rs7111257	11	9930813	A/G	0.252	-0.021	0.004	1.1e-06	SBF2,LOC101928008
rs2162734	12	20359670	T/C	0.481	0.019	0.004	1.1e-06	LINC02468-PDE3A
rs12946717	17	16238376	G/A	0.491	0.018	0.004	1.1e-06	PIGL-CENPV
rs71372388	17	26911529	C/A	0.105	0.029	0.006	1.1e-06	SPAG5
rs5762497	22	28546346	T/G	0.131	-0.027	0.006	1.1e-06	TTC28
rs35877502	2	175144942	A/G	0.324	-0.02	0.004	1.1e-06	OLA1-LINC01305
rs11124941	2	23444447	T/C	0.293	-0.02	0.004	1.1e-06	LINC01884-KLHL29

SNPid	CHR	pos	Effect/other alleles	Effect allele Freq	beta	SE	pval	closest gene
rs9819362	3	168878674	A/G	0.463	0.019	0.004	1.1e-06	MECOM
rs34297584	4	111240278	G/A	0.019	-0.065	0.013	1.1e-06	ELOVL6-ENPEP
rs455660	5	55816888	T/C	0.19	-0.024	0.005	1.1e-06	C5orf67
rs79189747	6	138874625	C/T	0.029	0.053	0.011	1.1e-06	NHSL1
rs2107595	7	19049388	G/A	0.157	-0.026	0.005	1.1e-06	HDAC9-TWIST1
rs17123349	12	49223541	A/G	0.093	-0.035	0.006	1.1e-07	DDX23,DDX23,CACNB3
rs11852268	15	85667983	T/G	0.035	-0.053	0.01	1.1e-07	PDE8A
rs6499323	16	70624483	A/G	0.273	0.021	0.004	1.1e-07	IL34
rs284237	1	10678610	G/A	0.125	0.029	0.006	1.1e-07	PEX14
rs78940046	2	159410213	A/C	0.278	0.022	0.004	1.1e-07	PKP4
rs112350050	7	131197667	C/T	0.085	0.035	0.007	1.1e-07	PODXL
rs79598313	1	27284913	C/T	0.022	-0.073	0.012	1.1e-08	KDF1
rs2358891	2	175558804	G/A	0.233	-0.026	0.004	1.1e-08	WIPF1-H3P6
rs9986272	5	88120326	C/T	0.065	0.042	0.007	1.1e-08	MEF2C
rs34369885	13	30115634	G/A	0.086	-0.04	0.007	1.1e-09	SLC7A1
rs2208589	20	47408414	A/G	0.218	-0.027	0.005	1.1e-09	PREX1
rs2044065	5	68755613	C/T	0.022	-0.075	0.013	1.1e-09	MARVELD2-LOC101928924
rs830460	8	76967792	A/G	0.341	-0.024	0.004	1.1e-09	HNF4G-LINC01111
rs1469249	5	113837198	G/A	0.205	-0.03	0.005	1.1e-10	LOC101927078
rs9343824	6	79497569	A/G	0.337	-0.026	0.004	1.1e-10	MIR10524-IRAK1BP1
rs56743174	3	168814206	G/A	0.074	0.049	0.007	1.1e-11	MECOM
rs7798740	7	139462162	T/C	0.158	0.036	0.005	1.1e-12	HIPK2
rs642803	11	65560620	C/T	0.463	0.032	0.004	1.1e-17	OVOL1-AS1,OVOL1
rs4277405	17	61548918	C/T	0.375	0.042	0.004	1.1e-27	CYB561-ACE
rs72831343	10	63515681	T/G	0.14	0.086	0.005	1.1e-58	CABCOCO1
rs2504016	10	33188434	A/G	0.058	-0.032	0.008	1.2e-05	ITGB1,SNORA86,CCDC7-ITGB1
rs204937	11	8280938	C/T	0.44	0.017	0.004	1.2e-05	LMO1
rs73207624	12	112674718	C/T	0.036	-0.042	0.01	1.2e-05	HECTD4
rs113893579	12	115472344	C/T	0.039	0.043	0.009	1.2e-05	TBX3-MED13L
rs34135607	17	511507	A/G	0.192	-0.02	0.005	1.2e-05	VPS53
rs7216355	17	64474993	T/C	0.363	0.017	0.004	1.2e-05	PRKCA
rs2277964	19	10104154	G/T	0.214	0.02	0.005	1.2e-05	COL5A3
rs11586623	1	45758511	A/G	0.106	-0.027	0.006	1.2e-05	ZSWIM5-LINC01144
rs136829	22	40046176	T/C	0.184	-0.02	0.005	1.2e-05	CACNA1I
rs11676014	2	113558886	A/G	0.431	0.016	0.004	1.2e-05	IL1A-IL1B
rs1642922	2	225305832	C/T	0.162	0.023	0.005	1.2e-05	FAM124B-CUL3
rs112812240	2	43275371	C/T	0.036	-0.043	0.01	1.2e-05	LINC01819-LINC02580
rs12639495	3	35075831	T/C	0.252	-0.019	0.004	1.2e-05	LOC101928135
rs6843197	4	156699054	G/A	0.163	0.023	0.005	1.2e-05	GUCY1B1
rs13139534	4	2683400	A/C	0.073	-0.032	0.007	1.2e-05	FAM193A
rs35716337	4	95862384	C/T	0.257	0.019	0.004	1.2e-05	BMPR1B

SNPid	CHR	pos	Effect/other alleles	Effect allele Freq	beta	SE	pval	closest gene
rs149309025	6	119154346	C/T	0.444	-0.017	0.004	1.2e-05	MCM9
rs9382154	6	13496604	C/T	0.232	0.019	0.004	1.2e-05	GFOD1-SIRT5
rs4895670	6	145664768	T/C	0.056	0.035	0.008	1.2e-05	UTRN-EPM2A
rs7769686	6	163199467	A/G	0.118	0.026	0.006	1.2e-05	PACRG
rs12199806	6	27435943	A/C	0.071	0.043	0.01	1.2e-05	ZNF184
rs17254865	6	62540482	T/C	0.209	0.02	0.005	1.2e-05	KHDRBS2
rs10828545	10	18629603	G/A	0.359	0.019	0.004	1.2e-06	CACNB2
rs3741042	11	10631247	C/T	0.484	-0.018	0.004	1.2e-06	MRVI1
rs61993994	14	60491133	G/A	0.155	0.024	0.005	1.2e-06	LRRC9
rs11639246	15	90128834	G/A	0.488	0.018	0.004	1.2e-06	TICRR
rs8080754	17	62377645	C/T	0.379	-0.019	0.004	1.2e-06	TEX2-PECAM1
rs3128339	1	2046938	G/T	0.397	-0.019	0.004	1.2e-06	PRKCZ
rs11120771	1	6878371	T/G	0.093	0.032	0.006	1.2e-06	CAMTA1
rs2391972	1	99533151	A/G	0.241	0.021	0.004	1.2e-06	LOC100129620
rs6090921	20	47501776	G/A	0.178	0.023	0.005	1.2e-06	PREX1-ARFGEF2
rs7568495	2	200066671	G/T	0.444	-0.019	0.004	1.2e-06	LINC01923-SATB2
rs62133080	2	37230026	C/T	0.194	0.023	0.005	1.2e-06	HEATR5B
rs16897819	5	67716281	T/G	0.072	-0.035	0.007	1.2e-06	PIK3R1-LINC02198
rs2745353	6	127452935	C/T	0.479	0.019	0.004	1.2e-06	RSPO3
rs7826828	8	134229883	C/A	0.422	-0.019	0.004	1.2e-06	CCN4
rs11136424	8	1717036	G/A	0.266	-0.021	0.004	1.2e-06	CLN8
rs28380191	8	91122146	T/G	0.298	-0.02	0.004	1.2e-06	CALB1-LINC00534
rs17885785	11	2167850	C/T	0.196	0.025	0.005	1.2e-07	INS-IGF2,IGF2-AS,IGF2,INS-IGF2
rs57340570	13	30155800	C/A	0.087	-0.036	0.007	1.2e-07	SLC7A1
rs13387221	2	44135314	G/A	0.18	0.024	0.005	1.2e-07	LRPPRC
rs1048070	9	14735053	T/C	0.468	-0.021	0.004	1.2e-07	FREM1
rs11188112	10	96666699	T/C	0.221	-0.025	0.005	1.2e-08	CYP2C19-CYP2C9
rs113602013	18	57919710	G/A	0.3	-0.023	0.004	1.2e-08	PMAIP1-MC4R
rs116044797	5	32886323	T/C	0.024	0.068	0.012	1.2e-08	NPR3-LINC02120
rs34315310	2	164874617	T/C	0.056	0.051	0.008	1.2e-09	FIGN-GRB14
rs16932756	9	35921694	A/G	0.115	-0.035	0.006	1.2e-09	SPAAR-OR2S2
rs12731208	1	6676485	T/G	0.349	-0.026	0.004	1.2e-10	PHF13
rs28401365	8	81440788	G/A	0.198	0.03	0.005	1.2e-10	ZBTB10,ZBTB10-ZNF704
rs11066308	12	112881996	A/G	0.083	0.047	0.007	1.2e-11	PTPN11
rs8093196	18	42113270	G/T	0.33	-0.028	0.004	1.2e-12	LINC01478,LINC01601
rs971791	1	113026534	G/A	0.236	-0.036	0.004	1.2e-16	WNT2B
rs4094474	11	48924889	C/T	0.142	0.047	0.005	1.2e-18	OR4A47-TRIM49B
rs10770059	11	9770910	T/C	0.346	-0.039	0.004	1.2e-23	SWAP70
rs6039211	20	8616588	A/G	0.365	0.043	0.004	1.2e-28	PLCB1
rs1573643	15	91420973	T/C	0.33	-0.054	0.004	1.2e-40	FURIN
rs3918226	7	150690176	C/T	0.074	-0.111	0.007	1.2e-57	NOS3

SNPid	CHR	pos	Effect/other alleles	Effect allele Freq	beta	SE	pval	closest gene
rs3008362	10	135045035	T/C	0.102	-0.026	0.006	1.3e-05	UTF1
rs56211871	11	13067483	T/C	0.174	-0.021	0.005	1.3e-05	RASSF10-ARNTL
rs11820339	11	8632456	C/T	0.135	-0.025	0.006	1.3e-05	TRIM66,STK33-TRIM66
rs74546467	12	20571444	G/A	0.055	0.035	0.008	1.3e-05	PDE3A
rs491907	15	50908697	A/G	0.492	0.017	0.004	1.3e-05	TRPM7
rs16953806	16	54681404	G/A	0.175	0.022	0.005	1.3e-05	LINC02140-LOC101927480
rs56349113	17	78639415	G/A	0.082	0.029	0.007	1.3e-05	RPTOR
rs3816046	19	46118127	C/T	0.323	-0.018	0.004	1.3e-05	EML2
rs209956	20	52722856	G/A	0.27	-0.019	0.004	1.3e-05	BCAS1-CYP24A1
rs116921259	21	16555091	G/A	0.08	-0.03	0.007	1.3e-05	NRIP1-USP25
rs139072358	5	68204354	T/C	0.105	-0.026	0.006	1.3e-05	PIK3R1-LINC02198
rs1094382	6	11501909	T/C	0.47	0.017	0.004	1.3e-05	NEDD9-TMEM170B
rs2745593	6	1603402	G/A	0.46	0.016	0.004	1.3e-05	FOXCUT,FOXF2-FOXCUT
rs146742336	6	26977628	G/A	0.032	-0.046	0.011	1.3e-05	LINC00240
rs77428836	6	95163148	G/T	0.232	-0.019	0.004	1.3e-05	TSG1-MANEA-DT
rs113825498	7	131535589	A/G	0.294	-0.017	0.004	1.3e-05	PODXL-LOC101928782
rs74458426	7	27134638	C/T	0.049	0.037	0.009	1.3e-05	HOTAIRM1,HOXA1
rs11781699	8	118863061	T/C	0.178	-0.021	0.005	1.3e-05	EXT1
rs7832054	8	141620713	C/A	0.103	-0.026	0.006	1.3e-05	AGO2
rs112270518	9	136471660	G/A	0.11	0.026	0.006	1.3e-05	FAM163B
rs10744774	12	112090022	C/A	0.159	-0.024	0.005	1.3e-06	BRAP
rs11048454	12	26460045	C/A	0.236	-0.021	0.004	1.3e-06	SSPN-ITPR2
rs4923919	15	42208041	G/A	0.305	-0.019	0.004	1.3e-06	EHD4
rs16953013	18	48780405	G/A	0.105	-0.03	0.006	1.3e-06	MEX3C-LINC01630
rs968699	19	11289826	C/T	0.298	-0.02	0.004	1.3e-06	KANK2
rs1558118	19	2232083	G/T	0.078	-0.036	0.007	1.3e-06	DOT1L,SF3A2,MIR1227,MIR6789,PLEKHJ1
rs7248960	19	32604309	C/T	0.298	0.02	0.004	1.3e-06	LINC01782-ZNF507
rs10495160	1	221135961	C/T	0.091	0.032	0.006	1.3e-06	HLX-C1orf140
rs6067424	20	48990945	T/C	0.283	0.02	0.004	1.3e-06	LINC01271-PTPN1
rs12479678	20	57552375	C/T	0.304	0.02	0.004	1.3e-06	NELFCD,GNAS-NELFCD
rs9374286	6	112075257	C/T	0.417	0.019	0.004	1.3e-06	FYN
rs41294854	6	135401371	C/T	0.1	-0.03	0.006	1.3e-06	HBS1L-MYB
rs9369946	6	50225528	A/C	0.461	0.018	0.004	1.3e-06	DEFB112-TFAP2D
rs57832775	9	109075787	C/T	0.072	0.035	0.007	1.3e-06	TMEM38B-MIR8081
rs73413011	11	1941481	T/C	0.232	0.024	0.004	1.3e-07	TNNT3
rs775307	12	67660415	G/A	0.285	0.022	0.004	1.3e-07	CAND1,LOC102724421-CAND1
rs630943	13	110877046	A/G	0.266	0.023	0.004	1.3e-07	COL4A1
rs12920774	16	24830750	T/C	0.192	0.025	0.005	1.3e-07	TNRC6A
rs62065366	17	46817759	C/T	0.184	-0.026	0.005	1.3e-07	HOXB13-TTLL6
rs11880558	19	12581063	A/G	0.231	-0.024	0.005	1.3e-07	ZNF709

SNPid	CHR	pos	Effect/other alleles	Effect allele Freq	beta	SE	pval	closest gene
rs111805423	2	37248924	G/T	0.289	-0.022	0.004	1.3e-07	HEATR5B
rs2680661	3	53742148	T/G	0.237	-0.022	0.004	1.3e-07	CACNA1D
rs17631367	8	17200201	A/C	0.159	0.028	0.005	1.3e-07	MTMR7
rs2393455	10	60374898	C/A	0.429	-0.022	0.004	1.3e-08	BICC1
rs11105273	12	89787787	T/C	0.152	0.029	0.005	1.3e-08	DUSP6-POC1B
rs9317097	13	23170374	C/T	0.182	-0.028	0.005	1.3e-08	LINC00540-LINC00621
rs323713	1	43279132	C/T	0.281	-0.023	0.004	1.3e-08	ERMAP,SVBP
rs12615509	2	27098766	G/A	0.393	0.022	0.004	1.3e-08	DPYSL5
rs9369425	6	43810974	G/A	0.293	-0.023	0.004	1.3e-08	LINC02537,VEGFA-LINC02537
rs36037977	20	4426532	G/A	0.052	0.053	0.008	1.3e-09	ADRA1D-PRNP
rs2441466	2	61373930	C/T	0.29	0.025	0.004	1.3e-09	LOC339803,C2orf74
rs8014182	14	103859962	C/T	0.125	0.037	0.006	1.3e-10	MARK3
rs6728849	2	164732109	T/C	0.391	-0.024	0.004	1.3e-10	FIGN-GRB14
rs1843091	15	74619537	T/C	0.294	0.029	0.004	1.3e-12	CCDC33
rs17199964	4	102707791	G/A	0.075	0.049	0.007	1.3e-12	BANK1,FLJ20021-BANK1
rs3764769	2	43732958	C/T	0.257	0.032	0.004	1.3e-14	THADA
rs76833657	17	7781020	G/A	0.076	-0.059	0.007	1.3e-16	NAA38
rs17147691	11	55524737	T/G	0.382	-0.017	0.004	1.4e-05	OR4C6-OR5D13
rs6582280	12	41629042	T/G	0.351	0.017	0.004	1.4e-05	PDZRN4
rs7142232	14	50847492	C/T	0.405	-0.016	0.004	1.4e-05	CDKL1
rs61984496	14	59790745	G/A	0.244	0.019	0.004	1.4e-05	DAAM1
rs140646549	15	43680031	G/A	0.026	-0.052	0.012	1.4e-05	TUBGCP4
rs1044355	15	75137064	G/A	0.076	0.032	0.007	1.4e-05	SCAMP2,ULK3,MIR6882
rs7191540	16	60615912	T/C	0.226	0.02	0.005	1.4e-05	NPAP1L-MIR4426
rs7215084	17	3880148	C/T	0.483	-0.016	0.004	1.4e-05	LINC01975,ATP2A3-LINC01975
rs2715831	17	67527469	T/C	0.383	-0.016	0.004	1.4e-05	MAP2K6
rs12709693	18	20256891	T/C	0.31	-0.019	0.004	1.4e-05	CTAGE1-LOC101927571
rs7248236	19	49647058	G/A	0.397	0.017	0.004	1.4e-05	PPFIA3
rs311464	1	25419442	A/G	0.252	-0.019	0.004	1.4e-05	MIR4425-SYF2
rs1579406	2	218847092	C/A	0.115	0.026	0.006	1.4e-05	LOC105373878
rs35254732	3	157799168	G/A	0.127	0.024	0.006	1.4e-05	SLC66A1L-SHOX2
rs267243	3	45622893	G/A	0.46	-0.016	0.004	1.4e-05	LARS2-LIMD1
rs309768	4	177452214	A/G	0.378	0.017	0.004	1.4e-05	SPCS3-VEGFC
rs4440352	5	114220256	T/C	0.058	0.034	0.008	1.4e-05	LOC101927078-TRIM36
rs2905617	5	137035117	T/C	0.231	0.019	0.004	1.4e-05	KLHL3
rs2746187	6	127011064	A/G	0.188	-0.022	0.005	1.4e-05	MIR588-RSPO3
rs4515482	7	107072681	A/C	0.224	0.02	0.005	1.4e-05	COG5
rs78079625	7	39221793	T/G	0.101	0.026	0.006	1.4e-05	POU6F2
rs143524414	7	98965615	G/A	0.063	0.033	0.008	1.4e-05	ARPC1A,ARPC1A-ARPC1B
rs4871406	8	124670101	C/T	0.204	-0.021	0.005	1.4e-05	KLHL38,KLHL38-

SNPid	CHR	pos	Effect/other alleles	Effect allele Freq	beta	SE	pval	closest gene
								ANXA13
rs34666150	12	97690200	C/T	0.068	0.034	0.007	1.4e-06	NEDD1-RMST
rs34257057	15	64419374	G/T	0.063	-0.039	0.008	1.4e-06	SNX1
rs731839	19	33899065	G/A	0.342	0.019	0.004	1.4e-06	PEPD
rs238196	20	47898217	G/A	0.207	0.022	0.005	1.4e-06	ZNFX1,SNORD12C,SNORD12B,SNORD12,ZFAS1
rs2835336	21	37709639	G/A	0.477	-0.018	0.004	1.4e-06	MORC3
rs8140401	22	29429955	G/A	0.066	0.035	0.007	1.4e-06	ZNRF3-AS1,ZNRF3
rs2357461	2	193769529	A/G	0.498	-0.018	0.004	1.4e-06	PCGEM1-LINC01821
rs2971882	2	54861172	A/G	0.482	-0.019	0.004	1.4e-06	SPTBN1
rs1521955	2	79103513	C/T	0.475	-0.018	0.004	1.4e-06	LOC105374820-REG3G
rs3774428	3	53565829	G/T	0.228	-0.022	0.004	1.4e-06	CACNA1D
rs17032400	4	103190229	T/C	0.456	-0.019	0.004	1.4e-06	SLC39A8
rs28732167	6	32031904	G/A	0.048	-0.056	0.012	1.4e-06	TNXB
rs72823015	10	115786611	G/T	0.124	-0.03	0.006	1.4e-07	NHLRC2-ADRB1
rs11818697	10	32273277	A/G	0.29	-0.022	0.004	1.4e-07	ARHGAP12-KIF5B
rs11607954	11	1714688	T/C	0.459	-0.02	0.004	1.4e-07	KRTAP5-6,FAM99B-KRTAP5-6
rs739837	12	48238221	G/T	0.474	-0.019	0.004	1.4e-07	VDR
rs227426	14	70456664	G/T	0.442	-0.02	0.004	1.4e-07	SMOC1
rs7190835	16	84100628	A/G	0.301	0.022	0.004	1.4e-07	MBTPS1
rs12039668	1	89098380	T/C	0.345	-0.021	0.004	1.4e-07	PKN2-AS1
rs16823201	2	183283295	A/G	0.054	-0.044	0.008	1.4e-07	PDE1A
rs1213404	5	111103018	G/A	0.413	0.02	0.004	1.4e-07	NREP
rs71484411	11	56860396	G/T	0.063	0.043	0.008	1.4e-08	OR5AK4P-LRRC55
rs12370770	12	112277084	C/T	0.057	-0.046	0.008	1.4e-08	MAPKAPK5,MAPKAPK5-AS1,ALDH2-MAPKAPK5-AS1
rs60436973	1	89403729	A/C	0.106	0.035	0.006	1.4e-08	KYAT3
rs11766944	7	1888051	G/A	0.184	0.028	0.005	1.4e-08	MIR4655,MAD1L1
rs2131570	3	14906734	C/T	0.473	0.023	0.004	1.4e-09	FGD5
rs6441207	3	158282459	C/T	0.407	-0.023	0.004	1.4e-09	LOC100996447
rs6934891	6	22139729	G/A	0.428	-0.023	0.004	1.4e-09	CASC15,NBAT1
rs80090905	11	47800628	C/T	0.054	-0.056	0.008	1.4e-11	NUP160
rs77498573	11	48350646	G/T	0.059	-0.054	0.008	1.4e-11	OR4C3,OR4C3-OR4C45
rs11955050	5	114133676	G/A	0.424	0.027	0.004	1.4e-12	LOC101927078-TRIM36
rs708111	1	228191365	G/A	0.488	-0.03	0.004	1.4e-15	WNT3A,LOC107985355-WNT3A
rs12570727	10	18425519	G/A	0.387	-0.036	0.004	1.4e-20	CACNB2,SLC39A12-CACNB2
rs10437655	11	47391948	G/A	0.387	-0.038	0.004	1.4e-21	SPI1
rs2994387	10	82017686	G/A	0.487	0.016	0.004	1.5e-05	LOC100130698,LOC100130698-MAT1A
rs75259664	12	112815698	A/G	0.072	0.03	0.007	1.5e-05	HECTD4
rs17124559	12	50635918	T/C	0.042	0.04	0.009	1.5e-05	LIMA1

SNPid	CHR	pos	Effect/other alleles	Effect allele Freq	beta	SE	pval	closest gene
rs12816187	12	51733951	A/C	0.424	0.017	0.004	1.5e-05	CELA1
rs11641362	16	85335951	G/T	0.324	0.018	0.004	1.5e-05	MIR5093,LINC00311-MIR5093
rs56347129	17	46485443	C/T	0.025	0.049	0.012	1.5e-05	SKAP1
rs113621438	17	64250245	G/A	0.026	-0.051	0.012	1.5e-05	APOH-PRKCA
rs12961750	18	58363678	G/T	0.492	0.016	0.004	1.5e-05	MC4R-CDH20
rs667357	1	20872694	C/T	0.372	-0.017	0.004	1.5e-05	MUL1-FAM43B
rs1215502	1	90093015	T/C	0.481	-0.016	0.004	1.5e-05	LRRC8C-DT
rs6544325	2	40584730	C/T	0.423	-0.016	0.004	1.5e-05	SLC8A1
rs6775453	3	68776331	T/C	0.371	0.017	0.004	1.5e-05	TAFA4,TAFA1-TAFA4
rs10247712	7	122896963	A/G	0.297	-0.017	0.004	1.5e-05	SLC13A1-IQUB
rs56008118	9	112695646	A/G	0.045	0.039	0.009	1.5e-05	PALM2AKAP2
rs77749960	9	98777130	T/C	0.113	0.026	0.006	1.5e-05	ERCC6L2,LINC00092
rs12797544	11	57049791	A/G	0.308	0.019	0.004	1.5e-06	APLNR-TNKS1BP1
rs1975822	17	18244770	C/T	0.429	-0.018	0.004	1.5e-06	MIR6778,SHMT1
rs12936934	17	7500765	C/T	0.196	0.022	0.005	1.5e-06	FXR2
rs9661078	1	11936324	A/C	0.412	-0.018	0.004	1.5e-06	NPPB-RNU5E-1
rs10187334	2	69079165	A/G	0.32	0.019	0.004	1.5e-06	ARHGAP25-BMP10
rs1475974	4	2942073	T/C	0.329	0.019	0.004	1.5e-06	NOP14-AS1,NOP14
rs6532091	4	89877367	C/T	0.339	-0.019	0.004	1.5e-06	FAM13A
rs17155270	7	27504332	A/G	0.26	0.02	0.004	1.5e-06	EVX1-HIBADH
rs10506977	12	90318459	T/C	0.14	0.027	0.005	1.5e-07	ATP2B1-AS1-LINC02399
rs93177	13	72364711	A/C	0.106	-0.033	0.007	1.5e-07	DACH1
rs4792819	17	43247957	A/C	0.291	-0.021	0.004	1.5e-07	HEXIM2,LOC105371795
rs11928547	3	124577290	T/C	0.411	0.019	0.004	1.5e-07	ITGB5
rs6864072	5	14093588	C/T	0.389	-0.02	0.004	1.5e-07	DNAH5-TRIO
rs11751804	6	51145594	A/G	0.236	-0.024	0.004	1.5e-07	TFAP2B-PKHD1
rs11264763	1	153948705	C/T	0.288	0.024	0.004	1.5e-08	CREB3L4,JTB
rs62188801	2	162744546	T/C	0.067	0.042	0.007	1.5e-08	SLC4A10
rs9751478	2	208509582	T/C	0.458	-0.021	0.004	1.5e-08	METTL21A-LINC01857
rs34665498	16	337691	C/T	0.165	0.03	0.005	1.5e-09	AXIN1,ARHGDIG,PDIA2
rs6532414	4	77401195	G/A	0.343	0.024	0.004	1.5e-09	SHROOM3
rs2525887	11	2027064	C/T	0.411	-0.025	0.004	1.5e-10	H19-IGF2
rs9302885	17	76799898	A/G	0.448	0.024	0.004	1.5e-10	USP36
rs72912175	11	46710111	A/G	0.117	-0.04	0.006	1.5e-11	ARHGAP1
rs3519	12	112598321	G/A	0.081	0.047	0.007	1.5e-11	HECTD4,MIR6861
rs4110517	10	96650328	A/G	0.208	-0.034	0.005	1.5e-13	CYP2C19-CYP2C9
rs4384926	3	133995065	G/A	0.438	-0.028	0.004	1.5e-13	RYK-LINC02004
rs35516283	4	26806736	C/T	0.401	-0.029	0.004	1.5e-14	TBC1D19-STIM2-AS1
rs7803355	7	7221775	C/T	0.114	-0.047	0.006	1.5e-15	C1GALT1,LOC100131257-C1GALT1
rs180940	10	115722411	A/G	0.331	-0.035	0.004	1.5e-18	NHLRC2-ADRB1

SNPid	CHR	pos	Effect/other alleles	Effect allele Freq	beta	SE	pval	closest gene
rs11039520	11	48130794	A/G	0.138	0.053	0.005	1.5e-22	PTPRJ
rs7942556	11	100534632	T/C	0.277	0.051	0.004	1.5e-34	CNTN5-LOC100128386
rs11813708	10	129881970	C/A	0.048	-0.039	0.009	1.6e-05	PTPRE
rs116892553	10	63601824	G/A	0.036	-0.044	0.01	1.6e-05	CABCOCO1-ARID5B
rs4554901	11	133400279	C/T	0.137	0.023	0.005	1.6e-05	OPCML
rs17308782	11	26134446	A/G	0.092	-0.028	0.006	1.6e-05	LUZP2-ANO3
rs12822167	12	24566725	G/T	0.388	0.017	0.004	1.6e-05	SOX5
rs73130641	12	41864755	G/A	0.037	-0.041	0.01	1.6e-05	PDZRN4
rs4759229	12	56474480	A/G	0.347	-0.017	0.004	1.6e-05	ERBB3
rs7149559	14	90891435	G/A	0.21	-0.02	0.005	1.6e-05	CALM1-LINC02317
rs2305761	19	51015869	G/A	0.268	0.019	0.004	1.6e-05	JOSD2,LRRC4B,ASPDH
rs603160	1	184802062	A/G	0.08	-0.03	0.007	1.6e-05	NIBAN1
rs4655461	1	213864971	G/A	0.266	-0.018	0.004	1.6e-05	RPS6KC1-PROX1-AS1
rs6707047	2	145503373	A/G	0.248	-0.019	0.004	1.6e-05	TEX41
rs35766826	2	26827290	G/A	0.022	-0.053	0.012	1.6e-05	CIB4
rs17189638	2	54306778	G/T	0.06	-0.034	0.008	1.6e-05	ACYP2
rs6729083	2	64680967	A/G	0.323	-0.017	0.004	1.6e-05	LGALSL,MIR4433B-LGALSL
rs2280312	2	86072076	G/A	0.197	-0.02	0.005	1.6e-05	ST3GAL5
rs6763380	3	138179328	C/T	0.468	0.016	0.004	1.6e-05	ESYT3
rs6846180	4	163974516	A/G	0.42	0.016	0.004	1.6e-05	FSTL5-MIR4454
rs1328126	6	97366760	A/G	0.241	-0.018	0.004	1.6e-05	KLHL32
rs2529185	7	101346222	C/T	0.136	-0.024	0.005	1.6e-05	MYL10-CUX1
rs55975206	7	122135794	A/G	0.307	-0.018	0.004	1.6e-05	CADPS2
rs10890658	11	107038741	C/T	0.397	-0.019	0.004	1.6e-06	GUCY1A2-CWF19L2
rs1950764	14	68343363	G/A	0.168	0.023	0.005	1.6e-06	RAD51B
rs7178018	15	25925675	G/A	0.358	-0.018	0.004	1.6e-06	ATP10A
rs11654557	17	47390136	A/G	0.085	0.032	0.007	1.6e-06	ZNF652
rs77397926	2	142341391	T/C	0.392	0.018	0.004	1.6e-06	LRP1B
rs56370736	5	72654319	C/T	0.058	-0.039	0.008	1.6e-06	LINC02230-FOXD1
rs34587258	6	162945580	T/C	0.118	0.027	0.006	1.6e-06	PRKN
rs11187118	10	94412186	C/T	0.087	-0.036	0.007	1.6e-07	KIF11
rs7932807	11	16438411	A/G	0.391	0.02	0.004	1.6e-07	SOX6
rs2283454	15	92700274	G/T	0.28	-0.022	0.004	1.6e-07	SLCO3A1
rs2131431	17	1294895	A/C	0.267	-0.023	0.004	1.6e-07	YWHAE
rs2815116	6	39187886	G/A	0.297	0.022	0.004	1.6e-07	KCNK5
rs62491907	8	26439185	C/T	0.139	0.029	0.005	1.6e-07	DPYSL2
rs3819536	12	2436998	G/A	0.294	-0.023	0.004	1.6e-08	CACNA1C
rs318699	19	11501240	A/G	0.354	-0.023	0.004	1.6e-08	RGL3,EPOR-RGL3
rs4811058	20	49011501	T/C	0.322	0.023	0.004	1.6e-08	LINC01271-PTPN1
rs2271834	2	69009304	C/T	0.418	0.021	0.004	1.6e-08	ARHGAP25
rs2240736	17	59485393	C/T	0.272	-0.028	0.004	1.6e-11	C17orf82,TBX2

SNPid	CHR	pos	Effect/other alleles	Effect allele Freq	beta	SE	pval	closest gene
rs11229165	11	55112643	T/C	0.13	0.042	0.006	1.6e-14	OR4A16,OR4A16-OR4A15
rs6062533	20	62429101	G/A	0.229	0.033	0.004	1.6e-14	ZBTB46
rs2977324	8	76716737	T/G	0.295	-0.032	0.004	1.6e-14	HNF4G-LINC01111
rs7942337	11	76465272	C/T	0.215	0.02	0.005	1.7e-05	GUCY2EP-TSKU
rs9935845	16	51847798	G/A	0.458	0.017	0.004	1.7e-05	LINC01571-C16orf97
rs117428269	17	46093977	G/A	0.035	-0.043	0.01	1.7e-05	CDK5RAP3-COPZ2
rs1787846	18	3606272	C/T	0.242	0.019	0.004	1.7e-05	DLGAP1,DLGAP1-AS2
rs1646782	18	51405662	A/G	0.153	0.023	0.005	1.7e-05	LINC01919-MBD2
rs11247712	1	28229681	C/T	0.371	0.017	0.004	1.7e-05	RPA2
rs12083807	1	29285224	G/A	0.071	-0.031	0.007	1.7e-05	EPB41
rs1925341	1	67115084	A/G	0.292	-0.018	0.004	1.7e-05	SGIP1
rs12145044	1	93524045	T/G	0.047	0.038	0.009	1.7e-05	DIPK1A-MTF2
rs2918594	2	145627269	A/G	0.067	-0.032	0.007	1.7e-05	TEX41
rs6432705	2	162779958	T/C	0.445	-0.016	0.004	1.7e-05	SLC4A10
rs35430985	3	185488303	C/A	0.271	-0.018	0.004	1.7e-05	MIR548AQ,IGF2BP2
rs304077	3	4534629	A/C	0.388	0.017	0.004	1.7e-05	ITPR1,ITPR1-DT
rs4377576	4	21825726	T/C	0.482	-0.016	0.004	1.7e-05	KCNIP4
rs34498262	5	115330494	G/A	0.387	-0.017	0.004	1.7e-05	LVRN
rs62387979	5	157320013	C/T	0.08	-0.029	0.007	1.7e-05	CLINT1-LINC02227
rs202804	5	157630569	C/T	0.137	0.023	0.006	1.7e-05	CLINT1-LINC02227
rs2906872	7	97449546	C/A	0.212	0.02	0.005	1.7e-05	TAC1-ASNS
rs4750447	10	14037028	A/G	0.246	0.021	0.004	1.7e-06	FRMD4A
rs1999710	13	110492729	T/C	0.318	0.019	0.004	1.7e-06	IRS2-LINC00396
rs885064	16	88627727	T/C	0.254	-0.02	0.004	1.7e-06	ZC3H18-AS1
rs1937260	1	234131146	G/A	0.231	0.022	0.004	1.7e-06	SLC35F3
rs4660214	1	39731550	T/C	0.204	-0.022	0.005	1.7e-06	MACF1
rs3749262	3	131624097	G/A	0.27	-0.02	0.004	1.7e-06	CPNE4
rs4712955	6	25684279	A/G	0.46	-0.019	0.004	1.7e-06	SCGN
rs2075801	6	31728267	G/A	0.138	0.032	0.006	1.7e-06	SAPCD1,SAPCD1-AS1,MSH5,MSH5-SAPCD1
rs2224951	6	96493143	G/A	0.39	0.018	0.004	1.7e-06	FUT9
rs13287525	9	128219180	T/C	0.397	0.019	0.004	1.7e-06	MAPKAP1
rs1906469	10	63006098	T/G	0.128	-0.028	0.006	1.7e-07	LINC00845-TMEM26
rs74179972	19	11296830	A/G	0.076	-0.037	0.007	1.7e-07	KANK2
rs6693009	1	8743710	A/G	0.377	-0.02	0.004	1.7e-07	RERE
rs62190863	2	208845666	A/G	0.128	0.029	0.006	1.7e-07	PLEKHM3
rs12638862	3	169477506	A/G	0.26	0.022	0.004	1.7e-07	TERC,MECOM-TERC
rs78909293	5	148335250	T/C	0.045	0.048	0.009	1.7e-07	ADRB2-SH3TC2
rs1336371	6	140837292	C/T	0.115	-0.031	0.006	1.7e-07	MIR3668-MIR4465
rs192042440	16	69285510	C/T	0.02	0.069	0.012	1.7e-08	SNTB2
rs17210774	3	41527534	C/T	0.172	-0.028	0.005	1.7e-08	ULK4

SNPid	CHR	pos	Effect/other alleles	Effect allele Freq	beta	SE	pval	closest gene
rs55650102	5	122501968	G/A	0.086	0.038	0.007	1.7e-08	PRDM6
rs12799215	11	58405674	C/T	0.238	0.026	0.004	1.7e-09	ZFP91-CNTF-GLYAT
rs6864831	5	127854758	C/T	0.324	-0.025	0.004	1.7e-09	FBN2
rs11228718	11	56440129	C/T	0.129	0.036	0.006	1.7e-10	OR5AR1-OR9G1
rs13330501	16	3551799	A/C	0.185	-0.033	0.005	1.7e-11	CLUAP1
rs8101970	19	1835625	C/T	0.212	0.032	0.005	1.7e-11	REXO1
rs3897821	1	243420388	A/G	0.335	-0.027	0.004	1.7e-11	CEP170,SDCCAG8
rs79094191	21	44720890	T/C	0.037	-0.071	0.01	1.7e-12	CRYAA-LINC00322
rs7539013	1	230849168	C/T	0.37	-0.03	0.004	1.7e-14	AGT
rs73306876	20	57739829	A/G	0.117	-0.084	0.006	1.7e-45	SLMO2-ATP5E-ZNF831
rs4948281	10	63248358	G/A	0.151	-0.022	0.005	1.8e-05	TMEM26-AS1
rs72805692	10	71099109	A/G	0.11	0.025	0.006	1.8e-05	HK1
rs67549624	10	8215279	C/A	0.304	0.018	0.004	1.8e-05	GATA3-LINC00708
rs12225345	11	65647105	A/G	0.165	-0.021	0.005	1.8e-05	CTSW,FIBP,EFEMP2-CTSW
rs10146240	14	35996987	G/A	0.12	0.025	0.006	1.8e-05	NFKBIA-INSM2
rs4777601	15	65100154	G/T	0.126	0.027	0.006	1.8e-05	RBPMS2-PIF1
rs16976295	15	96710126	C/T	0.19	-0.021	0.005	1.8e-05	NR2F2-AS1
rs7499519	16	86167132	A/G	0.065	0.036	0.008	1.8e-05	IRF8-LINC01082
rs12943024	17	7617209	C/T	0.099	0.027	0.006	1.8e-05	DNAH2,EFNB3,EFNB3-DNAH2
rs16827018	1	145545225	T/G	0.052	-0.036	0.008	1.8e-05	ANKRD35,ITGA10,NBPF20,NBPF19,NBPF10
rs209572	1	40836794	G/A	0.382	-0.016	0.004	1.8e-05	SMAP2,COL9A2-SMAP2
rs3810477	20	62191321	A/G	0.443	0.017	0.004	1.8e-05	HELZ2,FNDC11
rs9608690	22	28921347	G/A	0.066	0.032	0.007	1.8e-05	TTC28
rs28573806	22	50727792	T/C	0.392	0.016	0.004	1.8e-05	PLXNB2
rs1992761	2	113810286	G/A	0.079	0.029	0.007	1.8e-05	IL36B
rs13024316	2	142486253	G/A	0.197	-0.02	0.005	1.8e-05	LRP1B
rs62156320	2	98984479	C/T	0.082	0.029	0.007	1.8e-05	CNGA3
rs116455227	5	113788744	C/A	0.017	-0.062	0.014	1.8e-05	KCNN2,LOC101927078
rs42265	5	128404226	G/A	0.445	-0.016	0.004	1.8e-05	SLC27A6-ISOC1
rs6580209	5	141344123	T/C	0.174	0.022	0.005	1.8e-05	RNF14,PCDH12-RNF14
rs1322815	6	109704062	A/G	0.222	-0.019	0.005	1.8e-05	CD164,CD164-PPIL6
rs9365514	6	163362358	C/A	0.443	-0.016	0.004	1.8e-05	PACRG
rs38847	7	116325326	T/C	0.038	0.041	0.01	1.8e-05	MET
rs11978045	7	78140222	A/G	0.422	-0.016	0.004	1.8e-05	MAGI2
rs10114121	9	19440136	G/A	0.15	-0.023	0.005	1.8e-05	ACER2
rs148306899	11	100551578	G/A	0.034	0.049	0.01	1.8e-06	LOC100128386,CNTN5-LOC100128386
rs2509116	11	102948779	G/T	0.272	0.02	0.004	1.8e-06	DCUN1D5
rs1872635	12	54541750	A/G	0.304	0.02	0.004	1.8e-06	LINC02381-SMUG1
rs4984802	16	1340465	A/C	0.468	0.017	0.004	1.8e-06	TPSD1-UBE2I
rs62092642	18	20189121	T/G	0.027	0.054	0.011	1.8e-06	CTAGE1-LOC101927571

SNPid	CHR	pos	Effect/other alleles	Effect allele Freq	beta	SE	pval	closest gene
rs55766730	2	20810024	C/A	0.06	0.038	0.008	1.8e-06	HS1BP3-IT1-HS1BP3
rs12799296	11	22379228	G/A	0.092	-0.033	0.006	1.8e-07	SLC17A6
rs11666888	19	18850707	G/T	0.192	0.025	0.005	1.8e-07	CRTC1
rs2950790	2	145759721	G/A	0.244	0.024	0.004	1.8e-07	TEX41
rs263017	3	183503017	A/G	0.493	0.019	0.004	1.8e-07	YEATS2
rs112689589	12	48190269	C/T	0.22	0.025	0.005	1.8e-08	HDAC7
rs193084249	1	26987646	A/G	0.021	-0.074	0.013	1.8e-08	RPS6KA1-LOC101928728
rs80226362	10	134413500	G/T	0.18	-0.03	0.005	1.8e-09	INPP5A
rs13103931	4	17892400	T/G	0.135	-0.033	0.006	1.8e-09	LCORL
rs10951169	7	27555914	A/G	0.296	0.025	0.004	1.8e-09	EVX1-HIBADH
rs146466236	17	46735946	T/G	0.032	-0.068	0.01	1.8e-10	MIR196A1-PRAC1
rs61772592	1	56979681	A/G	0.119	-0.036	0.006	1.8e-10	PLPP3
rs3823483	7	131010943	T/C	0.46	-0.024	0.004	1.8e-10	MKLN1,MKLN1-AS,MKLN1
rs12423041	12	112324846	T/C	0.173	0.041	0.005	1.8e-16	MAPKAPK5
rs10059884	5	32832474	C/A	0.404	-0.058	0.004	1.8e-51	NPR3-LINC02120
rs11598274	10	130904542	G/T	0.201	-0.02	0.005	1.9e-05	LINC02667-MGMT
rs112948053	10	44958059	C/T	0.033	-0.044	0.01	1.9e-05	CXCL12-TMEM72-AS1
rs1456285	10	63369632	G/A	0.311	0.017	0.004	1.9e-05	TMEM26-AS1-CABCOCO1
rs7943101	11	32460873	C/T	0.174	-0.021	0.005	1.9e-05	WT1,WT1-AS
rs377470	12	50339290	T/G	0.233	-0.019	0.004	1.9e-05	LINC02395-AQP2
rs9532983	13	42634372	A/G	0.02	0.056	0.013	1.9e-05	DGKH
rs6573918	14	70421831	T/C	0.271	-0.018	0.004	1.9e-05	SMOC1
rs7201473	16	86447579	G/A	0.245	-0.02	0.004	1.9e-05	LINC00917-FENDRR
rs142820596	19	16785141	G/A	0.026	0.047	0.012	1.9e-05	TMEM38A
rs33955687	1	107942560	C/A	0.279	0.019	0.004	1.9e-05	NTNG1
rs1539624	1	113431786	G/A	0.448	-0.016	0.004	1.9e-05	MIR11399-SLC16A1
rs74362691	1	46361153	G/A	0.023	-0.052	0.012	1.9e-05	MAST2
rs1451077	2	147901207	G/A	0.422	0.016	0.004	1.9e-05	PABPC1P2-ACVR2A
rs11689842	2	43158542	G/A	0.292	0.017	0.004	1.9e-05	HAAO-LINC01819
rs2698189	2	61268292	T/C	0.099	0.026	0.006	1.9e-05	PEX13
rs279415	5	167127619	C/T	0.211	0.019	0.005	1.9e-05	TENM2
rs143709822	6	127553305	C/T	0.028	0.046	0.011	1.9e-05	RSPO3-RNF146
rs9355515	6	165318519	G/A	0.214	-0.019	0.005	1.9e-05	MEAT6-C6orf118
rs62468466	7	90456493	A/G	0.31	-0.018	0.004	1.9e-05	CDK14
rs10983318	9	119482585	C/T	0.297	0.018	0.004	1.9e-05	ASTN2
rs7918084	10	94429467	C/T	0.443	-0.018	0.004	1.9e-06	KIF11-HHEX
rs9679519	2	211669719	C/A	0.23	-0.02	0.004	1.9e-06	CPS1-ERBB4
rs6731302	2	58833493	A/G	0.432	0.018	0.004	1.9e-06	LINC01122
rs2421649	3	169197333	A/G	0.495	0.017	0.004	1.9e-06	MECOM-AS1,MECOM
rs34322	12	12879570	T/C	0.479	-0.021	0.004	1.9e-08	CDKN1B,APOLD1
rs7133185	12	115932988	G/A	0.38	-0.023	0.004	1.9e-09	TBX3-MED13L

SNPid	CHR	pos	Effect/other alleles	Effect allele Freq	beta	SE	pval	closest gene
rs2960578	18	21143739	T/G	0.499	0.023	0.004	1.9e-09	NPC1
rs2493292	1	3328659	C/T	0.139	-0.032	0.005	1.9e-09	PRDM16
rs11605518	11	13270214	A/G	0.276	-0.028	0.004	1.9e-11	RASSF10-ARNTL
rs62039768	16	51560761	C/A	0.091	-0.044	0.006	1.9e-11	SALL1-LINC01571
rs78230363	19	7504017	C/T	0.229	0.029	0.004	1.9e-11	ARHGEF18
rs1799998	8	143999600	A/G	0.445	0.026	0.004	1.9e-12	CYP11B2,CYP11B2-LY6E-DT
rs76038906	9	113250200	G/T	0.033	-0.081	0.01	1.9e-15	SVEP1
rs6564889	16	81591496	T/C	0.414	-0.032	0.004	1.9e-16	CMIP
rs11838335	12	121891698	G/A	0.392	0.033	0.004	1.9e-17	KDM2B
rs10893870	11	128300953	T/C	0.382	-0.016	0.004	2.1e-05	LINC02098-ETS1
rs12286721	11	47701528	C/A	0.45	0.016	0.004	2.1e-05	AGBL2
rs1950500	14	24830850	T/C	0.294	0.018	0.004	2.1e-05	RIPK3-NFATC4
rs11625845	14	29264552	G/A	0.25	0.019	0.004	2.1e-05	LINC01551,LINC01551-PRKD1
rs55905084	14	36299256	G/A	0.118	0.025	0.006	2.1e-05	BRMS1L
rs11630901	15	41819367	T/C	0.181	0.02	0.005	2.1e-05	RPAP1
rs13380109	15	79378775	G/A	0.416	0.016	0.004	2.1e-05	RASGRF1
rs4786552	16	4860552	G/A	0.089	-0.027	0.006	2.1e-05	GLYR1
rs7503807	17	78591111	A/C	0.44	0.015	0.004	2.1e-05	RPTOR
rs11872991	18	9623036	C/T	0.449	-0.016	0.004	2.1e-05	PPP4R1-AS1,PPP4R1-AS1-RAB31
rs1547725	1	165670958	T/C	0.109	-0.024	0.006	2.1e-05	ALDH9A1,LOC440700
rs12127894	1	42889912	T/C	0.439	-0.016	0.004	2.1e-05	RIMKLA,RIMKLA-ZMYND12
rs1931072	1	97693858	G/A	0.292	-0.017	0.004	2.1e-05	DPYD,DPYD-AS1
rs2223538	20	8583414	T/G	0.203	-0.019	0.005	2.1e-05	PLCB1
rs72856885	2	132152479	A/G	0.183	-0.021	0.005	2.1e-05	RAB6D-LINC01120
rs1900844	2	138275534	C/A	0.322	-0.017	0.004	2.1e-05	THSD7B
rs2968796	2	55287835	T/C	0.474	-0.016	0.004	2.1e-05	RTN4
rs34085137	3	27588131	G/A	0.038	-0.045	0.01	2.1e-05	SLC4A7-EOMES
rs1379932	4	89800892	C/T	0.217	0.018	0.005	2.1e-05	FAM13A
rs10941611	5	43162608	T/C	0.326	-0.017	0.004	2.1e-05	ZNF131
rs28429298	7	109351958	A/G	0.136	-0.024	0.006	2.1e-05	C7orf66-EIF3IP1
rs4723006	7	30764820	T/C	0.43	-0.016	0.004	2.1e-05	CRHR2-INMT
rs72630462	15	85250117	G/A	0.153	0.025	0.005	2.1e-06	SEC11A
rs7203746	16	4256063	A/G	0.277	0.021	0.004	2.1e-06	SRL
rs17272756	19	49642264	A/G	0.139	0.026	0.005	2.1e-06	PPFIA3
rs11919550	3	27605170	A/G	0.276	-0.02	0.004	2.1e-06	SLC4A7-EOMES
rs1915925	3	41125726	G/A	0.111	0.028	0.006	2.1e-06	ZNF621-CTNNB1
rs7756377	6	27481661	G/A	0.285	0.024	0.005	2.1e-06	ZNF184-LINC01012
rs1182450	7	157054079	G/A	0.244	-0.021	0.004	2.1e-06	UBE3C
rs4754705	11	100734650	G/A	0.263	0.023	0.004	2.1e-07	ARHGAP42
rs79716751	13	73821910	G/A	0.044	-0.048	0.009	2.1e-07	KLF5-LINC00392

SNPid	CHR	pos	Effect/other alleles	Effect allele Freq	beta	SE	pval	closest gene
rs1440371	15	66941084	G/A	0.288	-0.021	0.004	2.1e-07	LINC01169
rs34973300	16	2035253	T/C	0.085	-0.035	0.007	2.1e-07	NOXO1,SYNGR3,TBL3,GFER
rs55986668	6	143845589	G/A	0.044	-0.046	0.009	2.1e-07	FUCA2-PHACTR2-AS1
rs2489207	10	18505787	A/G	0.156	0.03	0.005	2.1e-08	CACNB2
rs11008626	10	32037230	T/C	0.295	-0.023	0.004	2.1e-08	ZEB1-ARHGAP12
rs10841520	12	20586395	C/T	0.192	0.027	0.005	2.1e-08	PDE3A
rs115302179	20	11045176	G/T	0.012	-0.095	0.018	2.1e-08	C20orf187-LOC339593
rs6983948	8	95988209	C/T	0.472	0.023	0.004	2.1e-09	NDUFAF6
rs10084596	21	44744540	C/T	0.263	-0.03	0.004	2.1e-11	LINC00322
rs27687	5	68032422	T/C	0.266	-0.029	0.004	2.1e-11	PIK3R1-LINC02198
rs61927826	12	50972580	C/T	0.037	0.068	0.01	2.1e-12	DIP2B
rs12257959	10	115936832	G/T	0.242	-0.032	0.004	2.1e-13	CCDC186,MIR2110,TDRD1,CCDC186-TDRD1
rs532436	9	136149830	G/A	0.182	0.035	0.005	2.1e-13	ABO
rs12415349	10	105525454	G/A	0.178	-0.022	0.005	2.2e-05	SH3PXD2A
rs2650000	12	121388962	A/C	0.337	0.018	0.004	2.2e-05	XLOC_009911-HNF1A-AS1
rs11106296	12	92211755	A/G	0.12	0.023	0.006	2.2e-05	DCN-LINC01619
rs11622651	14	104620077	G/A	0.292	0.018	0.004	2.2e-05	KIF26A
rs142673229	17	46178773	G/T	0.026	0.048	0.012	2.2e-05	CBX1
rs1531554	17	79380547	T/C	0.453	-0.017	0.004	2.2e-05	BAHCC1
rs2972574	19	8394092	T/C	0.437	0.016	0.004	2.2e-05	KANK3
rs494173	1	116000324	A/C	0.044	-0.039	0.009	2.2e-05	NGF-AS1-VANGL1
rs3766696	1	175142462	A/G	0.089	-0.029	0.007	2.2e-05	KIAA0040
rs6067800	20	36464696	C/T	0.359	-0.017	0.004	2.2e-05	CTNNBL1
rs1016593	5	122632603	C/A	0.325	0.017	0.004	2.2e-05	PRDM6-CEP120
rs62385431	5	158475691	A/G	0.016	0.06	0.014	2.2e-05	EBF1
rs26649	5	61699451	C/T	0.33	0.017	0.004	2.2e-05	DIMT1
rs4947013	6	110021317	T/C	0.238	0.019	0.004	2.2e-05	FIG4
rs147561921	6	79407932	T/G	0.138	0.023	0.005	2.2e-05	MIR10524-IRAK1BP1
rs1229762	7	114218582	C/T	0.327	-0.016	0.004	2.2e-05	FOXP2
rs80014594	7	114691804	G/A	0.112	0.024	0.006	2.2e-05	MDFIC-LINC01393
rs4729578	7	99742093	G/A	0.216	0.019	0.005	2.2e-05	LAMTOR4,MBLAC1-LAMTOR4
rs7823561	8	25641764	A/C	0.338	0.017	0.004	2.2e-05	CDCA2-EBF2
rs62508370	8	30417026	G/A	0.257	-0.018	0.004	2.2e-05	RBPMS
rs2480927	9	21366894	C/T	0.205	-0.019	0.005	2.2e-05	IFNA13,IFNA6-IFNA13
rs77339850	9	71828034	C/T	0.056	-0.035	0.008	2.2e-05	TJP2
rs11191851	10	105652965	C/T	0.044	-0.044	0.009	2.2e-06	STN1
rs6480716	10	75875794	A/G	0.395	0.019	0.004	2.2e-06	AP3M1,VCL
rs2774940	1	93311761	T/G	0.097	0.029	0.006	2.2e-06	RPL5,DIPK1A
rs2692938	2	96753149	G/A	0.211	-0.022	0.005	2.2e-06	GPAT2-ADRA2B
rs78009918	4	102195416	G/A	0.025	0.055	0.011	2.2e-06	PPP3CA

SNPid	CHR	pos	Effect/other alleles	Effect allele Freq	beta	SE	pval	closest gene
rs17008782	4	120930543	C/T	0.355	-0.018	0.004	2.2e-06	LINC02502-MAD2L1
rs7130440	11	111417779	A/G	0.363	-0.02	0.004	2.2e-07	LAYN
rs75899721	12	115512249	A/G	0.119	-0.031	0.006	2.2e-07	TBX3-MED13L
rs7970687	12	133730500	C/T	0.448	-0.021	0.004	2.2e-07	ZNF10
rs17317669	6	25406031	T/C	0.272	0.022	0.004	2.2e-07	CARMIL1
rs555007	6	31850332	T/C	0.065	0.045	0.008	2.2e-07	SLC44A4,EHMT2
rs12324086	15	61475188	C/T	0.366	0.022	0.004	2.2e-08	RORA
rs2396141	19	8492574	G/A	0.285	0.024	0.004	2.2e-08	MARCHF2
rs755249	1	39995074	C/T	0.225	-0.025	0.004	2.2e-08	BMP8A,PPIEL
rs762395	21	44769676	A/G	0.339	-0.024	0.004	2.2e-08	LINC00322-LINC01679
rs7759741	6	27241043	C/T	0.206	-0.034	0.006	2.2e-08	PRSS16-POM121L2
rs17679729	14	103924312	A/G	0.359	-0.023	0.004	2.2e-09	MARK3
rs12090626	1	193123221	T/C	0.311	0.024	0.004	2.2e-09	CDC73
rs61940962	12	48595477	A/C	0.211	-0.029	0.005	2.2e-10	OR10AD1,CCDC184-OR10AD1
rs9872754	3	138117985	C/T	0.156	-0.034	0.005	2.2e-11	MRAS
rs2341599	4	156433308	G/A	0.345	0.031	0.004	2.2e-14	MAP9-GUCY1A1
rs1505194	11	4706037	C/A	0.344	0.016	0.004	2.3e-05	OR51E2
rs649392	11	69464793	G/A	0.452	0.016	0.004	2.3e-05	CCND1
rs77100509	12	110560318	C/T	0.034	0.042	0.011	2.3e-05	IFT81,C12orf76-IFT81
rs117581081	14	58826493	T/C	0.033	-0.043	0.01	2.3e-05	ARID4A
rs40834	16	28510393	C/T	0.456	-0.016	0.004	2.3e-05	APOBR,IL27,APOBR-IL27
rs2072339	16	4039716	A/C	0.202	0.02	0.005	2.3e-05	ADCY9
rs6693583	1	111404055	T/G	0.324	-0.019	0.004	2.3e-05	KCNA3-CD53
rs6136437	20	18596424	T/C	0.107	0.025	0.006	2.3e-05	DTD1
rs9677933	2	121337280	C/T	0.156	0.021	0.005	2.3e-05	LINC01101-GLI2
rs13420652	2	121855879	C/T	0.242	-0.018	0.004	2.3e-05	GLI2-TFCP2L1
rs4550865	4	157013065	C/A	0.399	0.015	0.004	2.3e-05	CTSO-PDGFC
rs115271554	5	113546951	T/C	0.057	-0.035	0.008	2.3e-05	KCNN2
rs6883481	5	166132929	C/T	0.479	-0.016	0.004	2.3e-05	LOC102546299-LINC01947
rs2817420	6	50813332	T/C	0.156	-0.021	0.005	2.3e-05	TFAP2B
rs310583	7	150901609	A/G	0.489	0.016	0.004	2.3e-05	IQCA1L,H2BE1,LOC114483834
rs10138425	14	100604334	A/G	0.155	-0.024	0.005	2.3e-06	EVL
rs34050628	15	91398600	C/T	0.119	0.028	0.006	2.3e-06	BLM-FURIN
rs8070792	17	79365166	A/G	0.056	0.039	0.008	2.3e-06	LOC100130370-BAHCC1
rs7551303	1	215351386	T/C	0.058	0.038	0.008	2.3e-06	KCNK2
rs7511635	1	5281675	A/C	0.347	0.019	0.004	2.3e-06	AJAP1-MIR4689
rs10194077	2	66771863	T/C	0.39	-0.018	0.004	2.3e-06	MEIS1
rs30631	5	14292644	G/A	0.247	-0.02	0.004	2.3e-06	TRIO
rs3757424	7	150067679	C/A	0.32	0.019	0.004	2.3e-06	REPIN1
rs2607071	8	95255090	G/A	0.126	-0.027	0.006	2.3e-06	CDH17-GEM

SNPid	CHR	pos	Effect/other alleles	Effect allele Freq	beta	SE	pval	closest gene
rs2354356	10	88433895	A/G	0.487	0.02	0.004	2.3e-07	LDB3
rs17108967	14	71542168	T/C	0.338	0.02	0.004	2.3e-07	PCNX1
rs7588910	2	27384579	A/G	0.405	-0.019	0.004	2.3e-07	TCF23,TCF23-SLC5A6
rs113692401	5	127761299	T/C	0.04	-0.049	0.01	2.3e-07	FBN2
rs189487879	6	26980165	A/C	0.044	0.048	0.009	2.3e-07	LINC00240
rs7775795	6	28084828	G/A	0.031	-0.058	0.011	2.3e-07	ZSCAN16-AS1,ZSCAN12P1-ZSCAN16-AS1
rs12958584	18	19632380	C/T	0.132	-0.032	0.006	2.3e-08	MIB1-GATA6-AS1
rs12599865	16	81614679	G/A	0.083	0.04	0.007	2.3e-09	CMIP
rs10922500	1	89359543	A/C	0.378	-0.026	0.004	2.3e-11	GTF2B,GTF2B-KYAT3
rs300934	4	144171748	T/G	0.313	0.029	0.004	2.3e-13	USP38-GAB1
rs2137320	11	1884342	G/A	0.386	-0.043	0.004	2.3e-28	MIR4298,LSP1,LSP1
rs3016173	11	60790839	G/A	0.402	0.016	0.004	2.4e-05	CD6,CD6-CD5
rs12367370	12	69853934	A/G	0.404	-0.016	0.004	2.4e-05	YEATS4-FRS2
rs79693929	13	114623263	C/T	0.138	0.023	0.005	2.4e-05	LINC00452
rs9552466	13	22262231	C/T	0.332	0.016	0.004	2.4e-05	FGF9
rs1934229	1	163603466	T/C	0.445	-0.016	0.004	2.4e-05	LOC100422212-PBX1
rs12030051	1	21134597	G/A	0.438	-0.016	0.004	2.4e-05	EIF4G3
rs3805620	5	127606864	T/C	0.081	-0.029	0.007	2.4e-05	FBN2
rs73279695	8	103109107	T/C	0.113	0.025	0.006	2.4e-05	NCALD
rs17069121	8	3998555	G/A	0.179	0.02	0.005	2.4e-05	CSMD1
rs7907026	10	107406014	G/A	0.491	-0.017	0.004	2.4e-06	SORCS3-LINC02627
rs77403571	11	45913607	G/A	0.057	0.037	0.008	2.4e-06	MAPK8IP1
rs533018	11	64819218	A/G	0.285	-0.02	0.004	2.4e-06	NAALADL1
rs219779	21	37833751	G/A	0.259	0.02	0.004	2.4e-06	CLDN14
rs7565996	2	86113282	T/C	0.309	0.02	0.004	2.4e-06	ST3GAL5-AS1,ST3GAL5
rs62262084	3	47653822	C/T	0.25	0.021	0.004	2.4e-06	SMARCC1
rs4593081	4	156263285	T/C	0.462	-0.017	0.004	2.4e-06	MAP9,NPY2R-MAP9
rs11943964	4	53863203	T/C	0.262	-0.02	0.004	2.4e-06	SCFD2
rs62377186	5	168337362	C/T	0.053	-0.038	0.008	2.4e-06	SLIT3
rs1041926	6	28426296	G/A	0.03	-0.054	0.011	2.4e-06	ZSCAN23-GPX6
rs72902647	18	42746809	C/T	0.171	0.025	0.005	2.4e-07	SETBP1-SLC14A2
rs2613794	19	5143294	G/A	0.334	0.02	0.004	2.4e-07	KDM4B
rs57778433	1	27712608	G/A	0.081	0.034	0.007	2.4e-07	CD164L2,CD164L2-GPR3
rs10255425	7	155754773	T/C	0.445	-0.02	0.004	2.4e-07	LOC389602,LOC389602
rs7894090	10	18291288	G/A	0.145	0.03	0.005	2.4e-08	SLC39A12,SLC39A12-AS1
rs189583044	8	26127884	C/T	0.031	-0.056	0.011	2.4e-08	EBF2-PPP2R2A
rs2026427	10	45389704	T/C	0.28	0.025	0.004	2.4e-09	TMEM72-AS1
rs10789340	1	72940273	A/G	0.372	-0.023	0.004	2.4e-09	NEGR1-LINC01360
rs147405800	5	158412406	G/T	0.028	0.065	0.011	2.4e-09	EBF1
rs62229372	21	37692507	C/T	0.12	-0.036	0.006	2.4e-10	MORC3

SNPid	CHR	pos	Effect/other alleles	Effect allele Freq	beta	SE	pval	closest gene
rs4511593	17	7455536	C/T	0.351	0.027	0.004	2.4e-11	TNFSF12,TNFSF12-TNFSF13
rs11657371	17	46843873	T/C	0.258	0.029	0.004	2.4e-12	TTLL6
rs1522153	3	27170777	G/A	0.3	0.032	0.004	2.4e-14	NEK10
rs12567136	1	11883731	C/T	0.162	0.071	0.005	2.4e-43	CLCN6
rs61872966	10	122956687	G/A	0.257	0.018	0.004	2.5e-05	WDR11-FGFR2
rs1890825	11	30172579	G/A	0.357	0.017	0.004	2.5e-05	KCNA4-FSHB
rs76912408	12	13834291	T/C	0.178	0.021	0.005	2.5e-05	GRIN2B
rs6538386	12	93507311	T/C	0.193	0.019	0.005	2.5e-05	LOC643339
rs9554229	13	28639249	T/C	0.183	-0.021	0.005	2.5e-05	FLT3
rs279076	13	51100659	C/T	0.329	-0.017	0.004	2.5e-05	DLEU1-AS1,DLEU1
rs10438404	15	38938017	G/T	0.402	-0.015	0.004	2.5e-05	RASGRP1-LINC02694
rs139974673	15	44027885	T/C	0.025	-0.052	0.012	2.5e-05	CATSPER2P1,STRCP1-CATSPER2P1
rs10432203	18	48180842	A/C	0.328	0.017	0.004	2.5e-05	MAPK4
rs10402151	19	21446930	C/A	0.48	-0.016	0.004	2.5e-05	ZNF431-ZNF708
rs6040427	20	11185966	T/C	0.335	-0.017	0.004	2.5e-05	C20orf187-LOC339593
rs11907476	20	49047848	G/A	0.312	0.016	0.004	2.5e-05	LINC01271-PTPN1
rs8176498	2	188353919	G/A	0.299	0.017	0.004	2.5e-05	TFPI
rs116242176	2	58543836	G/A	0.026	0.048	0.012	2.5e-05	LINC01795-LINC01122
rs16903982	5	38466747	G/A	0.078	-0.029	0.007	2.5e-05	EGFLAM,EGFLAM-LIFR
rs9387472	6	117714885	C/A	0.085	0.028	0.007	2.5e-05	ROS1
rs116719739	6	27798937	C/T	0.038	0.043	0.01	2.5e-05	H4C12,H4C11-H4C12
rs10484946	6	79856630	T/C	0.09	0.028	0.007	2.5e-05	PHIP-HMGN3
rs6954479	7	103403948	A/C	0.161	-0.021	0.005	2.5e-05	RELN
rs9657620	9	34512552	G/A	0.37	0.016	0.004	2.5e-05	DNAI1
rs84086	10	121378250	T/C	0.445	-0.017	0.004	2.5e-06	TIAL1-BAG3
rs11225866	11	103411740	T/G	0.22	0.021	0.005	2.5e-06	DYNC2H1-MIR4693
rs41288297	13	42404710	C/T	0.047	-0.043	0.009	2.5e-06	VWA8
rs60603802	17	42665402	A/G	0.097	-0.029	0.006	2.5e-06	FZD2-LINC01180
rs2289321	2	111870220	T/C	0.063	-0.036	0.008	2.5e-06	ACOXL,ACOXL-AS1
rs73174368	3	169304310	G/T	0.056	-0.036	0.008	2.5e-06	MECOM
rs10519731	5	122941704	A/G	0.145	0.025	0.005	2.5e-06	CSNK1G3
rs9356839	6	22841522	T/C	0.371	0.019	0.004	2.5e-06	LOC105374972-NRSN1
rs2011695	10	118507219	A/C	0.396	-0.02	0.004	2.5e-07	HSPA12A
rs56170410	10	8240873	C/T	0.206	-0.023	0.005	2.5e-07	GATA3-LINC00708
rs2811477	3	127898783	C/T	0.09	0.033	0.007	2.5e-07	EEFSEC
rs2206738	6	20666246	A/G	0.316	-0.021	0.004	2.5e-07	CDKAL1
rs3894010	18	48167237	G/T	0.407	0.022	0.004	2.5e-08	MAPK4
rs13017832	2	175170792	G/A	0.387	0.022	0.004	2.5e-08	OLA1-LINC01305
rs7694531	4	96087101	A/G	0.408	0.022	0.004	2.5e-08	UNC5C
rs10062049	5	61553881	C/T	0.136	-0.031	0.006	2.5e-08	C5orf64-AS1-KIF2A
rs73050466	7	1141575	G/A	0.027	0.071	0.011	2.5e-10	C7orf50

SNPid	CHR	pos	Effect/other alleles	Effect allele Freq	beta	SE	pval	closest gene
rs4372964	20	62707527	C/T	0.432	0.025	0.004	2.5e-11	OPRL1,TCEA2,MIR6813,RGS19
rs2052926	2	43394781	C/T	0.26	0.028	0.004	2.5e-11	LINC02580-ZFP36L2
rs7515635	1	42408070	T/C	0.461	0.026	0.004	2.5e-12	HIVEP3
rs56329057	4	156633444	C/T	0.181	0.042	0.005	2.5e-18	GUCY1A1
rs743575	10	104594906	T/G	0.284	-0.018	0.004	2.6e-05	CYP17A1
rs79889784	11	1702117	G/T	0.017	0.062	0.015	2.6e-05	FAM99B,FAM99A-FAM99B
rs10769961	11	8829105	G/T	0.214	0.019	0.005	2.6e-05	DENND2B,LOC102724784
rs17465916	12	90009929	G/A	0.04	-0.04	0.009	2.6e-05	ATP2B1
rs4887128	15	74587965	G/A	0.187	-0.019	0.005	2.6e-05	CCDC33
rs12149660	16	70309237	G/A	0.114	0.025	0.006	2.6e-05	AARS1
rs12142313	1	198772260	T/G	0.132	-0.023	0.005	2.6e-05	MIR181A1HG,PTPRC-MIR181A1HG
rs6848761	4	817172	C/T	0.041	0.039	0.009	2.6e-05	CPLX1
rs246600	5	142516897	C/T	0.464	-0.016	0.004	2.6e-05	ARHGAP26
rs73172008	7	156730688	T/C	0.182	-0.02	0.005	2.6e-05	LMBR1-NOM1
rs10958696	8	41571233	T/C	0.35	0.016	0.004	2.6e-05	ANK1
rs2281767	13	99099338	C/T	0.486	-0.018	0.004	2.6e-06	STK24,FARP1
rs1025686	18	48147127	A/G	0.239	0.021	0.004	2.6e-06	MAPK4
rs62169493	2	146876922	C/T	0.38	0.019	0.004	2.6e-06	TEX41-PABPC1P2
rs2145375	3	197063819	C/T	0.266	-0.02	0.004	2.6e-06	DLG1-AS1-LINC02012
rs72851179	6	28103473	G/A	0.047	0.056	0.013	2.6e-06	ZSCAN16-AS1
rs35255141	10	18782777	G/T	0.153	-0.027	0.005	2.6e-07	CACNB2
rs4404633	5	63850157	C/A	0.246	-0.022	0.004	2.6e-07	RGS7BP
rs10108728	8	106238481	A/C	0.399	-0.02	0.004	2.6e-07	LRP12-ZFPM2
rs4653933	1	228445187	A/G	0.384	0.021	0.004	2.6e-08	OBSCN
rs112903399	2	113486880	A/G	0.056	0.043	0.008	2.6e-08	NT5DC4
rs922887	4	156483953	A/G	0.15	0.03	0.005	2.6e-08	MAP9-GUCY1A1
rs204883	6	32032743	G/A	0.383	-0.029	0.005	2.6e-08	TNXB
rs4808569	19	17218970	C/A	0.198	0.031	0.005	2.6e-11	MYO9B
rs77114905	5	148385614	G/A	0.185	0.032	0.005	2.6e-11	SH3TC2
rs143278243	6	26095442	G/A	0.011	-0.121	0.018	2.6e-12	HFE,LOC108783645
rs11040359	11	49437005	G/T	0.146	0.042	0.005	2.6e-15	FOLH1-LOC440040
rs68085814	17	47135875	G/A	0.295	0.033	0.004	2.6e-16	IGF2BP1,IGF2BP1-B4GALNT2
rs76935275	10	124166835	T/G	0.116	-0.025	0.006	2.7e-05	PLEKHA1
rs11029977	11	1494775	T/C	0.386	0.017	0.004	2.7e-05	MOB2
rs28505800	12	110825979	C/A	0.032	0.043	0.011	2.7e-05	ANAPC7
rs11054935	12	12648843	A/G	0.267	0.018	0.004	2.7e-05	DUSP16
rs61614746	12	54740214	G/A	0.115	0.024	0.006	2.7e-05	COPZ1
rs3816291	17	76440652	G/A	0.084	0.028	0.007	2.7e-05	DNAH17
rs62123672	19	2583037	T/C	0.038	-0.041	0.01	2.7e-05	GNG7
rs73159006	22	29923084	A/G	0.228	-0.019	0.004	2.7e-05	THOC5

SNPid	CHR	pos	Effect/other alleles	Effect allele Freq	beta	SE	pval	closest gene
rs9616900	22	51098443	A/G	0.465	-0.017	0.004	2.7e-05	ARSA-SHANK3
rs2880119	2	111809330	C/A	0.137	0.022	0.005	2.7e-05	ACOXL
rs2571445	2	218683154	A/G	0.391	0.016	0.004	2.7e-05	TNS1
rs935919	2	36189668	T/C	0.47	-0.016	0.004	2.7e-05	MIR548AD-CRIM1-DT
rs2708149	2	58951187	A/G	0.427	-0.016	0.004	2.7e-05	LINC01122
rs6965571	7	150434472	G/A	0.166	-0.022	0.005	2.7e-05	GIMAP5,GIMAP1-GIMAP5
rs6994937	8	141117789	G/A	0.368	0.017	0.004	2.7e-05	TRAPPC9
rs55892969	11	8119298	A/G	0.036	-0.047	0.01	2.7e-06	TUB,RIC3
rs2928064	12	58085198	T/C	0.333	0.019	0.004	2.7e-06	OS9,B4GALNT1-OS9
rs72839768	17	7129898	G/A	0.023	0.06	0.012	2.7e-06	DVL2,MIR324,ACADVL
rs116123382	1	115293353	C/T	0.021	-0.059	0.013	2.7e-06	CSDE1
rs9425633	1	184657251	T/C	0.488	-0.018	0.004	2.7e-06	EDEM3,C1orf21-EDEM3
rs12097349	1	29576015	G/A	0.064	-0.035	0.008	2.7e-06	PTPRU
rs2171618	4	56430408	A/G	0.308	0.019	0.004	2.7e-06	PDCL2
rs6874493	5	76701739	A/G	0.466	0.018	0.004	2.7e-06	PDE8B
rs67687820	9	35922816	T/C	0.136	0.025	0.005	2.7e-06	SPAAR-OR2S2
rs144953311	12	51606328	G/A	0.075	0.036	0.007	2.7e-07	POU6F1
rs10413734	19	7227871	T/G	0.328	0.021	0.004	2.7e-07	INSR
rs2358668	2	175253824	A/C	0.241	0.023	0.004	2.7e-07	CIR1
rs2070731	5	131819798	T/C	0.333	-0.02	0.004	2.7e-07	IRF1
rs4712930	6	25403707	A/G	0.152	-0.028	0.005	2.7e-07	CARMIL1
rs9467570	6	25699283	C/A	0.286	0.023	0.004	2.7e-07	SCGN
rs1515110	2	227122216	G/T	0.361	-0.02	0.004	2.7e-08	LOC646736-MIR5702
rs1055447	11	47186424	C/A	0.46	-0.023	0.004	2.7e-09	ARFGAP2,C11orf49
rs9309103	2	43863866	G/A	0.37	0.024	0.004	2.7e-10	PLEKHH2,THADA-PLEKHH2
rs59400568	9	112757346	A/G	0.196	0.031	0.005	2.7e-10	PALM2AKAP2
rs36077257	4	81179990	C/T	0.024	-0.083	0.012	2.7e-12	PRDM8-FGF5
rs4722681	7	27320319	T/C	0.1	-0.049	0.006	2.7e-15	EVX1-HIBADH
rs13107325	4	103188709	C/T	0.072	0.064	0.007	2.7e-18	SLC39A8
rs906934	10	64759410	G/A	0.289	0.018	0.004	2.8e-05	EGR2-NRBF2
rs7077767	10	76880322	C/T	0.053	-0.036	0.009	2.8e-05	SAMD8
rs4938313	11	116671005	A/G	0.112	0.024	0.006	2.8e-05	APOA5-APOA4
rs72931727	11	69815306	C/T	0.047	0.038	0.009	2.8e-05	FGF3-LINC02753
rs7939397	11	9777421	C/T	0.019	-0.06	0.014	2.8e-05	SBF2-AS1,SWAP70,LINC02709
rs3935332	12	122036525	A/G	0.065	0.031	0.007	2.8e-05	MIR548AQ,MIR548AQ-ORAI1
rs114321782	15	63813341	A/C	0.243	-0.018	0.004	2.8e-05	USP3
rs16941174	15	88592449	G/A	0.089	0.029	0.007	2.8e-05	NTRK3
rs3897644	18	57729069	C/T	0.453	0.016	0.004	2.8e-05	PMAIP1-MC4R
rs9442373	1	1062638	C/A	0.44	-0.016	0.004	2.8e-05	C1orf159-LINC01342
rs79060661	7	2570655	A/C	0.037	-0.042	0.01	2.8e-05	LFNG,MIR4648,LFNG-

SNPid	CHR	pos	Effect/other alleles	Effect allele Freq	beta	SE	pval	closest gene
								BRAT1
rs2158134	7	92454202	T/C	0.134	0.022	0.006	2.8e-05	CDK6
rs2046834	11	46218910	A/G	0.353	0.018	0.004	2.8e-06	LOC101928894-CREB3L1
rs138337894	11	50346573	C/T	0.076	-0.033	0.007	2.8e-06	LOC441601-LOC646813
rs35732828	17	44833217	C/A	0.155	-0.024	0.005	2.8e-06	NSF
rs111437402	18	48403285	G/A	0.046	-0.043	0.009	2.8e-06	ME2,MRO-ME2
rs11881242	19	36283847	T/C	0.098	-0.03	0.006	2.8e-06	ARHGAP33,LINC01529
rs4847327	1	93045786	A/G	0.097	0.028	0.006	2.8e-06	EVI5
rs242609	20	30673424	C/T	0.265	0.02	0.004	2.8e-06	HCK
rs12622267	2	181568007	A/G	0.359	0.018	0.004	2.8e-06	SCHLAP1
rs74509660	9	100512962	G/A	0.061	0.037	0.008	2.8e-06	PTCSC2
rs78016875	3	54069872	A/G	0.019	0.072	0.014	2.8e-07	SELENOK-CACNA2D3
rs61910264	11	122608704	C/T	0.454	-0.02	0.004	2.8e-08	UBASH3B
rs3803802	17	7621464	C/A	0.454	0.021	0.004	2.8e-08	DNAH2
rs17429037	7	27386701	G/A	0.067	-0.041	0.007	2.8e-08	EVX1-HIBADH
rs7616723	3	150055644	G/A	0.093	-0.039	0.006	2.8e-09	LINC01214-TSC22D2
rs42038	7	92243719	C/T	0.295	0.024	0.004	2.8e-09	CDK6
rs4845875	1	11824133	A/C	0.357	-0.024	0.004	2.8e-10	C1orf167
rs11169294	12	50561998	A/G	0.368	-0.033	0.004	2.8e-17	CERS5,CERS5-LIMA1
rs4881719	11	49758987	A/G	0.095	0.028	0.007	2.9e-05	LOC440040
rs56679184	16	5789665	G/A	0.129	0.024	0.006	2.9e-05	MIR8065-RBFOX1
rs12149877	16	71996067	T/C	0.252	-0.018	0.004	2.9e-05	PKD1L3
rs2966858	16	85321483	T/C	0.197	0.02	0.005	2.9e-05	LINC00311
rs4791603	17	14720368	T/G	0.312	-0.016	0.004	2.9e-05	HS3ST3B1-LOC101928475
rs757411	17	38775150	C/T	0.363	0.017	0.004	2.9e-05	CCR7-SMARCE1
rs7256689	19	17273893	G/T	0.341	0.017	0.004	2.9e-05	MYO9B
rs5752866	22	29498118	G/A	0.104	-0.025	0.006	2.9e-05	KREMEN1
rs11678837	2	107488998	C/T	0.3	-0.016	0.004	2.9e-05	ST6GAL2
rs118077746	6	26905300	T/C	0.087	0.037	0.009	2.9e-05	GUSBP2
rs7213756	17	2098339	C/A	0.329	-0.019	0.004	2.9e-06	SMG6
rs6035372	20	19516254	G/A	0.224	0.021	0.005	2.9e-06	SLC24A3
rs7567791	2	235779747	T/C	0.101	0.028	0.006	2.9e-06	LINC01173-SH3BP4
rs3856803	3	11673402	C/T	0.497	-0.018	0.004	2.9e-06	VGLL4
rs7007207	8	82618801	T/C	0.127	-0.025	0.006	2.9e-06	ZFAND1
rs10261050	7	114337652	C/T	0.469	-0.019	0.004	2.9e-07	FOXP2,FOXP2-MDFIC
rs61911503	11	122524741	G/A	0.117	-0.031	0.006	2.9e-08	UBASH3B,MIR100HG-UBASH3B
rs6108676	20	10688744	C/A	0.369	0.022	0.004	2.9e-08	JAG1-LINC01752
rs78643014	5	68403982	T/C	0.024	-0.065	0.012	2.9e-08	SLC30A5
rs112563428	6	34800435	C/T	0.113	-0.032	0.006	2.9e-08	UHRF1BP1
rs28485388	3	169311841	A/G	0.303	-0.023	0.004	2.9e-09	MECOM
rs2417849	12	20276513	C/T	0.341	-0.025	0.004	2.9e-10	LINC02468

SNPid	CHR	pos	Effect/other alleles	Effect allele Freq	beta	SE	pval	closest gene
rs141947880	11	47385350	C/T	0.053	-0.056	0.008	2.9e-11	SPI1
rs13112145	4	138413244	T/C	0.419	-0.028	0.004	2.9e-13	LINC02511-PCDH18
rs448385	1	25395133	G/A	0.441	-0.031	0.004	2.9e-16	MIR4425-SYF2
rs7120737	11	47702395	A/G	0.147	0.051	0.005	2.9e-21	AGBL2
rs1957563	5	157474590	C/T	0.266	-0.04	0.004	2.9e-21	CLINT1-LINC02227
rs12819192	12	50539589	C/T	0.035	-0.042	0.01	3.1e-05	CERS5
rs2029938	12	62855364	A/G	0.362	0.016	0.004	3.1e-05	USP15-MON2
rs77083992	12	70326498	A/G	0.133	-0.022	0.005	3.1e-05	MYRFL
rs141020647	15	42440297	G/A	0.02	-0.054	0.013	3.1e-05	PLA2G4F
rs117818985	16	4974305	C/A	0.015	0.06	0.015	3.1e-05	PPL
rs12938771	17	47112408	C/T	0.169	-0.02	0.005	3.1e-05	IGF2BP1
rs117185237	17	51345853	C/T	0.03	-0.044	0.011	3.1e-05	C17orf112-KIF2B
rs7551188	1	25273200	T/C	0.484	-0.016	0.004	3.1e-05	RUNX3
rs11247797	1	28716638	A/G	0.307	0.016	0.004	3.1e-05	PHACTR4
rs11207709	1	61655241	G/A	0.11	0.025	0.006	3.1e-05	NFIA
rs62168888	2	163751533	G/A	0.062	0.032	0.008	3.1e-05	KCNH7-FIGN
rs2857539	2	177024863	G/A	0.309	-0.017	0.004	3.1e-05	HOXD3
rs9815587	3	141674548	C/A	0.146	-0.022	0.005	3.1e-05	TFDP2
rs2164554	3	143755528	A/G	0.322	-0.016	0.004	3.1e-05	DIPK2A-LNCSRLR
rs1604685	3	144016694	C/T	0.206	-0.019	0.005	3.1e-05	DIPK2A-LNCSRLR
rs11931102	4	2222471	T/C	0.045	-0.038	0.009	3.1e-05	POLN
rs78156284	4	52853439	T/C	0.087	-0.028	0.007	3.1e-05	DCUN1D4-LRRC66
rs7775523	6	20955332	T/C	0.333	0.017	0.004	3.1e-05	CDKAL1
rs17432448	7	18548419	A/G	0.369	-0.016	0.004	3.1e-05	HDAC9
rs1521780	8	106008412	G/A	0.068	0.03	0.007	3.1e-05	LRP12-ZFPM2
rs13258335	8	1802711	G/A	0.216	-0.019	0.005	3.1e-05	ARHGEF10
rs35133675	10	134312857	C/T	0.44	0.017	0.004	3.1e-06	C10orf91-LOC107984282
rs28399271	15	67710011	G/A	0.262	0.019	0.004	3.1e-06	IQCH,IQCH-AS1
rs7188071	16	28917644	T/C	0.346	0.018	0.004	3.1e-06	ATP2A1,RABEP2
rs16970025	17	47243622	G/T	0.29	0.02	0.004	3.1e-06	B4GALNT2
rs4511641	19	45993568	A/C	0.444	0.018	0.004	3.1e-06	RTN2
rs13447553	1	91988919	T/G	0.157	0.024	0.005	3.1e-06	CDC7
rs73166358	3	157977793	C/T	0.29	-0.019	0.004	3.1e-06	RSRC1
rs4142419	4	106912250	C/A	0.095	0.03	0.007	3.1e-06	NPNT-LOC101929577
rs12535208	7	150312857	G/A	0.219	0.021	0.005	3.1e-06	GIMAP4-GIMAP6
rs12767891	10	115748333	T/G	0.496	-0.02	0.004	3.1e-07	NHLRC2-ADRB1
rs72681624	14	51135609	G/A	0.038	0.048	0.01	3.1e-07	SAV1,SAV1-NIN
rs62083434	17	57019660	T/C	0.204	-0.023	0.005	3.1e-07	PPM1E
rs9960575	18	54702309	T/C	0.28	-0.021	0.004	3.1e-07	WDR7-LINC-ROR
rs7902062	10	60586473	C/T	0.271	-0.024	0.004	3.1e-08	BICC1
rs1741344	20	4101800	C/T	0.363	-0.022	0.004	3.1e-08	RNF24-SMOX

SNPid	CHR	pos	Effect/other alleles	Effect allele Freq	beta	SE	pval	closest gene
rs11196553	10	115710997	C/T	0.045	-0.064	0.009	3.1e-12	NHLRC2-ADRB1
rs6726740	2	165050128	T/G	0.272	0.033	0.004	3.1e-15	FIGN-GRB14
rs7092571	10	27065536	G/A	0.178	-0.019	0.005	3.2e-05	ABI1
rs73387428	12	105921854	T/G	0.085	0.029	0.007	3.2e-05	C12orf75-CASC18
rs4280357	18	19882403	A/G	0.348	-0.017	0.004	3.2e-05	GATA6-CTAGE1
rs116482300	1	46150680	T/C	0.039	0.038	0.01	3.2e-05	TMEM69,GPBP1L1
rs7590726	2	167247291	T/G	0.388	0.016	0.004	3.2e-05	SCN9A-SCN7A
rs28368942	4	109052225	G/A	0.076	-0.03	0.007	3.2e-05	LEF1
rs258952	5	92055195	C/T	0.337	-0.017	0.004	3.2e-05	ARRDC3-AS1-NR2F1-AS1
rs997694	9	16484870	T/C	0.336	-0.017	0.004	3.2e-05	BNC2
rs11228613	11	69068492	T/G	0.232	0.022	0.005	3.2e-06	MYEOV,MYEOV-LOC102724265
rs79037865	16	71437135	C/T	0.046	0.042	0.009	3.2e-06	CALB2-LINC02136
rs1933629	1	231075574	C/T	0.131	-0.027	0.006	3.2e-06	TTC13
rs1515132	2	117558695	T/C	0.356	0.017	0.004	3.2e-06	DPP10-DDX18
rs4864422	4	138374616	A/G	0.451	0.018	0.004	3.2e-06	LINC02511-PCDH18
rs7711823	5	158489315	A/G	0.361	0.019	0.004	3.2e-06	EBF1
rs7714850	5	63036337	C/A	0.368	-0.018	0.004	3.2e-06	IPO11-HTR1A
rs116268808	6	28756722	C/T	0.042	0.057	0.013	3.2e-06	LINC00533-LINC01623
rs2814985	6	34548296	C/T	0.131	-0.025	0.006	3.2e-06	SPDEF-ILRUN
rs61955585	12	122660392	T/G	0.146	-0.027	0.005	3.2e-07	IL31,LRRC43
rs10822184	10	65337153	T/C	0.493	0.023	0.004	3.2e-09	REEP3
rs10064027	5	158483282	G/A	0.145	0.031	0.005	3.2e-09	EBF1
rs7306947	12	53385046	T/G	0.076	-0.046	0.007	3.2e-10	KRT18-EIF4B
rs6458334	6	43354522	G/A	0.214	-0.031	0.005	3.2e-12	ZNF318-ABCC10
rs6533514	4	111337840	A/G	0.112	0.044	0.006	3.2e-13	ELOVL6-ENPEP
rs7089353	10	97276617	C/T	0.166	-0.021	0.005	3.3e-05	SORBS1
rs10744832	12	115178455	T/C	0.395	-0.017	0.004	3.3e-05	TBX3-MED13L
rs16953475	15	63348087	A/G	0.473	0.016	0.004	3.3e-05	TPM1
rs8026900	15	69424568	G/A	0.356	-0.017	0.004	3.3e-05	EWSAT1-GLCE
rs76187824	1	93833994	G/A	0.023	0.05	0.012	3.3e-05	DR1
rs2065703	20	31966698	C/T	0.154	0.021	0.005	3.3e-05	CDK5RAP1
rs744540	2	26804143	T/C	0.358	0.017	0.004	3.3e-05	CIB4,FAM166C
rs76964762	4	109246447	G/A	0.164	0.021	0.005	3.3e-05	LEF1-AS1-RPL34-AS1
rs6451305	5	36719979	C/T	0.236	-0.018	0.004	3.3e-05	SLC1A3-NIPBL-DT
rs34618835	6	37899784	C/T	0.056	0.033	0.008	3.3e-05	ZFAND3
rs2787604	10	120392439	T/C	0.331	0.018	0.004	3.3e-06	PRLHR-CACUL1
rs79042862	16	86530719	G/A	0.039	0.044	0.01	3.3e-06	FENDRR
rs1285679	1	232711884	A/C	0.497	0.017	0.004	3.3e-06	SIPA1L2-LINC01745
rs61014091	7	106810107	A/C	0.203	0.022	0.005	3.3e-06	HBP1
rs72747538	9	109880321	G/A	0.161	-0.025	0.005	3.3e-06	LOC340512-RAD23B
rs794356	7	75196531	G/A	0.428	0.019	0.004	3.3e-07	HIP1

SNPid	CHR	pos	Effect/other alleles	Effect allele Freq	beta	SE	pval	closest gene
rs117872640	11	100572709	T/C	0.031	0.056	0.01	3.3e-08	ARHGAP42
rs4872453	8	26413989	T/C	0.253	-0.026	0.004	3.3e-09	DPYSL2
rs117464403	10	107158054	G/A	0.018	-0.085	0.014	3.3e-10	SORCS3-LINC02627
rs2823139	21	16576783	G/A	0.336	-0.026	0.004	3.3e-11	NRIP1-USP25
rs17192317	12	90269777	C/T	0.05	-0.035	0.009	3.4e-05	ATP2B1-AS1-LINC02399
rs34087264	16	70323126	C/T	0.419	-0.015	0.004	3.4e-05	AARS1
rs11165084	1	94596302	C/T	0.371	-0.015	0.004	3.4e-05	ABCA4-ARHGAP29
rs6087840	20	30819912	C/T	0.047	0.035	0.009	3.4e-05	POFUT1
rs56060408	2	110041781	A/G	0.048	0.036	0.009	3.4e-05	SH3RF3
rs10034519	4	105410510	A/C	0.389	0.016	0.004	3.4e-05	CXXC4-AS1,CXXC4
rs2753254	6	5323156	G/T	0.374	-0.016	0.004	3.4e-05	FARS2
rs10985908	9	125986335	C/T	0.07	0.03	0.007	3.4e-05	STRBP
rs10841385	12	20007757	G/A	0.188	0.021	0.005	3.4e-06	AEBP2-LINC02398
rs1966500	19	18735666	T/C	0.452	0.018	0.004	3.4e-06	TMEM59L,TMEM59L-KLHL26
rs4651223	1	184580250	C/T	0.315	-0.019	0.004	3.4e-06	C1orf21
rs6576704	1	84892014	T/C	0.182	0.023	0.005	3.4e-06	DNASE2B-RPF1
rs7791727	7	106420850	T/C	0.489	-0.018	0.004	3.4e-06	CCDC71L-PIK3CG
rs290102	11	31015497	C/T	0.432	0.019	0.004	3.4e-07	DCDC1
rs61751344	11	9771457	C/T	0.017	0.079	0.014	3.4e-08	SWAP70,LINC02709
rs62188121	2	162232085	T/C	0.078	0.038	0.007	3.4e-08	PSMD14
rs73275538	8	102801104	T/C	0.028	0.07	0.011	3.4e-10	NCALD
rs1722883	7	134215403	T/C	0.476	0.026	0.004	3.4e-11	AKR1B10
rs2906163	7	2516594	C/T	0.349	0.027	0.004	3.4e-11	GRIFIN,GRIFIN-LFNG
rs1509966	10	64552607	G/A	0.497	0.031	0.004	3.4e-16	ZNF365-ADO
rs12570331	10	14253783	A/G	0.335	-0.016	0.004	3.5e-05	FRMD4A,FRMD4A
rs6583954	10	96534263	C/T	0.156	-0.022	0.005	3.5e-05	CYP2C19
rs72943494	11	77253788	G/A	0.107	-0.026	0.006	3.5e-05	PAK1-LOC646029
rs17554709	15	86046751	G/T	0.198	-0.02	0.005	3.5e-05	AKAP13
rs28750179	16	20551855	A/G	0.144	0.023	0.005	3.5e-05	ACSM2B
rs3218784	18	51807260	A/G	0.027	-0.048	0.011	3.5e-05	POLI
rs9960465	18	59359219	C/T	0.212	0.019	0.005	3.5e-05	CDH20-LINC01544
rs118117859	19	50159143	G/T	0.028	-0.043	0.011	3.5e-05	IRF3,SCAF1
rs12723373	1	230829847	G/A	0.142	0.022	0.005	3.5e-05	COG2,COG2-AGT
rs6713053	2	121350592	T/C	0.449	0.016	0.004	3.5e-05	LINC01101-GLI2
rs3772047	2	241666628	G/A	0.249	-0.018	0.004	3.5e-05	KIF1A
rs62140884	2	47776391	C/A	0.019	0.057	0.014	3.5e-05	KCNK12
rs13075615	3	136505832	C/T	0.145	0.022	0.005	3.5e-05	STAG1-SLC35G2
rs6779918	3	161540640	T/C	0.435	0.015	0.004	3.5e-05	OTOL1-LINC01192
rs2237002	4	2653847	A/C	0.221	0.019	0.005	3.5e-05	FAM193A
rs12215155	6	125658475	G/A	0.468	0.016	0.004	3.5e-05	HDDC2-LINC02523
rs77133138	6	26136687	C/A	0.02	-0.053	0.013	3.5e-05	H2AC6-H1-4

SNPid	CHR	pos	Effect/other alleles	Effect allele Freq	beta	SE	pval	closest gene
rs34165624	7	1666394	A/G	0.051	-0.036	0.009	3.5e-05	TFAMP1-ELFN1
rs2436851	8	103875613	A/G	0.256	0.017	0.004	3.5e-05	AZIN1-AS1,AZIN1
rs73227659	8	26545568	C/T	0.021	-0.05	0.013	3.5e-05	DPYSL2-ADRA1A
rs2488136	10	18334521	A/G	0.298	0.018	0.004	3.5e-06	SLC39A12,SLC39A12-CACNB2
rs15876	12	121867492	G/A	0.107	-0.028	0.006	3.5e-06	KDM2B
rs2229840	12	124826462	C/T	0.159	0.024	0.005	3.5e-06	NCOR2,MIR6880
rs12953087	17	59265201	C/T	0.207	0.02	0.005	3.5e-06	BCAS3
rs144760922	19	16507337	C/T	0.03	0.048	0.011	3.5e-06	EPS15L1
rs111802397	1	25252652	C/T	0.037	-0.044	0.01	3.5e-06	RUNX3
rs8116954	20	10937836	C/T	0.073	-0.033	0.007	3.5e-06	LOC101929413-C20orf187
rs4704768	5	157500760	G/A	0.2	0.022	0.005	3.5e-06	CLINT1-LINC02227
rs32497	5	55641055	C/T	0.196	0.021	0.005	3.5e-06	ANKRD55-LINC01948
rs10258119	7	48755648	C/T	0.44	-0.018	0.004	3.5e-06	ABCA13-CDC14C
rs3809272	12	111800258	G/A	0.3	0.021	0.004	3.5e-07	PHETA1
rs1175650	1	113019574	T/C	0.141	-0.03	0.005	3.5e-08	WNT2B
rs4423794	3	150175996	G/A	0.376	-0.02	0.004	3.5e-08	TSC22D2
rs55833661	8	26022471	A/G	0.262	0.024	0.004	3.5e-08	EBF2-PPP2R2A
rs618291	11	100681211	G/A	0.478	0.023	0.004	3.5e-09	ARHGAP42
rs1859551	14	72165208	A/G	0.472	-0.023	0.004	3.5e-10	SIPA1L1
rs11040531	11	49803119	A/G	0.13	0.04	0.006	3.5e-13	LOC440040
rs11130381	3	53850005	C/T	0.466	-0.03	0.004	3.5e-15	CHDH,CACNA1D
rs6271	9	136522274	C/T	0.071	0.06	0.007	3.5e-17	DBH,DBH-AS1
rs76135585	11	55191032	C/T	0.092	-0.027	0.006	3.6e-05	OR4A15-OR4C15
rs11611673	12	121451150	A/G	0.026	-0.05	0.012	3.6e-05	C12orf43
rs377371	12	42000177	T/C	0.473	-0.015	0.004	3.6e-05	PDZRN4-LINC02400
rs62016913	15	70975092	T/C	0.042	0.038	0.009	3.6e-05	UACA
rs6735011	2	53852102	T/C	0.336	0.016	0.004	3.6e-05	MIR4431-ASB3
rs13130330	4	36249796	T/C	0.394	0.016	0.004	3.6e-05	ARAP2,LOC439933
rs12641981	4	45179883	C/T	0.429	-0.016	0.004	3.6e-05	GNPDA2-GABRG1
rs382182	6	15819385	C/T	0.422	-0.015	0.004	3.6e-05	DTNBP1-MYLIP
rs2395694	6	38144269	A/G	0.064	0.031	0.008	3.6e-05	BTBD9
rs62411916	6	57644697	C/T	0.239	0.018	0.004	3.6e-05	PRIM2-GUSBP4
rs2205760	6	96786690	T/C	0.134	0.022	0.006	3.6e-05	FUT9-UFL1
rs8053979	16	89871168	A/G	0.187	-0.022	0.005	3.6e-06	FANCA
rs6682230	1	11999556	G/A	0.096	-0.029	0.006	3.6e-06	PLOD1
rs3093653	1	25683092	G/A	0.101	0.027	0.006	3.6e-06	TMEM50A
rs78085960	1	28736113	C/T	0.103	-0.028	0.006	3.6e-06	PHACTR4
rs78435371	2	161529181	A/G	0.206	0.022	0.005	3.6e-06	RBMS1-TANK
rs10818758	9	125652429	A/G	0.134	0.026	0.006	3.6e-06	RC3H2
rs7116121	11	16697527	G/T	0.215	-0.022	0.005	3.6e-07	SOX6
rs57334905	19	46368757	T/G	0.156	-0.026	0.005	3.6e-07	SYMPK,FOXA3

SNPid	CHR	pos	Effect/other alleles	Effect allele Freq	beta	SE	pval	closest gene
rs17581380	1	149854324	T/C	0.044	0.046	0.009	3.6e-07	H2AC20,H2BC21,H2AC21,H4C14-H2BC21
rs4307614	1	234097324	A/G	0.226	-0.023	0.005	3.6e-07	SLC35F3
rs12138348	1	45939136	C/T	0.087	-0.034	0.007	3.6e-07	TESK2
rs73872717	3	141134569	C/T	0.046	-0.045	0.009	3.6e-07	ZBTB38
rs11936781	4	86744838	G/A	0.11	-0.031	0.006	3.6e-07	ARHGAP24
rs10477516	5	113884069	G/A	0.115	0.03	0.006	3.6e-07	LOC101927078
rs1106090	2	58068741	G/A	0.373	0.023	0.004	3.6e-09	CCDC85A-VRK2
rs71636784	1	27169200	T/G	0.158	-0.033	0.005	3.6e-10	ZDHHC18
rs28650790	5	55861464	C/T	0.19	-0.03	0.005	3.6e-10	C5orf67
rs2764379	10	96159614	A/C	0.188	0.033	0.005	3.6e-12	TBC1D12,NOC3L-TBC1D12
rs222852	17	7140606	A/G	0.435	0.027	0.004	3.6e-13	DVL2,GABARAP,PHF23
rs72841270	10	104642237	T/G	0.138	0.041	0.006	3.6e-14	BORCS7-ASMT,AS3MT
rs2419922	5	157982756	T/G	0.301	-0.033	0.004	3.6e-15	LINC02227-EBF1
rs17419291	5	87780432	T/C	0.086	0.05	0.007	3.6e-15	LINC02060-LINC00461
rs6504608	17	47424681	C/A	0.362	-0.037	0.004	3.6e-22	ZNF652
rs2334385	11	1943708	C/T	0.234	-0.018	0.004	3.7e-05	TNNT3
rs138893177	15	44297617	C/T	0.024	-0.051	0.012	3.7e-05	FRMD5
rs7662107	4	104258396	C/T	0.286	0.016	0.004	3.7e-05	CENPE-LINC02428
rs2440406	8	6348204	A/G	0.26	-0.018	0.004	3.7e-05	MCPH1
rs7031064	9	14455076	A/G	0.479	0.015	0.004	3.7e-05	NFIB
rs62550596	9	18668939	T/C	0.142	0.021	0.005	3.7e-05	ADAMTSL1
rs7187736	16	51488206	A/G	0.226	-0.021	0.005	3.7e-06	SALL1-LINC01571
rs117586265	16	89698793	C/T	0.039	-0.043	0.01	3.7e-06	DPEP1
rs6056006	20	8666771	G/A	0.441	-0.017	0.004	3.7e-06	PLCB1
rs7808731	7	7318669	T/C	0.439	-0.018	0.004	3.7e-06	LOC101927354,LOC101927354-COL28A1
rs62188616	2	207957820	T/C	0.11	-0.033	0.006	3.7e-08	KLF7
rs11612002	12	50824232	A/G	0.214	0.027	0.005	3.7e-09	LARP4
rs12324159	15	41462468	G/A	0.435	-0.03	0.004	3.7e-15	INO80-EXD1
rs59980837	1	115827266	G/T	0.019	-0.134	0.014	3.7e-20	NGF,NGF-AS1
rs13394970	2	26929282	T/G	0.395	0.06	0.004	3.7e-56	KCNK3
rs34227136	11	27266122	G/A	0.41	0.016	0.004	3.8e-05	BBOX1-AS1-CCDC34
rs4091998	12	27367146	G/A	0.271	0.017	0.004	3.8e-05	C12orf71-STK38L
rs11168326	12	48345666	T/C	0.196	0.019	0.005	3.8e-05	VDR-TMEM106C
rs35349730	12	89998019	A/G	0.013	0.066	0.015	3.8e-05	ATP2B1
rs72648719	13	99252699	G/T	0.368	-0.015	0.004	3.8e-05	STK24-AS1-SLC15A1
rs28426081	14	104626997	G/A	0.469	0.016	0.004	3.8e-05	KIF26A
rs7517566	1	150850035	A/G	0.084	-0.028	0.007	3.8e-05	ARNT,ARNT-CTXND2
rs2250860	20	44184375	T/C	0.424	0.015	0.004	3.8e-05	WFDC8
rs6019409	20	47380990	T/C	0.338	-0.016	0.004	3.8e-05	PREX1
rs2528634	2	159592486	A/G	0.402	-0.016	0.004	3.8e-05	PKP4-AS1,PKP4-AS1-DAPL1

SNPid	CHR	pos	Effect/other alleles	Effect allele Freq	beta	SE	pval	closest gene
rs832790	5	76568292	C/A	0.401	0.016	0.004	3.8e-05	PDE8B
rs36044713	7	123532474	T/C	0.102	0.024	0.006	3.8e-05	HYAL4-SPAM1
rs61851721	10	63349910	C/A	0.07	-0.035	0.007	3.8e-06	TMEM26-AS1-CABCOCO1
rs61218937	1	65961299	C/T	0.136	0.027	0.006	3.8e-06	LEPR
rs114731436	2	176154787	C/T	0.039	-0.044	0.01	3.8e-06	ATP5MC3-LNPK
rs10187622	2	188414161	T/C	0.171	0.023	0.005	3.8e-06	TFPI
rs7819550	8	74206582	G/A	0.193	0.022	0.005	3.8e-06	RPL7,RDH10,RPL7
rs10816399	9	109364146	G/T	0.238	-0.021	0.004	3.8e-06	MIR8081,MIR8081-LINC01505
rs10822153	10	65056813	C/A	0.47	0.021	0.004	3.8e-08	JMJD1C
rs6938091	6	20522290	A/G	0.204	0.025	0.005	3.8e-08	E2F3-CDKAL1
rs2222684	8	77234927	C/T	0.438	-0.022	0.004	3.8e-09	HNF4G-LINC01111
rs1243197	10	21970077	T/C	0.483	-0.023	0.004	3.8e-10	MLLT10
rs62187657	2	162488091	G/A	0.09	0.043	0.007	3.8e-11	SLC4A10
rs11227582	11	55995736	C/A	0.122	0.039	0.006	3.8e-12	OR5T2,OR5J2-OR5T2
rs2421648	3	169129651	T/C	0.456	0.034	0.004	3.8e-20	MECOM
rs2168518	15	75081078	G/A	0.349	0.047	0.004	3.8e-33	CSK,MIR4513
rs11593126	10	95743087	A/G	0.068	-0.031	0.007	3.9e-05	PIPSL-PLCE1
rs57497660	12	55059155	C/A	0.221	0.019	0.005	3.9e-05	DCD-MUCL1
rs11117487	16	86421270	G/A	0.15	-0.022	0.005	3.9e-05	LINC00917-FENDRR
rs74839095	18	73408206	G/A	0.02	0.055	0.013	3.9e-05	LINC01898
rs79938023	1	114361770	A/G	0.049	0.033	0.008	3.9e-05	AP4B1-AS1,PTPN22
rs12483377	21	46931109	G/A	0.086	0.027	0.007	3.9e-05	COL18A1,SLC19A1
rs73183332	22	50466407	C/T	0.037	-0.041	0.01	3.9e-05	TTLL8
rs12478187	2	164057936	C/T	0.043	0.038	0.009	3.9e-05	KCNH7-FIGN
rs1978573	2	185459544	C/T	0.299	0.016	0.004	3.9e-05	ZNF804A,MIR548AE1-ZNF804A
rs2269071	2	208019264	T/C	0.296	-0.017	0.004	3.9e-05	KLF7
rs188852956	6	6813411	G/A	0.014	0.065	0.016	3.9e-05	LY86-RREB1
rs8030814	15	100217186	C/A	0.495	-0.017	0.004	3.9e-06	MEF2A
rs9928164	16	71234413	C/T	0.325	0.018	0.004	3.9e-06	HYDIN
rs4685218	3	14894140	C/T	0.091	-0.029	0.007	3.9e-06	FGD5
rs7755075	6	126708470	A/G	0.169	0.025	0.005	3.9e-06	CENPW
rs77405793	7	117050513	T/G	0.027	-0.051	0.011	3.9e-06	ASZ1
rs77102566	8	23527412	G/A	0.108	0.028	0.006	3.9e-06	SLC25A37-NKX3-1
rs2501950	13	41878171	T/C	0.072	0.04	0.007	3.9e-08	MTRF1
rs73374119	18	1894783	A/G	0.143	-0.032	0.005	3.9e-09	LINC00470-METTL4
rs7917532	10	18373902	T/C	0.466	0.024	0.004	3.9e-11	SLC39A12-CACNB2
rs36071027	5	158444274	C/T	0.351	0.031	0.004	3.9e-15	EBF1
rs11002569	10	80223728	G/T	0.284	0.017	0.004	4.1e-05	LINC00595-ZMIZ1-AS1
rs764855	11	56610412	G/T	0.445	0.016	0.004	4.1e-05	MIR6128-LINC02735
rs75074181	12	52315590	C/T	0.083	0.028	0.007	4.1e-05	ACVRL1
rs12821619	12	8791281	A/G	0.054	0.034	0.008	4.1e-05	AICDA-MFAP5

SNPid	CHR	pos	Effect/other alleles	Effect allele Freq	beta	SE	pval	closest gene
rs72714280	14	63949500	T/C	0.197	0.019	0.005	4.1e-05	PPP2R5E
rs2156774	18	22136195	C/A	0.175	0.02	0.005	4.1e-05	HRH4-LINC01915
rs73570553	19	47774329	T/C	0.316	0.017	0.004	4.1e-05	CCDC9,INAFM1
rs373747	22	20155192	T/C	0.483	-0.016	0.004	4.1e-05	CCDC188-LOC284865
rs11690717	2	205093360	G/T	0.366	0.016	0.004	4.1e-05	ICOS-PARD3B
rs3814035	2	47185830	C/T	0.068	-0.029	0.007	4.1e-05	TTC7A
rs7650509	3	134170971	A/G	0.437	0.016	0.004	4.1e-05	MIR4788-ANAPC13
rs688359	6	160465291	G/A	0.374	0.017	0.004	4.1e-05	IGF2R
rs76379691	7	150429668	G/A	0.102	0.026	0.006	4.1e-05	GIMAP5,GIMAP1-GIMAP5
rs2201337	11	4694603	C/T	0.322	0.018	0.004	4.1e-06	OR51E1-OR51E2
rs11658590	17	18831252	T/C	0.093	0.029	0.006	4.1e-06	PRPSAP2
rs17055718	18	72550794	T/C	0.102	-0.029	0.006	4.1e-06	ZNF407
rs62382151	5	165387540	C/T	0.347	0.018	0.004	4.1e-06	LOC102546299-LINC01947
rs9848210	3	27470734	C/T	0.043	-0.047	0.009	4.1e-07	SLC4A7
rs11722472	4	144485337	G/A	0.205	0.024	0.005	4.1e-07	GUSBP5,GUSBP5-FREM3
rs1290071	5	158092505	A/G	0.354	0.02	0.004	4.1e-07	LINC02227-EBF1
rs702634	5	53271420	G/A	0.303	-0.021	0.004	4.1e-07	ARL15
rs2516049	6	32570400	T/C	0.207	-0.033	0.006	4.1e-07	HLA-DRB1-HLA-DQA1
rs1400003	2	182961271	C/T	0.216	0.024	0.005	4.1e-08	PPP1R1C
rs2853796	7	150703915	G/T	0.467	0.021	0.004	4.1e-08	NOS3
rs4823006	22	29451671	A/G	0.45	0.022	0.004	4.1e-09	ZNRF3,C22orf31
rs2372888	2	37460234	A/G	0.379	0.024	0.004	4.1e-09	CEBPZ,NDUFAF7
rs74140812	1	228113436	T/C	0.106	0.037	0.006	4.1e-10	WNT9A
rs1413969	6	79790982	A/G	0.381	-0.027	0.004	4.1e-12	PHIP,PHIP-HMGN3
rs73278319	10	76451952	A/G	0.046	-0.038	0.009	4.2e-05	ADK
rs58546246	2	98521823	C/A	0.382	-0.016	0.004	4.2e-05	TMEM131
rs34343839	6	12922734	C/T	0.335	-0.016	0.004	4.2e-05	PHACTR1
rs73148945	7	133063456	A/G	0.085	0.028	0.007	4.2e-05	EXOC4
rs2700894	7	36172787	G/A	0.259	0.017	0.004	4.2e-05	LOC101928618-EEPD1
rs4872174	8	23525211	C/T	0.316	0.015	0.004	4.2e-05	SLC25A37-NKX3-1
rs12086697	1	88623666	T/C	0.1	0.029	0.006	4.2e-06	LINC01364-PKN2-AS1
rs6062314	20	62409713	C/T	0.082	0.031	0.007	4.2e-06	ZBTB46
rs5762780	22	29151760	G/A	0.138	-0.026	0.006	4.2e-06	HSCB
rs10203824	2	191595850	A/C	0.274	0.019	0.004	4.2e-06	NAB1-GLS
rs36061734	6	91260054	T/C	0.054	0.038	0.008	4.2e-06	MAP3K7
rs28447194	8	11542590	G/A	0.098	0.029	0.006	4.2e-06	GATA4
rs2296779	9	113151713	T/C	0.223	0.021	0.005	4.2e-06	SVEP1
rs118080693	17	36920050	C/T	0.058	-0.043	0.008	4.2e-07	PIP4K2B,PSMB3
rs13162397	5	57010990	C/T	0.338	-0.02	0.004	4.2e-07	LOC101928505-LINC02225
rs6466874	7	123250169	C/T	0.307	0.02	0.004	4.2e-07	ASB15-AS1,ASB15

SNPid	CHR	pos	Effect/other alleles	Effect allele Freq	beta	SE	pval	closest gene
rs12142296	1	46541679	T/G	0.134	-0.031	0.005	4.2e-08	PIK3R3,LOC110117498-PIK3R3
rs17054192	6	127212478	A/G	0.078	0.04	0.007	4.2e-08	MIR588-RSPO3
rs12935539	16	51754991	T/C	0.225	0.028	0.005	4.2e-10	SALL1-LINC01571
rs7412	19	45412079	C/T	0.08	0.043	0.007	4.2e-10	APOE
rs10906396	10	13551876	C/T	0.322	-0.016	0.004	4.3e-05	BEND7
rs11030323	11	28421065	G/A	0.462	-0.015	0.004	4.3e-05	METTL15-MIR8068
rs34973257	13	30286305	G/A	0.058	-0.034	0.008	4.3e-05	LOC102723345-UBL3
rs7199888	16	1519674	G/A	0.154	-0.022	0.005	4.3e-05	CLCN7
rs16978350	18	42940499	G/T	0.263	0.017	0.004	4.3e-05	SLC14A2
rs13001293	2	164568301	C/T	0.046	0.036	0.009	4.3e-05	FIGN
rs10102398	8	76670707	G/A	0.182	-0.019	0.005	4.3e-05	HNF4G-LINC01111
rs1418633	10	31975384	G/T	0.194	0.022	0.005	4.3e-06	ZEB1-ARHGAP12
rs8031550	15	80956407	G/A	0.246	0.019	0.004	4.3e-06	ARNT2-ABHD17C
rs16952251	16	53483138	A/G	0.31	0.019	0.004	4.3e-06	RBL2
rs80214639	3	23349062	A/G	0.064	-0.036	0.008	4.3e-06	UBE2E2
rs4676525	3	39902069	A/G	0.4	0.017	0.004	4.3e-06	MYRIP
rs4691869	4	156637094	T/C	0.379	-0.017	0.004	4.3e-06	GUCY1A1
rs9459231	6	165287399	C/T	0.329	0.019	0.004	4.3e-06	MEAT6-C6orf118
rs4549685	7	39326478	C/T	0.332	0.019	0.004	4.3e-06	POU6F2
rs4339320	5	101133279	A/C	0.243	-0.022	0.004	4.3e-07	ST8SIA4-SLCO4C1
rs12712867	2	43135815	A/G	0.495	0.024	0.004	4.3e-11	HAAO-LINC01819
rs7697171	4	103885504	C/T	0.491	0.029	0.004	4.3e-15	SLC9B1
rs7134677	12	54441498	C/T	0.295	0.04	0.004	4.3e-22	HOXC4
rs17341886	10	4128511	A/G	0.074	0.029	0.007	4.4e-05	LOC101927964
rs10994981	10	63708007	C/A	0.498	0.015	0.004	4.4e-05	ARID5B
rs672396	11	31386165	A/G	0.357	0.015	0.004	4.4e-05	DCDC1
rs10774459	12	886503	C/T	0.228	-0.018	0.005	4.4e-05	WNK1
rs79590310	15	77842095	A/G	0.424	-0.016	0.004	4.4e-05	LOC101929457-LINGO1
rs8045055	16	55944009	A/G	0.49	0.016	0.004	4.4e-05	CES5A
rs2958002	18	48039881	T/G	0.182	0.021	0.005	4.4e-05	SKA1-MAPK4
rs6745716	2	18882100	G/A	0.489	-0.015	0.004	4.4e-05	NT5C1B-RDH14-LINC01376
rs10033436	4	25388018	G/A	0.369	0.016	0.004	4.4e-05	ANAPC4
rs6912083	6	137723291	C/T	0.348	-0.017	0.004	4.4e-05	IFNGR1-OLIG3
rs11155202	6	142092295	G/A	0.051	0.036	0.009	4.4e-05	MIR4465-NMBR
rs74623670	9	95591603	A/C	0.201	0.019	0.005	4.4e-05	ANKRD19P
rs11024074	11	16917219	T/C	0.302	-0.019	0.004	4.4e-06	PLEKHA7
rs2290430	15	60791954	A/C	0.094	-0.031	0.006	4.4e-06	RORA-AS1,RORA
rs76667377	18	31138815	A/G	0.075	-0.033	0.007	4.4e-06	CCDC178-ASXL3
rs9846767	3	14880436	C/T	0.073	-0.032	0.007	4.4e-06	FGD5
rs17796841	7	23353514	T/C	0.061	0.035	0.008	4.4e-06	MALSU1,IGF2BP3
rs3811640	2	112776979	C/A	0.279	0.021	0.004	4.4e-07	MERTK

SNPid	CHR	pos	Effect/other alleles	Effect allele Freq	beta	SE	pval	closest gene
rs10931012	2	183290028	G/A	0.439	-0.019	0.004	4.4e-07	PDE1A
rs12587821	14	51092554	A/G	0.468	0.021	0.004	4.4e-08	ATL1
rs61169316	7	156321111	G/A	0.311	-0.024	0.004	4.4e-09	LINC01006
rs41219	14	71839807	T/C	0.469	-0.023	0.004	4.4e-10	SIPA1L1
rs302877	17	56764018	G/A	0.339	-0.024	0.004	4.4e-10	TEX14
rs79744918	2	43203447	G/A	0.077	0.042	0.007	4.4e-10	HAAO-LINC01819
rs11066395	12	113168961	C/A	0.434	0.027	0.004	4.4e-13	RPH3A
rs1075581	16	4135898	G/A	0.101	0.044	0.006	4.4e-13	ADCY9
rs4265150	8	129384895	C/A	0.373	0.028	0.004	4.4e-13	MIR1208-LINC00824
rs75938527	12	6838054	C/T	0.085	-0.026	0.007	4.5e-05	COPS7A
rs72744945	15	56931193	A/C	0.096	-0.026	0.006	4.5e-05	ZNF280D
rs6817875	4	57526349	A/C	0.361	0.017	0.004	4.5e-05	HOPX
rs72771783	5	97951616	C/A	0.162	0.021	0.005	4.5e-05	LINC01846-RGMB
rs76376137	6	34173330	T/G	0.049	-0.036	0.009	4.5e-05	GRM4-HMGA1
rs62074441	17	78236436	T/C	0.48	0.017	0.004	4.5e-06	RNF213
rs34433149	18	35813142	G/T	0.479	-0.017	0.004	4.5e-06	MIR4318-MIR924HG
rs62205904	20	42748368	G/A	0.117	-0.027	0.006	4.5e-06	JPH2
rs12533831	7	150870626	C/A	0.231	-0.02	0.004	4.5e-06	ASB10,GBX1-ASB10
rs12715275	3	35572591	A/G	0.289	-0.02	0.004	4.5e-07	LOC101928135-ARPP21
rs62431110	6	140510259	C/T	0.149	-0.027	0.005	4.5e-07	LOC100507477-MIR3668
rs137923903	21	44829595	C/T	0.013	0.096	0.017	4.5e-09	SIK1B,SIK1,LINC01679-SIK1
rs2144186	20	10828267	A/C	0.338	-0.036	0.004	4.5e-20	LINC01752-LOC101929413
rs61864670	10	64620357	T/C	0.053	-0.032	0.008	4.6e-05	EGR2-NRBF2
rs7132437	12	26502619	C/T	0.163	0.021	0.005	4.6e-05	ITPR2
rs72713672	14	102162027	A/G	0.07	0.03	0.007	4.6e-05	DIO3-LINC00239
rs12051	17	46103760	A/G	0.382	0.016	0.004	4.6e-05	COPZ2
rs73040793	19	30364926	G/A	0.063	0.032	0.008	4.6e-05	CCNE1-URI1
rs343953	2	45001464	G/A	0.184	-0.02	0.005	4.6e-05	CAMKMT,CAMKMT-LINC01833
rs11739752	5	38638424	T/C	0.488	-0.015	0.004	4.6e-05	LIFR-AS1
rs4236058	6	38442448	A/C	0.338	-0.016	0.004	4.6e-05	BTBD9
rs13214696	6	50259156	C/T	0.154	0.021	0.005	4.6e-05	DEFB112-TFAP2D
rs17089296	8	23349637	A/C	0.131	0.023	0.006	4.6e-05	ENTPD4-SLC25A37
rs7894582	10	115704458	C/A	0.044	0.044	0.009	4.6e-06	NHLRC2-ADRB1
rs66704117	12	109901330	C/T	0.058	-0.036	0.008	4.6e-06	KCTD10
rs9805764	13	31776418	C/T	0.292	-0.019	0.004	4.6e-06	B3GLCT
rs17577611	15	86299707	C/T	0.131	-0.024	0.005	4.6e-06	KLHL25,LOC101929679
rs62034688	16	55212449	T/G	0.107	0.027	0.006	4.6e-06	IRX5-IRX6
rs12452064	17	44868187	G/A	0.44	0.017	0.004	4.6e-06	WNT3
rs264226	18	10892951	A/C	0.42	-0.017	0.004	4.6e-06	PIEZO2
rs77194403	20	36260585	G/A	0.074	-0.033	0.007	4.6e-06	LINC00489-LOC100287792

SNPid	CHR	pos	Effect/other alleles	Effect allele Freq	beta	SE	pval	closest gene
rs16903196	5	87801670	A/G	0.097	-0.029	0.006	4.6e-06	LINC02060-LINC00461
rs10085375	7	17163262	A/C	0.382	0.018	0.004	4.6e-06	AGR3-AHR
rs12376605	9	139518718	A/G	0.212	-0.021	0.005	4.6e-06	LINC01451-HSPC324
rs6592142	11	83509773	C/T	0.434	-0.02	0.004	4.6e-07	DLG2
rs11623535	14	72462381	G/A	0.255	-0.022	0.004	4.6e-07	RGS6
rs61823391	1	218541992	T/C	0.314	-0.02	0.004	4.6e-07	TGFB2
rs11683207	2	98333290	T/C	0.15	-0.027	0.005	4.6e-07	ZAP70
rs62282972	3	178379501	C/T	0.051	0.044	0.009	4.6e-07	KCNMB2-AS1,KCNMB2
rs2680707	17	56455641	T/C	0.205	0.027	0.005	4.6e-09	RNF43
rs67290665	1	88922518	T/C	0.3	-0.024	0.004	4.6e-09	LINC01364-PKN2-AS1
rs954768	5	3706197	G/T	0.26	-0.027	0.004	4.6e-10	IRX1-LINC02114
rs17181782	8	105977743	T/C	0.183	0.039	0.005	4.6e-16	LRP12-ZFPM2
rs976785	10	18705074	C/T	0.326	0.042	0.004	4.6e-25	CACNB2
rs7187250	16	53810546	C/A	0.392	-0.041	0.004	4.6e-26	FTO
rs2289903	13	25273140	T/C	0.256	-0.018	0.004	4.7e-05	ATP12A
rs10910836	1	145646645	G/A	0.222	-0.018	0.004	4.7e-05	NBPF20,NBPF19,NBPF10,RNF115
rs56411234	1	150532376	G/A	0.032	-0.042	0.01	4.7e-05	ADAMTSL4,ADAMTSL4-AS1
rs59657202	1	228104559	C/T	0.189	-0.02	0.005	4.7e-05	WNT9A,PRSS38-WNT9A
rs2312459	2	113001329	A/G	0.296	-0.017	0.004	4.7e-05	ZC3H8
rs72814617	2	79147928	G/A	0.13	0.022	0.006	4.7e-05	LOC105374820-REG3G
rs62384157	5	131707537	A/G	0.172	-0.02	0.005	4.7e-05	MIR3936HG,SLC22A5
rs6928334	6	131411586	A/G	0.268	-0.017	0.004	4.7e-05	EPB41L2-AKAP7
rs17054363	6	151746586	T/C	0.137	-0.022	0.006	4.7e-05	RMND1
rs4869737	6	151892135	C/T	0.303	0.017	0.004	4.7e-05	CCDC170
rs17611963	7	122520343	C/T	0.055	0.032	0.008	4.7e-05	CADPS2
rs1568731	7	4665570	G/A	0.178	-0.02	0.005	4.7e-05	SDK1-FOXK1
rs6479789	10	63947969	T/C	0.176	0.022	0.005	4.7e-06	RTKN2,ARID5B-RTKN2
rs72655775	13	111019714	T/C	0.066	0.033	0.007	4.7e-06	COL4A2
rs4968281	17	44950122	T/C	0.262	-0.019	0.004	4.7e-06	WNT9B
rs897989	11	91973528	T/G	0.256	-0.022	0.004	4.7e-07	FAT3
rs9651825	12	27159784	A/G	0.268	-0.022	0.004	4.7e-07	TM7SF3
rs141094792	12	48113527	G/A	0.086	0.033	0.007	4.7e-07	ENDOU
rs7966391	12	57139056	G/A	0.367	0.02	0.004	4.7e-07	PRIM1
rs2113944	15	60977882	C/T	0.218	0.022	0.005	4.7e-07	RORA-AS2,RORA
rs8037584	15	95314639	A/G	0.138	-0.028	0.005	4.7e-07	MCTP2-LOC440311
rs2584681	17	47529746	A/G	0.262	-0.022	0.004	4.7e-07	PHB-LINC02075
rs74050116	11	2142184	G/A	0.081	-0.037	0.007	4.7e-08	H19-IGF2
rs17627481	12	111777482	G/A	0.049	0.049	0.009	4.7e-09	CUX2
rs9747171	17	45210873	A/G	0.402	-0.023	0.004	4.7e-10	CDC27
rs4755742	11	43810459	T/C	0.406	0.025	0.004	4.7e-11	HSD17B12

SNPid	CHR	pos	Effect/other alleles	Effect allele Freq	beta	SE	pval	closest gene
rs11097909	4	106911321	T/C	0.146	-0.038	0.005	4.7e-13	NPNT-LOC101929577
rs17477177	7	106411858	T/C	0.199	-0.042	0.005	4.7e-18	CCDC71L-PIK3CG
rs682681	13	22294062	T/C	0.32	-0.035	0.004	4.7e-19	FGF9-LINC00424
rs78304339	10	106580735	A/C	0.064	-0.031	0.008	4.8e-05	SORCS3
rs2891436	10	34552179	A/C	0.494	-0.014	0.004	4.8e-05	PARD3
rs1848802	11	56453566	C/T	0.486	-0.016	0.004	4.8e-05	OR5AR1-OR9G1
rs2238027	12	2216958	T/G	0.274	0.017	0.004	4.8e-05	CACNA1C
rs11620775	14	73275445	C/T	0.222	0.018	0.005	4.8e-05	DPF3
rs2856811	1	115838282	G/T	0.368	0.016	0.004	4.8e-05	NGF-AS1,NGF
rs6742014	2	68385955	C/T	0.352	-0.016	0.004	4.8e-05	WDR92,PNO1
rs140475729	5	157309275	C/T	0.042	0.038	0.009	4.8e-05	CLINT1-LINC02227
rs7100920	10	105640978	C/T	0.498	0.017	0.004	4.8e-06	STN1
rs112730974	10	63099406	C/T	0.031	-0.049	0.011	4.8e-06	LINC00845-TMEM26
rs695113	11	128562098	C/T	0.301	0.018	0.004	4.8e-06	FLI1,SENCR,FLI1
rs144255844	9	33863544	A/G	0.019	0.063	0.013	4.8e-06	UBE2R2
rs7276594	21	41288453	A/G	0.103	0.031	0.006	4.8e-07	PCP4
rs10077908	5	122503511	A/G	0.286	-0.023	0.004	4.8e-08	PRDM6
rs6869051	5	158345923	A/G	0.187	0.026	0.005	4.8e-08	EBF1
rs6905288	6	43758873	G/A	0.427	-0.02	0.004	4.8e-08	VEGFA,VEGFA-LINC02537
rs12205050	6	26111372	C/T	0.155	0.038	0.006	4.8e-12	H1-6,H1-6-H2BC4
rs1216744	11	100573092	C/T	0.235	-0.031	0.004	4.8e-13	ARHGAP42
rs10897138	11	55431897	C/T	0.127	0.04	0.006	4.8e-13	OR4C6,OR4S2-OR4C6
rs7000357	8	102829515	G/A	0.251	-0.033	0.004	4.8e-14	NCALD
rs11004585	10	56577441	T/C	0.392	0.016	0.004	4.9e-05	PCDH15
rs117911950	16	51342228	A/G	0.071	-0.03	0.007	4.9e-05	SALL1-LINC01571
rs4486390	1	2931440	C/T	0.107	0.024	0.006	4.9e-05	TTC34-ACTRT2
rs149106235	2	183376240	A/C	0.014	-0.064	0.016	4.9e-05	PDE1A
rs9488534	6	115428632	G/A	0.292	0.016	0.004	4.9e-05	HDAC2-AS2-LINC02534
rs2980867	8	126487691	T/G	0.294	0.017	0.004	4.9e-05	TRIB1-LINC00861
rs3794808	17	28531793	C/T	0.417	0.018	0.004	4.9e-06	SLC6A4
rs228726	1	7848943	G/A	0.094	0.028	0.006	4.9e-06	PER3
rs2353032	16	89385510	T/C	0.438	0.019	0.004	4.9e-07	LOC100287036,ANKRD11
rs7585434	2	177518748	T/C	0.419	-0.019	0.004	4.9e-07	LINC01117
rs11135910	8	25892142	C/T	0.163	-0.024	0.005	4.9e-07	EBF2
rs1093316	10	96108774	T/C	0.167	0.028	0.005	4.9e-08	NOC3L
rs4392188	2	144262757	A/G	0.121	-0.031	0.006	4.9e-08	ARHGAP15
rs72844204	6	28411468	A/G	0.044	0.068	0.013	4.9e-08	ZSCAN23,ZSCAN23-GPX6
rs72649639	8	51891208	A/G	0.178	-0.027	0.005	4.9e-08	SNTG1-PXDNL
rs73153123	20	62641994	G/A	0.164	-0.028	0.005	4.9e-09	PRPF6
rs331948	4	143679854	T/C	0.372	-0.026	0.004	4.9e-11	INPP4B
rs7105402	11	48411023	A/C	0.144	0.049	0.005	4.9e-20	OR4C5-OR4A47

SNPid	CHR	pos	Effect/other alleles	Effect allele Freq	beta	SE	pval	closest gene
rs62434125	6	151002371	T/C	0.075	0.086	0.007	4.9e-32	PLEKHG1,PLEKHG1
rs10840108	11	8703941	T/C	0.245	-0.018	0.004	5.1e-05	RPL27A,SNORA3A,SNORA3B,TRIM66-RPL27A
rs6707284	2	128736686	T/C	0.343	0.016	0.004	5.1e-05	SAP130
rs17522702	3	118452812	T/G	0.087	-0.028	0.007	5.1e-05	LOC105374060
rs2532861	3	53611136	A/G	0.026	-0.045	0.011	5.1e-05	CACNA1D
rs1042317	6	20492995	T/C	0.312	0.016	0.004	5.1e-05	E2F3
rs6940116	6	27708732	A/G	0.179	-0.03	0.007	5.1e-05	LINC01012-LOC100131289
rs302172	7	18317158	G/A	0.248	0.017	0.004	5.1e-05	HDAC9
rs2498857	9	109494498	T/C	0.29	-0.016	0.004	5.1e-05	LINC01505-ZNF462
rs11210838	1	43827190	C/A	0.14	-0.024	0.005	5.1e-06	ELOVL1,MIR6734,CDC20
rs72875755	2	37452045	C/T	0.043	0.041	0.009	5.1e-06	CEBPZ
rs41298968	5	137034169	C/T	0.183	0.022	0.005	5.1e-06	KLHL3
rs3807482	7	16727529	T/C	0.172	-0.022	0.005	5.1e-06	BZW2
rs113605702	12	50095636	G/A	0.086	0.033	0.007	5.1e-07	FMNL3
rs10950336	7	1753083	C/T	0.219	0.022	0.005	5.1e-07	ELFN1
rs34591516	8	142367087	C/T	0.047	-0.044	0.009	5.1e-07	GPR20
rs2478516	1	230868652	T/C	0.144	-0.03	0.005	5.1e-08	AGT-CAPN9
rs4507692	7	129670412	T/C	0.441	0.02	0.004	5.1e-08	ZC3HC1
rs114987861	3	53605712	G/A	0.024	-0.077	0.012	5.1e-11	CACNA1D
rs4778849	15	81026226	C/T	0.474	-0.028	0.004	5.1e-14	ABHD17C
rs76774446	17	45046368	C/A	0.128	-0.046	0.006	5.1e-18	MIR5089,GOSR2,GOSR2-MIR5089
rs598711	12	111973140	A/G	0.203	0.045	0.005	5.1e-22	ATXN2
rs601328	3	27547322	C/T	0.394	0.04	0.004	5.1e-25	SLC4A7-EOMES
rs61942582	12	110803794	G/A	0.072	0.029	0.007	5.2e-05	ATP2A2-ANAPC7
rs1963505	13	32908347	C/T	0.343	-0.016	0.004	5.2e-05	BRCA2
rs752004	15	74329021	T/C	0.493	-0.015	0.004	5.2e-05	PML
rs75506524	17	58231427	G/A	0.05	-0.033	0.009	5.2e-05	CA4
rs12465259	2	54498212	T/C	0.063	0.031	0.008	5.2e-05	ACYP2
rs13023231	2	64215748	C/T	0.406	-0.016	0.004	5.2e-05	VPS54
rs1053598	6	24704538	T/C	0.292	0.017	0.004	5.2e-05	ACOT13,C6orf62
rs858995	6	27181701	T/C	0.09	-0.036	0.009	5.2e-05	MIR3143-PRSS16
rs7356991	6	6998130	C/T	0.366	0.016	0.004	5.2e-05	LY86-RREB1
rs12859192	13	88799824	G/A	0.38	-0.018	0.004	5.2e-06	LINC00373
rs111299170	1	205674897	C/T	0.08	0.032	0.007	5.2e-06	SLC45A3-NUCKS1
rs1534696	7	26397239	C/A	0.455	0.017	0.004	5.2e-06	SNX10
rs58638214	19	31864938	C/T	0.394	-0.02	0.004	5.2e-07	TSHZ3-THEG5
rs11168236	12	48178604	T/C	0.142	-0.029	0.005	5.2e-08	SLC48A1,HDAC7
rs1080078	6	51834038	A/G	0.331	-0.023	0.004	5.2e-09	PKHD1
rs188315257	2	25241386	T/G	0.089	0.041	0.007	5.2e-10	DNAJC27-AS1
rs228632	4	103573904	C/T	0.494	-0.023	0.004	5.2e-10	MANBA

SNPid	CHR	pos	Effect/other alleles	Effect allele Freq	beta	SE	pval	closest gene
rs12505584	4	111416292	A/G	0.418	0.023	0.004	5.2e-10	ENPEP
rs1757463	15	41783814	A/G	0.283	-0.029	0.004	5.2e-12	ITPKA,RTF1-ITPKA
rs2566511	7	150703041	C/T	0.241	0.031	0.004	5.2e-13	NOS3
rs3790604	1	113046879	C/A	0.072	-0.08	0.007	5.2e-29	WNT2B,WNT2B
rs77055095	11	19221518	G/A	0.228	0.018	0.004	5.3e-05	CSRP3
rs62062196	17	29970056	T/C	0.027	-0.043	0.011	5.3e-05	MIR365B-COPRS
rs8074759	17	64326305	C/T	0.121	0.023	0.006	5.3e-05	PRKCA
rs1351844	19	45787002	G/A	0.355	0.016	0.004	5.3e-05	MARK4
rs7556473	1	15996858	A/G	0.245	0.017	0.004	5.3e-05	DDI2,DDI2-PLEKHM2
rs2275293	20	62576419	A/G	0.065	-0.029	0.007	5.3e-05	MIR1914,MIR647,UCKL1
rs12053541	2	144073821	A/G	0.31	-0.017	0.004	5.3e-05	ARHGAP15
rs1941929	2	158496502	A/C	0.149	-0.022	0.005	5.3e-05	ACVR1C-ACVR1
rs72655587	4	77195110	T/C	0.156	-0.021	0.005	5.3e-05	FAM47E-STBD1,FAM47E
rs1469688	5	138591955	A/G	0.081	-0.027	0.007	5.3e-05	SIL1-SNHG4
rs7736891	5	52808873	G/A	0.425	0.015	0.004	5.3e-05	FST-NDUFS4
rs12282356	11	113208382	A/G	0.372	0.018	0.004	5.3e-06	TTC12
rs1003136	5	127347815	G/A	0.364	0.018	0.004	5.3e-06	LINC01184
rs10486081	7	13740834	G/A	0.147	0.024	0.005	5.3e-06	ARL4A-ETV1
rs6470120	8	123881218	G/A	0.347	0.018	0.004	5.3e-06	ZHX2
rs4569188	14	93915929	G/A	0.341	-0.02	0.004	5.3e-07	UNC79
rs2602698	19	4937854	T/G	0.297	-0.025	0.004	5.3e-09	UHRF1
rs2216707	5	61951416	C/T	0.168	-0.031	0.005	5.3e-10	IPO11-HTR1A
rs10044570	5	32760773	G/T	0.11	-0.044	0.006	5.3e-13	NPR3
rs11196628	10	115850375	C/A	0.174	0.019	0.005	5.4e-05	ADRB1-CCDC186
rs77048156	12	111122466	C/T	0.032	0.041	0.011	5.4e-05	HVCN1
rs510634	20	60944314	G/T	0.149	-0.022	0.005	5.4e-05	LAMA5,MIR1-1HG-AS1
rs66531209	3	37894621	G/T	0.158	-0.02	0.005	5.4e-05	ITGA9-AS1
rs6781999	3	85734579	G/A	0.291	-0.017	0.004	5.4e-05	CADM2
rs10019711	4	102500252	G/A	0.213	-0.018	0.005	5.4e-05	FLJ20021-BANK1
rs286560	7	2552986	A/C	0.35	0.016	0.004	5.4e-05	LFNG
rs13440002	9	34583636	G/T	0.48	0.015	0.004	5.4e-05	CNTFR-AS1,CNTFR
rs12941165	17	36844593	G/T	0.391	-0.018	0.004	5.4e-06	EPOP-LOC105371763
rs13403092	2	37987680	T/C	0.199	-0.021	0.005	5.4e-06	CDC42EP3-LINC00211
rs1848510	5	57754005	G/A	0.366	-0.018	0.004	5.4e-06	PLK2
rs7775435	6	52252949	G/A	0.407	0.018	0.004	5.4e-06	PAQR8
rs1500876	12	32659277	G/A	0.343	-0.021	0.004	5.4e-08	FGD4
rs13293465	9	37127678	C/T	0.349	0.024	0.004	5.4e-10	ZCCHC7
rs74163354	10	126842060	G/A	0.105	-0.024	0.006	5.5e-05	CTBP2
rs7121067	11	63689879	T/C	0.43	-0.014	0.004	5.5e-05	RCOR2-NAA40
rs254326	16	83747850	G/A	0.31	0.016	0.004	5.5e-05	CDH13
rs4849453	2	117011679	G/A	0.466	-0.016	0.004	5.5e-05	DPP10-DDX18

SNPid	CHR	pos	Effect/other alleles	Effect allele Freq	beta	SE	pval	closest gene
rs12543107	8	55268833	A/G	0.054	-0.035	0.008	5.5e-05	RNU105C-SOX17
rs11696516	20	50131816	A/C	0.451	0.019	0.004	5.5e-07	NFATC2
rs10158347	1	232529744	A/G	0.284	0.022	0.004	5.5e-08	SIPA1L2,TSNAX-DISC1-SIPA1L2
rs1033661	20	51024515	C/T	0.371	0.021	0.004	5.5e-08	ZFP64-LINC01524
rs511154	3	135950921	A/G	0.234	-0.03	0.004	5.5e-11	MSL2-PCCB
rs10850407	12	115378037	T/G	0.301	0.03	0.004	5.5e-14	TBX3-MED13L
rs9532979	13	42621017	A/G	0.308	0.016	0.004	5.6e-05	DGKH,DGKH
rs6496235	15	86343428	T/C	0.245	-0.018	0.004	5.6e-05	KLHL25-MIR548AP
rs11640600	16	82815659	T/C	0.35	0.016	0.004	5.6e-05	CDH13,LOC101928446
rs28552491	18	72333691	C/T	0.133	-0.022	0.006	5.6e-05	LINC00909-ZNF407
rs2078337	1	112371071	A/C	0.335	-0.016	0.004	5.6e-05	KCND3
rs4373805	1	204175579	A/G	0.15	0.021	0.005	5.6e-05	GOLT1A
rs74887298	2	26929096	G/A	0.041	-0.039	0.01	5.6e-05	KCNK3
rs6445541	3	52880128	G/T	0.376	-0.015	0.004	5.6e-05	MIR8064,STIMATE-MUSTN1,STIMATE
rs62304263	4	42391677	T/C	0.22	-0.017	0.005	5.6e-05	LOC105374428
rs36027301	11	67809268	C/T	0.056	0.039	0.008	5.6e-07	TCIRG1,MIR6753
rs11169572	12	51216890	T/C	0.409	0.021	0.004	5.6e-08	ATF1,ATF1-TMPRSS12
rs12988578	2	142877109	A/G	0.497	0.021	0.004	5.6e-08	LRP1B
rs13163982	5	148026299	C/T	0.234	0.024	0.004	5.6e-08	HTR4
rs1485748	8	25870252	A/G	0.378	-0.021	0.004	5.6e-08	EBF2
rs2744126	6	22404140	G/T	0.191	0.033	0.005	5.6e-13	PRL-HDGFL1
rs11187793	10	95896716	G/A	0.415	-0.032	0.004	5.6e-17	PLCE1
rs1007530	11	119948049	G/A	0.162	0.021	0.005	5.7e-05	LOC102724301-TRIM29
rs11832532	12	98915601	G/A	0.053	0.034	0.009	5.7e-05	TMPO
rs9551769	13	30473681	A/C	0.058	0.033	0.008	5.7e-05	LINC00297-LINC00572
rs10146690	14	79890456	G/A	0.209	-0.019	0.005	5.7e-05	NRXN3
rs1111480	16	52521356	A/C	0.139	-0.023	0.005	5.7e-05	TOX3
rs2917708	16	64364892	G/A	0.257	0.017	0.004	5.7e-05	CDH8-CDH11
rs10518269	19	31028666	C/T	0.163	0.021	0.005	5.7e-05	ZNF536
rs60802415	1	233026749	C/T	0.048	-0.037	0.009	5.7e-05	MAP10-NTPCR
rs2457049	1	48041651	T/G	0.018	-0.053	0.014	5.7e-05	FOXD2-TRABD2B
rs9862733	3	125220246	C/T	0.453	-0.015	0.004	5.7e-05	SNX4
rs6580550	5	147856232	T/C	0.454	0.015	0.004	5.7e-05	HTR4,HTR4
rs11753254	6	51700849	T/C	0.069	-0.029	0.007	5.7e-05	PKHD1
rs17726513	6	80130318	A/C	0.191	-0.02	0.005	5.7e-05	LCAL1-LCA5
rs10954267	7	129933481	C/A	0.4	-0.016	0.004	5.7e-05	CPA2,CPA4
rs7929853	11	8238672	T/G	0.158	-0.024	0.005	5.7e-06	RIC3-LMO1
rs73196768	13	50730879	T/C	0.121	-0.025	0.006	5.7e-06	DLEU1
rs8112011	19	37730074	T/C	0.148	0.025	0.005	5.7e-06	ZNF383
rs11207196	1	58790501	C/T	0.194	0.022	0.005	5.7e-06	DAB1-OMA1
rs12516818	5	113799403	G/A	0.238	0.02	0.004	5.7e-06	KCNN2,LOC101927078

SNPid	CHR	pos	Effect/other alleles	Effect allele Freq	beta	SE	pval	closest gene
rs871668	8	19152067	T/G	0.414	-0.017	0.004	5.7e-06	LOC100128993-SH2D4A
rs77256758	2	43838963	G/A	0.019	0.065	0.013	5.7e-07	THADA-PLEKHH2
rs58821592	5	132399944	C/T	0.258	0.022	0.004	5.7e-07	HSPA4
rs7731717	5	173281685	T/C	0.297	0.022	0.004	5.7e-08	LINC01485-CPEB4
rs58583238	10	63572200	C/T	0.099	0.037	0.006	5.7e-09	CABCOCO1-ARID5B
rs116919616	11	57116046	C/T	0.023	0.069	0.012	5.7e-09	P2RX3
rs13125101	4	81174592	G/A	0.287	-0.092	0.004	5.7e-109	PRDM8-FGF5
rs112437639	11	65531612	G/A	0.016	0.106	0.015	5.7e-13	RNASEH2C-AP5B1
rs13354898	5	87256610	T/C	0.15	0.037	0.005	5.7e-13	LINC02488-TMEM161B
rs1574114	10	12448888	C/T	0.439	-0.015	0.004	5.8e-05	CAMK1D
rs11023781	11	15943742	T/C	0.07	-0.032	0.008	5.8e-05	LOC102724957-SOX6
rs187162129	11	45654244	A/G	0.025	-0.047	0.012	5.8e-05	LINC02687-CHST1
rs1915108	12	89991739	A/C	0.049	0.036	0.009	5.8e-05	ATP2B1
rs117674718	15	68960512	T/C	0.018	-0.056	0.014	5.8e-05	CORO2B
rs9894621	17	12000584	A/G	0.466	-0.015	0.004	5.8e-05	MAP2K4
rs77947172	20	62560203	G/A	0.037	0.038	0.01	5.8e-05	DNAJC5
rs73969936	2	167812982	A/G	0.031	-0.045	0.011	5.8e-05	XIRP2
rs358534	5	63230765	T/C	0.483	0.015	0.004	5.8e-05	IPO11-HTR1A
rs56204645	8	55421769	T/C	0.211	-0.018	0.005	5.8e-05	SOX17-RP1
rs4783279	16	82829780	C/T	0.157	-0.023	0.005	5.8e-06	CDH13,LOC101928446
rs1045384	19	10427246	G/T	0.176	0.022	0.005	5.8e-06	RAVER1,FDX2
rs34891	3	10525187	T/G	0.302	-0.018	0.004	5.8e-06	ATP2B2
rs9811721	3	111521268	T/C	0.304	-0.019	0.004	5.8e-06	PLCXD2,PHLDB2
rs113279217	7	71474895	G/A	0.154	0.024	0.005	5.8e-06	CALN1
rs7023954	9	21816758	G/A	0.418	-0.016	0.004	5.8e-06	MTAP
rs198683	6	143200547	C/T	0.457	0.02	0.004	5.8e-08	HIVEP2
rs12144044	1	113248791	C/A	0.271	0.025	0.004	5.8e-10	PPM1J,RHOC
rs2105092	6	134184972	G/A	0.297	0.034	0.004	5.8e-16	TARID
rs192204152	10	18715753	A/G	0.017	-0.059	0.014	5.9e-05	CACNB2
rs1948151	12	26459071	G/A	0.277	0.017	0.004	5.9e-05	SSPN-ITPR2
rs112462318	16	20399956	A/G	0.111	-0.025	0.006	5.9e-05	PDILT
rs7247576	19	1224999	A/G	0.267	0.018	0.004	5.9e-05	CBARP,STK11
rs12975217	19	2325297	T/C	0.042	-0.037	0.009	5.9e-05	SPPL2B,LSM7
rs1151623	20	62368606	T/C	0.247	-0.018	0.004	5.9e-05	SLC2A4RG,ZGPAT,LIME1
rs56396705	2	237107225	G/A	0.171	-0.019	0.005	5.9e-05	ASB18
rs141121949	4	42134531	G/A	0.045	-0.036	0.009	5.9e-05	BEND4
rs10028494	4	69970937	A/C	0.221	0.018	0.005	5.9e-05	UGT2B7
rs4958190	5	133897000	C/T	0.461	-0.016	0.004	5.9e-05	JADE2
rs11249593	5	178269126	T/C	0.305	0.017	0.004	5.9e-05	AACSP1-ZNF354B
rs269267	7	140372299	T/C	0.161	-0.02	0.005	5.9e-05	ADCK2,DENND2A-ADCK2
rs6955526	7	50638463	G/A	0.155	-0.021	0.005	5.9e-05	DDC-GRB10

SNPid	CHR	pos	Effect/other alleles	Effect allele Freq	beta	SE	pval	closest gene
rs10739694	9	130526605	A/G	0.414	-0.015	0.004	5.9e-05	SH2D3C
rs13189816	5	32989116	T/C	0.471	-0.018	0.004	5.9e-06	LINC02120-LINC02160
rs10782230	6	126228512	G/A	0.478	-0.017	0.004	5.9e-06	NCOA7
rs17108576	14	39399131	T/C	0.178	0.028	0.005	5.9e-08	LOC105370457-SEC23A
rs752208	2	25455389	G/A	0.207	-0.026	0.005	5.9e-08	DNMT3A,DNMT3A,DNMT3A
rs60600323	7	26422934	C/T	0.086	-0.037	0.007	5.9e-08	LOC105375304
rs2820292	1	201784287	A/C	0.442	-0.022	0.004	5.9e-10	NAV1,IPO9-AS1
rs34262842	16	20355811	A/G	0.161	0.054	0.005	5.9e-27	UMOD
rs7090117	10	125649465	T/C	0.112	0.024	0.006	6.1e-05	CPXM2
rs10519292	15	81077470	G/A	0.094	0.027	0.007	6.1e-05	CEMIP
rs2257181	16	8990680	C/A	0.311	-0.017	0.004	6.1e-05	USP7
rs16859665	3	184845544	A/G	0.283	-0.017	0.004	6.1e-05	C3orf70
rs111747606	6	7168946	A/G	0.014	-0.065	0.016	6.1e-05	RREB1
rs4723581	7	36870228	G/A	0.345	-0.015	0.004	6.1e-05	AOAH-ELMO1
rs12576071	11	43594337	C/T	0.092	0.03	0.007	6.1e-06	MIR670HG,MIR670HG-MIR129-2
rs7315861	12	18284908	C/A	0.388	-0.017	0.004	6.1e-06	RERGL-PIK3C2G
rs10930127	2	165332055	T/G	0.16	0.023	0.005	6.1e-06	FIGN-GRB14
rs6781619	3	25425217	A/G	0.152	-0.023	0.005	6.1e-06	RARB,RARB-AS1
rs3129308	4	3309272	G/A	0.402	-0.017	0.004	6.1e-06	MSANTD1-RGS12
rs930892	5	141721245	G/T	0.391	-0.017	0.004	6.1e-06	SPRY4-AS1
rs74612948	8	17779957	G/A	0.079	-0.034	0.007	6.1e-07	PCM1,FGL1-PCM1
rs55833772	8	26046347	A/G	0.103	-0.03	0.006	6.1e-07	EBF2-PPP2R2A
rs13333159	16	81568064	T/G	0.26	0.023	0.004	6.1e-08	MIR7854,CMIP
rs11920344	3	78722982	A/G	0.252	0.023	0.004	6.1e-08	ROBO1
rs17274750	21	16353809	A/C	0.094	-0.04	0.006	6.1e-10	NRIP1
rs10947786	6	39156410	G/A	0.209	0.028	0.005	6.1e-10	KCNK5,SAYSD1-KCNK5
rs7826620	8	23375652	C/T	0.324	-0.024	0.004	6.1e-10	ENTPD4-SLC25A37
rs2032447	6	26044369	A/G	0.309	-0.03	0.004	6.1e-12	H2BC3,H3C3,H2BC3-H3C3
rs12926550	16	81510155	G/A	0.31	0.031	0.004	6.1e-14	CMIP
rs11666569	19	17214073	C/T	0.294	-0.035	0.004	6.1e-17	MYO9B
rs1689040	12	89978233	C/T	0.409	0.05	0.004	6.1e-40	ATP2B1,POC1B-AS1-ATP2B1
rs115598727	10	85325065	C/T	0.021	-0.053	0.013	6.2e-05	NRG3-LINC02650
rs7929799	11	119055362	A/G	0.433	-0.015	0.004	6.2e-05	PDZD3,NLRX1,CCDC153,NLRX1-PDZD3
rs2270755	11	22770922	G/T	0.335	-0.016	0.004	6.2e-05	GAS2
rs7514373	1	96836927	C/T	0.035	0.039	0.01	6.2e-05	LINC01787
rs2836267	21	39644650	A/G	0.271	0.016	0.004	6.2e-05	KCNJ15
rs61093635	2	106127817	A/G	0.097	0.027	0.007	6.2e-05	FHL2-LOC285000
rs28381344	6	31707526	G/A	0.04	-0.052	0.013	6.2e-05	CLIC1,MSH5,MSH5-SAPCD1,CLIC1-MSH5
rs2960856	7	1206638	C/T	0.398	-0.016	0.004	6.2e-05	LOC101927021,LOC101927021-UNCX

SNPid	CHR	pos	Effect/other alleles	Effect allele Freq	beta	SE	pval	closest gene
rs79243129	7	142644474	G/A	0.018	-0.056	0.014	6.2e-05	KEL
rs4379368	7	40466200	C/T	0.108	0.024	0.006	6.2e-05	SUGCT
rs12823540	12	110308549	T/C	0.39	-0.018	0.004	6.2e-06	GLTP
rs16829482	1	193391298	G/T	0.194	-0.021	0.005	6.2e-06	LINC01031-LINC01724
rs6772037	3	39491929	A/G	0.499	-0.016	0.004	6.2e-06	RPSA-MOBP
rs1425521	4	143480525	T/C	0.014	0.074	0.017	6.2e-06	INPP4B
rs77726527	10	18481525	C/T	0.019	-0.07	0.014	6.2e-07	CACNB2
rs1262220	11	95385613	T/C	0.496	-0.019	0.004	6.2e-07	LOC100129203-FAM76B
rs73486469	12	133065832	A/G	0.137	-0.027	0.005	6.2e-07	FBRSL1,LOC101928416-FBRSL1
rs7160893	14	98607294	G/T	0.197	-0.023	0.005	6.2e-07	LINC01550-C14orf177
rs117074666	19	11504566	G/T	0.031	-0.055	0.011	6.2e-07	RGL3,EPOR-RGL3
rs114435241	10	18439324	G/A	0.065	-0.042	0.008	6.2e-08	CACNB2
rs833752	3	136255169	A/G	0.26	-0.027	0.004	6.2e-10	STAG1
rs4412193	6	26338056	A/G	0.37	0.027	0.004	6.2e-11	H4C8-BTN3A2
rs6686889	1	25030470	C/T	0.256	-0.029	0.004	6.2e-12	SRRM1-CLIC4
rs140552882	10	96135633	T/C	0.022	0.051	0.013	6.3e-05	NOC3L-TBC1D12
rs12423234	12	4930360	G/A	0.4	-0.015	0.004	6.3e-05	KCNA6
rs73119152	20	32143942	G/A	0.17	-0.021	0.005	6.3e-05	CBFA2T2
rs4639051	4	148448870	A/G	0.163	0.02	0.005	6.3e-05	EDNRA
rs2636617	6	133943732	C/A	0.395	-0.015	0.004	6.3e-05	TARID
rs17316802	6	153258876	C/T	0.201	0.018	0.005	6.3e-05	VIP-FBXO5
rs3846528	5	55697956	C/T	0.334	-0.018	0.004	6.3e-06	ANKRD55-LINC01948
rs9972911	17	47177747	C/T	0.388	0.019	0.004	6.3e-07	IGF2BP1-B4GALNT2
rs2051559	4	3298800	T/C	0.132	-0.027	0.006	6.3e-07	MSANTD1-RGS12
rs1558917	7	130028723	C/T	0.348	-0.02	0.004	6.3e-07	CPA1,CEP41,CPA1-CEP41
rs1630266	6	118612943	G/A	0.077	-0.041	0.007	6.3e-09	SLC35F1
rs9935429	16	2210407	G/A	0.056	-0.05	0.008	6.3e-10	TRAF7
rs1779383	10	44895357	T/G	0.186	-0.018	0.005	6.4e-05	CXCL12-TMEM72-AS1
rs78653555	15	53915110	C/A	0.061	0.032	0.008	6.4e-05	WDR72
rs4788828	16	71724090	C/A	0.167	-0.02	0.005	6.4e-05	PHLPP2
rs56325146	17	29151024	T/C	0.131	0.022	0.006	6.4e-05	CRLF3
rs9845524	3	29376642	G/T	0.252	0.017	0.004	6.4e-05	RBMS3
rs59319869	6	129462182	A/G	0.061	0.031	0.008	6.4e-05	LAMA2
rs117040556	7	41232994	C/T	0.106	-0.024	0.006	6.4e-05	LINC01449-INHBA
rs5762664	22	28900001	T/C	0.113	-0.027	0.006	6.4e-06	TTC28
rs11899825	2	20724694	G/T	0.432	0.017	0.004	6.4e-06	LOC107985856-HS1BP3-IT1
rs62137162	2	43090790	A/G	0.217	0.021	0.005	6.4e-06	HAAO-LINC01819
rs16881581	6	51431529	T/C	0.38	-0.018	0.004	6.4e-06	TFAP2B-PKHD1
rs455651	5	66190284	C/T	0.457	-0.02	0.004	6.4e-08	MAST4
rs202687	11	49180269	T/G	0.144	0.044	0.005	6.4e-17	FOLH1

SNPid	CHR	pos	Effect/other alleles	Effect allele Freq	beta	SE	pval	closest gene
rs73007637	11	100644268	C/T	0.046	-0.037	0.009	6.5e-05	ARHGAP42
rs10785445	12	43955387	T/C	0.017	-0.058	0.014	6.5e-05	ADAMTS20-PUS7L
rs10520129	15	39641885	A/G	0.42	-0.015	0.004	6.5e-05	C15orf54-THBS1
rs2830281	21	27929922	C/A	0.199	-0.019	0.005	6.5e-05	CYYR1-AS1,CYYR1
rs72830474	2	105414217	C/T	0.12	0.023	0.006	6.5e-05	LINC01114-PANTR1
rs415130	2	225916969	T/C	0.478	-0.015	0.004	6.5e-05	DOCK10-NYAP2
rs7701271	5	150776708	G/A	0.384	0.015	0.004	6.5e-05	SLC36A2-SLC36A1
rs116371977	6	31140068	C/T	0.03	0.046	0.012	6.5e-05	POU5F1,PSORS1C3,POU5F1-PSORS1C3
rs4376836	10	102415021	T/C	0.351	0.019	0.004	6.5e-06	HIF1AN-PAX2
rs12779698	10	92711293	A/G	0.405	0.018	0.004	6.5e-06	XLOC_008559
rs7336670	13	54199624	C/A	0.207	0.021	0.005	6.5e-06	LINC01065-LINC00558
rs12402966	1	172203894	G/A	0.21	-0.02	0.005	6.5e-06	DNM3
rs2194375	2	208246257	T/G	0.307	-0.018	0.004	6.5e-06	LINC01802-CREB1
rs114290391	4	111107721	A/C	0.045	0.042	0.009	6.5e-06	ELOVL6
rs75322996	5	113594885	C/T	0.115	0.026	0.006	6.5e-06	KCNN2
rs4540313	7	1849127	C/T	0.445	-0.017	0.004	6.5e-06	ELFN1-MAD1L1
rs10887611	10	88208698	A/C	0.49	0.019	0.004	6.5e-07	WAPL
rs7646407	3	167463532	T/G	0.222	-0.022	0.005	6.5e-07	SERPINI1
rs113776293	3	183988289	G/A	0.181	-0.024	0.005	6.5e-07	EEF1AKMT4-ECE2
rs2161228	5	88001798	C/T	0.103	-0.03	0.006	6.5e-07	MEF2C-AS2
rs1570061	6	26575986	T/C	0.47	0.024	0.005	6.5e-07	LOC105374988,LOC105374988-ABT1
rs1047720	9	14588910	G/A	0.292	-0.021	0.004	6.5e-07	NFIB-ZDHHC21
rs706848	2	234299919	G/A	0.434	0.021	0.004	6.5e-08	DGKD
rs6710055	2	43448658	A/G	0.382	0.02	0.004	6.5e-08	ZFP36L2,LINC02580-ZFP36L2
rs11692449	2	61704451	T/C	0.362	0.028	0.004	6.5e-13	XPO1,USP34-XPO1
rs62426328	6	127155481	G/A	0.433	-0.052	0.004	6.5e-42	MIR588-RSPO3
rs11047238	12	24207030	A/C	0.396	0.016	0.004	6.6e-05	SOX5
rs886528	16	3811556	A/G	0.446	0.015	0.004	6.6e-05	CREBBP
rs3924957	17	70645752	C/T	0.093	0.025	0.006	6.6e-05	SLC39A11
rs34275678	18	48943793	T/C	0.026	-0.048	0.012	6.6e-05	LINC01630
rs12028554	1	154223101	G/A	0.438	0.015	0.004	6.6e-05	UBAP2L
rs10204729	2	117892964	G/T	0.207	0.017	0.005	6.6e-05	DPP10-DDX18
rs7587314	2	23334047	A/G	0.494	0.014	0.004	6.6e-05	LINC01884-KLHL29
rs62378502	5	168242124	A/C	0.113	-0.023	0.006	6.6e-05	SLIT3
rs191785584	8	128183871	A/G	0.025	-0.044	0.012	6.6e-05	PRNCR1-CASC19
rs62429482	6	140254108	T/C	0.12	-0.026	0.006	6.6e-06	FILNC1-LOC100507477
rs9463551	6	9548285	G/A	0.059	0.034	0.008	6.6e-06	LOC100506207-TFAP2A
rs35169799	11	64031241	C/T	0.061	-0.038	0.008	6.6e-07	PLCB3
rs2725371	8	30854033	A/G	0.298	0.021	0.004	6.6e-07	PURG
rs943346	10	97254305	C/T	0.057	-0.043	0.008	6.6e-08	SORBS1
rs12030612	1	113215637	C/T	0.202	-0.025	0.005	6.6e-08	MOV10,CAPZA1,CAPZ

SNPid	CHR	pos	Effect/other alleles	Effect allele Freq	beta	SE	pval	closest gene
								A1-MOV10
rs113163918	16	66871701	C/T	0.044	-0.054	0.009	6.6e-09	NAE1-CA7
rs116337444	6	26208269	G/A	0.074	-0.052	0.007	6.6e-13	H4C5,H4C5-H2BC8
rs17456790	10	69872167	G/A	0.03	-0.043	0.011	6.7e-05	MYPN
rs2065524	1	3303731	G/A	0.083	0.026	0.007	6.7e-05	PRDM16
rs79273881	2	176866756	G/A	0.021	0.051	0.013	6.7e-05	LNPK
rs9862072	3	21551489	C/T	0.426	-0.015	0.004	6.7e-05	ZNF385D
rs150429489	3	49319336	C/T	0.099	-0.024	0.006	6.7e-05	C3orf62,USP4
rs17552503	5	73024386	T/G	0.221	0.018	0.005	6.7e-05	ARHGEF28
rs739797	7	117368587	G/A	0.186	-0.019	0.005	6.7e-05	CTTNBP2
rs143005749	19	49988353	G/A	0.021	-0.056	0.013	6.7e-06	RPL13A,SNORD32A,FLT3LG
rs145129276	1	110121058	C/A	0.105	-0.027	0.006	6.7e-06	GNAI3
rs8125763	20	17883531	C/A	0.468	-0.017	0.004	6.7e-06	BANF2-SNX5
rs13261451	8	112668862	T/C	0.435	-0.016	0.004	6.7e-06	LINC02237-CSMD3
rs4404888	8	77287466	C/T	0.409	-0.019	0.004	6.7e-07	HNF4G-LINC01111
rs778124	1	56606206	G/A	0.375	-0.024	0.004	6.7e-10	LINC01755-LINC01767
rs11607557	11	48099844	A/G	0.059	-0.053	0.008	6.7e-11	PTPRJ
rs7307248	12	96830278	C/T	0.245	-0.016	0.004	6.8e-05	CDK17-CFAP54
rs1876354	16	4919710	T/C	0.429	0.015	0.004	6.8e-05	UBN1
rs16837903	1	150552042	G/A	0.144	-0.02	0.005	6.8e-05	MCL1
rs17480516	22	28288532	G/A	0.072	0.029	0.007	6.8e-05	PITPNB
rs6009560	22	49629890	G/A	0.421	-0.015	0.004	6.8e-05	LINC01310-C22orf34
rs12475161	2	113823626	T/C	0.389	-0.015	0.004	6.8e-05	IL36RN,IL36RN-IL1F10
rs6728564	2	45947109	A/C	0.318	0.017	0.004	6.8e-05	PRKCE
rs1372579	7	52681177	G/A	0.406	0.015	0.004	6.8e-05	LOC107986794-POM121L12
rs12552768	9	126128960	G/A	0.398	0.016	0.004	6.8e-05	CRB2
rs10124866	9	24175234	T/C	0.219	0.018	0.005	6.8e-05	ELAVL2-IZUMO3
rs10814501	9	37019304	C/T	0.403	0.015	0.004	6.8e-05	PAX5
rs12780199	10	134553909	A/G	0.303	0.018	0.004	6.8e-06	INPP5A
rs74962019	16	66511905	C/T	0.036	-0.045	0.01	6.8e-06	BEAN1-AS1,BEAN1
rs79310937	22	29859506	C/T	0.127	-0.025	0.006	6.8e-06	RFPL1-NEFH
rs11586272	1	43844602	T/C	0.346	-0.021	0.004	6.8e-08	MED8,ELOVL1-MED8
rs72677847	14	50844256	T/C	0.033	0.059	0.01	6.8e-09	CDKL1
rs10457174	6	97067047	C/T	0.192	-0.028	0.005	6.8e-10	FHL5,FHL5-GPR63
rs75154776	13	50477325	T/C	0.145	-0.021	0.005	6.9e-05	CTAGE10P-SPRYD7
rs2240142	16	2812647	C/T	0.152	-0.021	0.005	6.9e-05	SRRM2
rs146207376	19	11477540	C/T	0.012	-0.071	0.018	6.9e-05	PLPPR2,PLPPR2-SWSAP1
rs72933784	1	80256101	T/C	0.188	0.019	0.005	6.9e-05	ADGRL4-LINC01781
rs114611189	2	159607955	C/T	0.037	0.039	0.01	6.9e-05	PKP4-AS1-DAPL1
rs4685517	3	2392718	T/G	0.488	0.015	0.004	6.9e-05	CNTN4
rs13126069	4	152212486	C/T	0.432	0.015	0.004	6.9e-05	PRSS48

SNPid	CHR	pos	Effect/other alleles	Effect allele Freq	beta	SE	pval	closest gene
rs4702056	5	14825589	T/C	0.274	-0.017	0.004	6.9e-05	MIR4637,ANKH
rs930527	17	6479655	T/C	0.408	-0.017	0.004	6.9e-06	KIAA0753,PITPNM3-KIAA0753
rs9424385	1	24887966	A/C	0.282	0.018	0.004	6.9e-06	NCMAP
rs10455695	6	70326403	A/G	0.219	0.021	0.005	6.9e-06	ADGRB3-LMBRD1
rs505492	9	82884212	C/T	0.172	0.021	0.005	6.9e-06	LINC01507-TLE1
rs6508055	18	48788446	G/A	0.22	-0.033	0.005	6.9e-14	MEX3C-LINC01630
rs1340362	10	73260244	G/A	0.386	0.016	0.004	7.1e-05	CDH23,CDH23
rs2293094	12	52316603	T/C	0.156	-0.02	0.005	7.1e-05	ACVRL1
rs80266153	13	85068465	A/G	0.03	-0.044	0.011	7.1e-05	LINC00333
rs8057913	16	2069167	T/C	0.32	-0.015	0.004	7.1e-05	NPW,ZNF598-NPW
rs4337311	16	7364117	A/G	0.173	0.019	0.005	7.1e-05	RBFOX1
rs13025932	2	181417009	T/C	0.183	-0.019	0.005	7.1e-05	CWC22-SCHLAP1
rs57886575	4	148424986	G/A	0.318	-0.017	0.004	7.1e-05	EDNRA
rs1118142	7	120648485	C/T	0.459	-0.015	0.004	7.1e-05	CPED1
rs141219399	8	76346309	G/A	0.02	-0.053	0.013	7.1e-05	HNF4G
rs10988229	9	131907510	T/C	0.209	0.017	0.005	7.1e-05	PTPA
rs4561126	10	95982601	C/T	0.35	0.017	0.004	7.1e-06	PLCE1
rs7961557	12	2493591	G/A	0.392	0.017	0.004	7.1e-06	CACNA1C
rs4968592	17	61612631	C/T	0.309	-0.019	0.004	7.1e-06	KCNH6
rs1354792	1	21391875	T/C	0.455	0.017	0.004	7.1e-06	EIF4G3
rs76611986	1	3220023	C/T	0.208	-0.02	0.005	7.1e-06	PRDM16
rs6953514	7	150724324	T/C	0.06	-0.036	0.008	7.1e-06	ATG9B,ABCB8,ATG9B-ABCB8
rs9326945	5	114425513	T/C	0.195	0.024	0.005	7.1e-07	LOC101927078-TRIM36
rs6947064	7	151397343	A/G	0.363	-0.019	0.004	7.1e-07	PRKAG2
rs56093896	2	114103966	C/A	0.211	0.024	0.005	7.1e-08	PAX8-CBWD2
rs34802409	3	53673715	G/A	0.192	0.025	0.005	7.1e-08	CACNA1D
rs4751828	10	123000544	G/A	0.204	-0.028	0.005	7.1e-09	WDR11-FGFR2
rs6547850	2	28629343	G/T	0.466	0.023	0.004	7.1e-10	FOSL2
rs7701003	5	157824481	A/G	0.375	0.048	0.004	7.1e-34	LINC02227
rs6760171	2	111754548	T/C	0.446	0.015	0.004	7.2e-05	ACOXL
rs17801458	2	142468472	C/T	0.061	0.029	0.008	7.2e-05	LRP1B
rs77846345	3	187701810	C/T	0.069	-0.029	0.007	7.2e-05	LINC01991-LPP-AS2
rs1735454	3	60678026	T/C	0.058	-0.031	0.008	7.2e-05	FHIT
rs1684174	7	131166993	A/G	0.467	-0.015	0.004	7.2e-05	MKLN1
rs292585	7	134884187	C/T	0.273	0.017	0.004	7.2e-05	WDR91
rs3977756	10	104398582	G/A	0.398	-0.017	0.004	7.2e-06	SUFU-TRIM8
rs62128715	19	10318222	A/G	0.26	-0.018	0.004	7.2e-06	DNMT1-S1PR2
rs1176387	1	117024491	G/T	0.38	0.017	0.004	7.2e-06	LINC01762,LINC01762-CD58
rs2275151	3	160003677	T/C	0.256	0.02	0.004	7.2e-06	IFT80,TRIM59-IFT80
rs73396126	6	25320087	C/T	0.213	-0.021	0.005	7.2e-06	CARMIL1
rs2239181	12	48255949	A/C	0.106	-0.03	0.006	7.2e-07	VDR

SNPid	CHR	pos	Effect/other alleles	Effect allele Freq	beta	SE	pval	closest gene
rs1899891	3	78628894	A/G	0.227	0.022	0.005	7.2e-07	LINC02077-ROBO1
rs7107727	11	10322019	C/T	0.08	0.036	0.007	7.2e-08	ADM,SBF2-ADM
rs17513752	19	30290811	A/G	0.322	-0.022	0.004	7.2e-08	C19orf12-CCNE1
rs6072407	20	40162802	G/A	0.331	0.02	0.004	7.2e-08	CHD6
rs13338270	16	19319549	T/G	0.206	-0.019	0.005	7.3e-05	CLEC19A
rs1357851	2	30613090	C/T	0.232	0.017	0.004	7.3e-05	LINC01936-LCLAT1
rs2244201	2	52552297	C/A	0.232	0.017	0.004	7.3e-05	LOC730100
rs58549997	4	11221366	C/T	0.034	-0.042	0.011	7.3e-05	CLNK-MIR572
rs4861036	4	42783873	A/C	0.101	-0.024	0.006	7.3e-05	ATP8A1-GRXCR1
rs392088	5	677718	G/A	0.366	0.015	0.004	7.3e-05	TPPP
rs17299478	16	69775500	C/T	0.161	-0.021	0.005	7.3e-06	NOB1,NQO1-NOB1
rs80105455	1	92661882	C/T	0.096	-0.027	0.006	7.3e-06	KIAA1107-C1orf146
rs4715296	6	52213658	T/G	0.247	0.02	0.004	7.3e-06	MCM3-PAQR8
rs7145048	14	39679917	A/C	0.192	0.025	0.005	7.3e-07	PNN-MIA2
rs1848864	12	115500103	A/G	0.32	0.023	0.004	7.3e-09	TBX3-MED13L
rs319681	3	47920240	C/T	0.301	0.024	0.004	7.3e-09	MAP4
rs10009490	4	81179173	A/G	0.104	0.047	0.006	7.3e-14	PRDM8-FGF5
rs28637873	14	24825677	C/T	0.081	0.028	0.007	7.4e-05	RIPK3-NFATC4
rs1996644	16	86086847	G/A	0.199	-0.018	0.005	7.4e-05	IRF8-LINC01082
rs10915993	1	226618382	A/C	0.334	-0.016	0.004	7.4e-05	PARP1-STUM
rs114584677	1	38011997	G/T	0.064	-0.03	0.008	7.4e-05	SNIP1
rs10205728	2	7416261	G/T	0.336	-0.015	0.004	7.4e-05	LOC101929452-LOC100506274
rs10477908	5	107876509	G/T	0.188	-0.018	0.005	7.4e-05	FBXL17-LINC01023
rs55736103	9	127969830	C/T	0.145	-0.021	0.005	7.4e-05	RABEPK
rs77662912	10	105368645	C/T	0.02	0.062	0.014	7.4e-06	SH3PXD2A
rs146816907	5	32876210	G/A	0.035	-0.045	0.01	7.4e-06	NPR3-LINC02120
rs56403239	8	113357973	A/G	0.465	0.017	0.004	7.4e-06	CSMD3
rs11006778	10	28228037	A/G	0.454	-0.02	0.004	7.4e-08	ARMC4
rs9328077	6	1979173	T/C	0.403	0.019	0.004	7.4e-08	GMDS
rs28418930	7	27472026	G/T	0.205	-0.026	0.005	7.4e-08	EVX1-HIBADH
rs71318356	3	183875419	C/T	0.02	-0.083	0.013	7.4e-10	DVL3
rs2680663	3	53735299	A/G	0.324	-0.031	0.004	7.4e-14	CACNA1D
rs6800730	3	48174210	A/G	0.337	-0.033	0.004	7.4e-16	MAP4-CDC25A
rs753992	11	47349846	G/A	0.178	0.048	0.005	7.4e-23	MYBPC3,MADD
rs10922552	1	89500792	G/T	0.096	-0.025	0.006	7.5e-05	GBP3-GBP1
rs6031456	20	42840019	A/G	0.141	-0.023	0.005	7.5e-05	OSER1,OSER1-DT
rs6752828	2	80187969	C/T	0.466	0.014	0.004	7.5e-05	CTNNA2
rs6804374	3	169343223	G/T	0.412	-0.015	0.004	7.5e-05	MECOM
rs13148238	4	143848478	G/A	0.138	0.021	0.005	7.5e-05	INPP4B-LOC105377623
rs28821226	5	18508285	C/T	0.198	0.019	0.005	7.5e-05	LINC02223-CDH18
rs766505	12	4875334	T/G	0.42	-0.017	0.004	7.5e-06	GALNT8

SNPid	CHR	pos	Effect/other alleles	Effect allele Freq	beta	SE	pval	closest gene
rs2389824	13	98014199	G/T	0.268	0.019	0.004	7.5e-06	MBNL2
rs72814595	5	157345934	C/T	0.218	0.021	0.005	7.5e-06	CLINT1-LINC02227
rs11087016	20	10997709	A/G	0.257	-0.027	0.004	7.5e-10	LOC101929413-C20orf187
rs1845501	5	32935090	T/C	0.296	-0.028	0.004	7.5e-12	NPR3-LINC02120
rs12921224	16	60449359	G/A	0.392	-0.015	0.004	7.6e-05	NPAP1L-MIR4426
rs9807152	18	46877219	G/A	0.174	0.02	0.005	7.6e-05	DYM
rs74692308	4	38456107	A/G	0.03	-0.043	0.011	7.6e-05	LINC01258
rs73018859	6	165354231	A/G	0.117	0.023	0.006	7.6e-05	MEAT6-C6orf118
rs9775587	9	122662720	G/T	0.403	-0.015	0.004	7.6e-05	BRINP1-LINC01613
rs7491248	13	47180671	G/A	0.22	-0.02	0.005	7.6e-06	LRCH1
rs13036715	20	47817815	G/A	0.234	0.02	0.004	7.6e-06	STAU1
rs1427298	2	145214421	C/T	0.421	0.016	0.004	7.6e-06	ZEB2
rs55902961	2	69691602	C/A	0.421	0.017	0.004	7.6e-06	AAK1
rs180883102	6	27747383	A/G	0.049	0.051	0.012	7.6e-06	LOC100131289-H2BC13
rs13116100	4	124814424	T/G	0.192	0.023	0.005	7.6e-07	LINC01091
rs17621806	17	46361051	C/T	0.029	-0.059	0.011	7.6e-08	SKAP1
rs35145102	2	174939083	G/A	0.248	-0.024	0.004	7.6e-08	OLA1
rs2365369	3	20117724	G/A	0.453	0.021	0.004	7.6e-09	KAT2B
rs1454651	21	45012476	A/G	0.403	-0.025	0.004	7.6e-11	HSF2BP
rs13265769	8	82824263	T/C	0.421	-0.024	0.004	7.6e-11	SNX16-LOC101927141
rs10994929	10	63582061	A/G	0.104	-0.024	0.006	7.7e-05	CABCOCO1-ARID5B
rs55646096	11	64918514	C/T	0.178	0.02	0.005	7.7e-05	SYVN1-SPDYC
rs17494309	12	27248979	A/G	0.123	-0.022	0.006	7.7e-05	C12orf71-STK38L
rs268822	14	57975646	G/A	0.201	-0.019	0.005	7.7e-05	CCDC198-SLC35F4
rs28363211	1	46724992	G/A	0.02	-0.051	0.013	7.7e-05	RAD54L
rs62285815	3	183782645	C/T	0.046	-0.033	0.009	7.7e-05	HTR3C,HTR3C-HTR3E-AS1
rs2643813	3	27537746	A/G	0.095	0.026	0.006	7.7e-05	SLC4A7-EOMES
rs243971	4	111287787	G/A	0.302	0.015	0.004	7.7e-05	ELOVL6-ENPEP
rs4551019	5	52672016	T/C	0.364	-0.015	0.004	7.7e-05	LOC257396-FST
rs139738489	7	129874985	G/A	0.023	0.05	0.012	7.7e-05	SSMEM1-CPA2
rs10093194	8	50441377	C/T	0.099	-0.025	0.006	7.7e-05	LOC100507464-SNTG1
rs11192323	10	106885045	A/G	0.139	-0.025	0.005	7.7e-06	SORCS3
rs12135871	1	171893880	A/G	0.214	-0.021	0.005	7.7e-06	DNM3
rs10084620	21	48049163	A/G	0.333	0.018	0.004	7.7e-06	S100B-PRMT2
rs57905605	6	150876184	T/G	0.033	0.048	0.01	7.7e-06	IYD-PLEKHG1
rs13154725	5	147894705	G/A	0.423	-0.019	0.004	7.7e-07	HTR4
rs306960	8	142005245	C/T	0.42	-0.018	0.004	7.7e-07	PTK2
rs7232858	18	773092	T/C	0.187	0.026	0.005	7.7e-08	YES1
rs647152	11	64109118	T/G	0.376	0.015	0.004	7.8e-05	CCDC88B,MIR7155
rs74803604	14	64654787	T/C	0.073	-0.027	0.007	7.8e-05	SYNE2
rs72677920	1	62470234	T/G	0.189	0.019	0.005	7.8e-05	PATJ

SNPid	CHR	pos	Effect/other alleles	Effect allele Freq	beta	SE	pval	closest gene
rs622721	1	71413324	C/T	0.382	0.014	0.004	7.8e-05	PTGER3,PTGER3
rs72793611	2	53857526	C/T	0.192	-0.019	0.005	7.8e-05	MIR4431-ASB3
rs16872363	5	3740531	T/C	0.444	0.015	0.004	7.8e-05	IRX1-LINC02114
rs116306020	6	30962678	G/A	0.039	-0.041	0.01	7.8e-05	MUC21,MUC21-MUC22
rs6960452	7	69611407	C/T	0.013	-0.066	0.017	7.8e-05	AUTS2
rs2026811	9	101879263	C/A	0.218	-0.018	0.005	7.8e-05	TGFBR1
rs17224476	11	4673788	G/A	0.111	-0.027	0.006	7.8e-06	OR51E1
rs1959440	14	30208630	G/T	0.442	-0.018	0.004	7.8e-06	PRKD1
rs72910629	6	69761994	A/G	0.136	-0.024	0.006	7.8e-06	ADGRB3
rs601551	11	77554821	G/A	0.386	-0.019	0.004	7.8e-07	AAMDC
rs2046850	1	210304319	C/T	0.188	0.024	0.005	7.8e-07	SYT14
rs12124523	1	72621463	C/T	0.103	-0.03	0.006	7.8e-07	NEGR1
rs2186797	11	70007770	T/C	0.064	-0.039	0.008	7.8e-08	ANO1
rs2941732	10	22231670	A/G	0.271	0.017	0.004	7.9e-05	DNAJC1
rs4762758	12	20614659	G/A	0.443	0.016	0.004	7.9e-05	PDE3A
rs2736609	1	156202640	C/T	0.357	0.015	0.004	7.9e-05	PMF1,PMF1-BGLAP
rs73188943	22	50815104	C/T	0.234	-0.018	0.004	7.9e-05	PPP6R2
rs142258694	3	167228461	T/C	0.031	-0.043	0.011	7.9e-05	WDR49
rs79095744	4	19475854	C/T	0.048	0.035	0.009	7.9e-05	LCORL-SLIT2
rs607342	6	139830218	A/C	0.412	0.015	0.004	7.9e-05	LINC01625-FILNC1
rs2080349	7	36350349	G/A	0.188	-0.018	0.005	7.9e-05	EEPD1-KIAA0895
rs2402948	13	115093704	C/T	0.21	-0.02	0.005	7.9e-06	CHAMP1,LINC01054,CHAMP1-LINC01054
rs145486593	18	51692749	C/T	0.017	0.062	0.014	7.9e-06	MBD2
rs7440077	4	3792442	T/C	0.421	0.017	0.004	7.9e-06	ADRA2C-FAM86EP
rs4895588	6	141497409	G/A	0.265	-0.02	0.004	7.9e-06	MIR4465-NMBR
rs848309	1	16308447	T/C	0.425	-0.022	0.004	7.9e-09	ZBTB17-SRARP
rs875106	11	70005641	G/A	0.486	0.015	0.004	8.1e-05	ANO1
rs4465386	11	81387764	C/A	0.291	0.016	0.004	8.1e-05	LINC02720-MIR4300HG
rs61944572	12	125356471	A/G	0.069	0.029	0.007	8.1e-05	SCARB1-UBC
rs7150826	14	60771653	T/C	0.256	0.017	0.004	8.1e-05	PPM1A-LINC02322
rs11900605	2	114449152	C/T	0.401	-0.015	0.004	8.1e-05	RABL2A-SLC35F5
rs56183833	2	161748439	G/A	0.204	-0.018	0.005	8.1e-05	RBMS1-TANK
rs7737906	5	153012186	C/T	0.28	0.016	0.004	8.1e-05	GRIA1
rs244545	5	50928871	A/G	0.369	0.015	0.004	8.1e-05	ISL1-LINC02118
rs76077988	1	89118331	G/A	0.017	0.067	0.015	8.1e-06	PKN2-AS1
rs4691377	4	157694651	C/T	0.144	0.024	0.005	8.1e-06	PDGFC
rs11741847	5	68332072	G/T	0.282	0.02	0.004	8.1e-07	LINC02198-SLC30A5
rs7216547	17	47509463	A/C	0.411	-0.02	0.004	8.1e-08	PHB-LINC02075
rs6842303	4	17854055	T/G	0.266	-0.023	0.004	8.1e-08	LCORL
rs17167306	7	133529648	A/C	0.138	0.031	0.005	8.1e-09	EXOC4
rs151545	10	115700005	C/A	0.112	0.038	0.006	8.1e-11	NHLRC2-ADRB1

SNPid	CHR	pos	Effect/other alleles	Effect allele Freq	beta	SE	pval	closest gene
rs3799354	6	25830038	C/A	0.114	-0.049	0.006	8.1e-17	SLC17A1
rs74971367	11	6372010	A/G	0.089	-0.027	0.007	8.2e-05	CAVIN3-SMPD1
rs78380887	18	35838871	G/A	0.048	-0.033	0.009	8.2e-05	MIR4318-MIR924HG
rs2357440	4	148053679	G/A	0.122	-0.023	0.006	8.2e-05	TTC29-MIR548G
rs6450394	5	55875713	T/C	0.276	0.016	0.004	8.2e-05	C5orf67
rs35143662	6	97414949	C/T	0.036	-0.04	0.01	8.2e-05	KLHL32
rs116537710	1	151044947	C/T	0.044	-0.038	0.009	8.2e-06	MLLT11,GABPB2
rs4958011	5	111531058	T/C	0.08	0.03	0.007	8.2e-06	EPB41L4A
rs11688048	2	24807533	C/T	0.098	-0.03	0.006	8.2e-07	NCOA1
rs58203769	5	148618708	A/G	0.466	-0.019	0.004	8.2e-07	ABLIM3
rs1046872	11	111179390	A/G	0.358	0.021	0.004	8.2e-08	COLCA2,COLCA1
rs9321410	6	133998133	T/C	0.047	0.046	0.009	8.2e-08	TARID
rs301799	1	8489302	C/T	0.417	-0.025	0.004	8.2e-11	RERE,RERE-AS1
rs72854502	11	9951967	A/C	0.132	-0.04	0.006	8.2e-14	LOC101928008,SBF2
rs3752962	17	1939543	T/C	0.314	0.016	0.004	8.3e-05	DPH1
rs2836478	21	39906374	T/G	0.167	0.02	0.005	8.3e-05	ERG
rs34776824	2	153799994	G/A	0.25	0.017	0.004	8.3e-05	ARL6IP6-RPRM
rs41313071	7	150642752	C/A	0.042	0.035	0.009	8.3e-05	KCNH2
rs7033474	9	37482333	C/T	0.407	-0.015	0.004	8.3e-05	POLR1E,ZBTB5-POLR1E
rs113467371	9	95330812	G/A	0.012	0.068	0.017	8.3e-05	CENPP
rs72944265	11	81437126	C/A	0.145	-0.025	0.005	8.3e-06	LINC02720-MIR4300HG
rs146336654	12	49305999	G/A	0.033	-0.045	0.01	8.3e-06	CCDC65
rs2423515	20	10703993	A/G	0.131	0.025	0.006	8.3e-06	JAG1-LINC01752
rs12521166	5	3686190	A/G	0.075	-0.032	0.007	8.3e-06	IRX1-LINC02114
rs248686	5	60354522	C/T	0.251	-0.018	0.004	8.3e-06	NDUFAF2
rs75359244	12	69732379	G/T	0.041	-0.047	0.009	8.3e-07	CPSF6-LYZ
rs1982468	19	4953351	T/G	0.361	0.021	0.004	8.3e-08	UHRF1
rs2125855	1	204546947	T/C	0.271	-0.023	0.004	8.3e-08	MDM4-LRRN2
rs1918898	2	188100639	C/T	0.357	0.021	0.004	8.3e-08	ZSWIM2-CALCRL
rs6958142	7	94112882	G/T	0.288	0.022	0.004	8.3e-08	COL1A2-CASD1
rs72838866	6	25673273	A/G	0.13	-0.04	0.006	8.3e-12	SCGN
rs4820847	22	30810945	G/A	0.298	-0.017	0.004	8.4e-05	SEC14L2
rs10045403	5	96147733	A/G	0.266	-0.017	0.004	8.4e-05	ERAP1,ERAP1
rs2475068	6	26827896	T/C	0.361	0.017	0.005	8.4e-05	ZNF322-GUSBP2
rs62406248	6	55657201	A/G	0.071	0.028	0.007	8.4e-05	BMP5
rs7297116	12	116015954	G/A	0.251	0.019	0.004	8.4e-06	TBX3-MED13L
rs36181536	12	122528263	T/C	0.322	0.017	0.004	8.4e-06	MLXIP
rs11752783	6	43347305	G/A	0.103	-0.028	0.006	8.4e-06	ZNF318-ABCC10
rs7765042	6	131141444	C/T	0.38	-0.019	0.004	8.4e-07	TMEM200A-SMLR1
rs134564	22	29461790	T/C	0.294	-0.022	0.004	8.4e-08	C22orf31,C22orf31-KREMEN1
rs1948505	4	15020226	A/G	0.347	0.021	0.004	8.4e-08	CPEB2,C1QTNF7-AS1

SNPid	CHR	pos	Effect/other alleles	Effect allele Freq	beta	SE	pval	closest gene
rs12237112	9	98934208	C/T	0.197	0.026	0.005	8.4e-08	LOC158434-HSD17B3
rs4283241	16	29988349	G/A	0.456	-0.021	0.004	8.4e-09	TMEM219,TAOK2
rs3957698	11	95453615	G/A	0.499	0.014	0.004	8.5e-05	LOC100129203-FAM76B
rs72829869	17	46662822	G/A	0.076	-0.028	0.007	8.5e-05	HOXB-AS3,HOXB3-HOXB-AS3
rs11664045	18	42220367	C/T	0.231	-0.017	0.004	8.5e-05	LINC01601-SETBP1
rs138938221	1	46683480	G/A	0.06	0.03	0.008	8.5e-05	POMGNT1,LURAP1
rs17881233	6	31323745	C/A	0.019	0.076	0.02	8.5e-05	MIR6891,HLA-B
rs2504706	6	51258579	T/C	0.232	0.017	0.004	8.5e-05	TFAP2B-PKHD1
rs6958617	7	39274454	T/C	0.361	-0.015	0.004	8.5e-05	POU6F2
rs10957090	8	60275254	G/A	0.162	0.019	0.005	8.5e-05	LOC100505501-CA8
rs74528956	1	94086067	G/T	0.022	0.055	0.013	8.5e-06	BCAR3
rs4665135	2	160872397	T/C	0.167	-0.022	0.005	8.5e-06	PLA2R1
rs920271	2	23620246	T/C	0.24	0.02	0.004	8.5e-06	KLHL29
rs16939764	12	110358336	G/A	0.036	0.051	0.01	8.5e-07	TCHP,TCHP-GIT2
rs2735357	10	89662504	A/G	0.252	-0.024	0.004	8.5e-08	PTEN
rs460879	16	89712889	C/T	0.426	0.033	0.004	8.5e-19	CHMP1A
rs77890156	11	10651842	T/C	0.067	0.03	0.008	8.6e-05	MRVI1
rs80118038	11	1880608	G/A	0.019	0.056	0.014	8.6e-05	MIR4298,LSP1
rs614468	17	75261986	C/T	0.264	0.016	0.004	8.6e-05	SEC14L1-SEPTIN9-DT
rs3730714	18	51802708	C/A	0.402	-0.015	0.004	8.6e-05	POLI
rs115438739	1	894519	G/A	0.022	0.05	0.013	8.6e-05	KLHL17,NOC2L
rs4809606	20	45579789	G/A	0.438	-0.015	0.004	8.6e-05	EYA2
rs17582253	2	184988375	C/T	0.463	-0.015	0.004	8.6e-05	NUP35-MIR548AE1
rs62289651	3	181016025	T/C	0.147	0.021	0.005	8.6e-05	SOX2-OT
rs4647512	9	97888730	C/T	0.056	-0.032	0.008	8.6e-05	FANCC
rs12624791	20	10842445	C/T	0.192	-0.025	0.005	8.6e-08	LINC01752-LOC101929413
rs12302980	12	111360290	A/G	0.408	0.022	0.004	8.6e-09	MYL2,MYL2-LINC01405
rs6692465	1	163231003	A/G	0.302	0.023	0.004	8.6e-09	RGS5
rs77867281	17	58989120	G/T	0.145	-0.021	0.005	8.7e-05	BCAS3
rs4891261	18	73017142	C/T	0.35	-0.015	0.004	8.7e-05	TSHZ1-SMIM21
rs9431947	1	231302356	T/C	0.108	0.024	0.006	8.7e-05	TRIM67
rs150305200	2	73412769	C/A	0.019	-0.055	0.013	8.7e-05	RAB11FIP5-NOTO
rs3932940	5	202282	C/T	0.365	0.016	0.004	8.7e-05	CCDC127
rs13179942	5	37279482	C/T	0.315	0.015	0.004	8.7e-05	LOC105374727-NUP155
rs1421361	7	131459782	A/C	0.405	0.015	0.004	8.7e-05	PODXL-LOC101928782
rs10748526	10	82273079	T/C	0.204	0.02	0.005	8.7e-06	TSPAN14
rs115595248	5	158035146	A/C	0.044	0.039	0.009	8.7e-06	LINC02227-EBF1
rs11832207	12	89627782	C/T	0.134	-0.027	0.005	8.7e-07	LINC02458-DUSP6
rs12193642	6	133200185	G/A	0.082	-0.034	0.007	8.7e-07	RPS12-LINC00326
rs11573607	9	110045257	T/C	0.122	0.029	0.006	8.7e-07	RAD23B,LOC340512-RAD23B

SNPid	CHR	pos	Effect/other alleles	Effect allele Freq	beta	SE	pval	closest gene
rs10757739	9	28414915	A/G	0.452	-0.019	0.004	8.7e-07	LINGO2
rs76558307	6	26448824	C/A	0.039	-0.052	0.01	8.7e-08	BTN3A3
rs16839055	3	132009881	C/T	0.283	-0.024	0.004	8.7e-09	CPNE4-ACP3
rs741066	7	150500520	C/T	0.294	-0.026	0.004	8.7e-10	TMEM176A,TMEM176B
rs73563812	8	25900405	G/T	0.236	0.041	0.004	8.7e-21	EBF2
rs1164760	12	105063254	C/T	0.264	0.015	0.004	8.8e-05	CHST11
rs3815129	12	48204456	T/C	0.17	-0.019	0.005	8.8e-05	HDAC7
rs1461213	15	88520291	A/G	0.394	0.015	0.004	8.8e-05	NTRK3
rs1786059	18	34803070	G/A	0.303	0.016	0.004	8.8e-05	KIAA1328
rs17358345	1	163269956	A/G	0.17	-0.019	0.005	8.8e-05	RGS5,RGS5
rs2235394	6	11763351	T/G	0.374	0.015	0.004	8.8e-05	ADTRP
rs7014583	8	142263393	T/C	0.356	-0.015	0.004	8.8e-05	LOC105375787,SLC45A4
rs2284704	14	61211868	T/C	0.261	0.019	0.004	8.8e-06	MNAT1
rs3853476	5	141817754	G/A	0.367	0.017	0.004	8.8e-06	SPRY4-AS1
rs2980872	8	126416920	G/A	0.476	0.017	0.004	8.8e-06	NSMCE2-TRIB1
rs1812736	8	76299138	G/A	0.173	-0.022	0.005	8.8e-06	CASC9-HNF4G
rs2934849	6	166162335	T/C	0.362	0.024	0.004	8.8e-10	PDE10A-LINC00473
rs145054300	11	46341259	G/A	0.114	-0.043	0.006	8.8e-13	CREB3L1
rs75673411	11	50120874	A/G	0.131	0.041	0.006	8.8e-14	OR4C12-LOC441601
rs12572674	10	107733983	T/G	0.22	-0.017	0.005	8.9e-05	LINC02627-LINC02624
rs10129307	14	71178861	T/C	0.124	0.022	0.006	8.9e-05	LINC01269
rs17606460	17	28899024	C/T	0.228	-0.018	0.005	8.9e-05	SMURF2P1-LRRC37BP1,GOSR1-SMURF2P1-LRRC37BP1
rs6518682	22	30612949	A/G	0.445	-0.016	0.004	8.9e-05	HORMAD2-LIF-AS1
rs73166732	22	28257368	C/A	0.112	-0.027	0.006	8.9e-06	PITPNB
rs6088864	20	30228157	G/A	0.195	0.023	0.005	8.9e-07	COX4I2
rs6747797	2	181984486	A/G	0.293	-0.02	0.004	8.9e-07	LINC01934,UBE2E3-LINC01934
rs76611746	6	50379067	G/A	0.051	-0.042	0.008	8.9e-07	DEFB112-TFAP2D
rs10281368	7	99655897	A/G	0.283	-0.021	0.004	8.9e-07	ZSCAN21
rs1034565	22	19984211	C/T	0.281	-0.023	0.004	8.9e-09	ARVCF
rs27433	5	96109935	T/G	0.153	-0.033	0.005	8.9e-10	CAST,ERAP1
rs11255578	10	8250682	T/C	0.348	-0.015	0.004	9.1e-05	GATA3-LINC00708
rs10844237	12	32701877	A/G	0.317	-0.016	0.004	9.1e-05	FGD4
rs76154667	12	50960228	A/G	0.042	0.036	0.009	9.1e-05	DIP2B
rs11620170	13	97653092	G/A	0.452	-0.015	0.004	9.1e-05	OXGR1-LINC00456
rs4784323	16	53797565	A/G	0.32	-0.017	0.004	9.1e-05	FTO
rs2199433	16	82816208	A/G	0.157	-0.02	0.005	9.1e-05	CDH13,LOC101928446
rs3766612	1	210020013	C/A	0.203	0.019	0.005	9.1e-05	UTP25
rs6019321	20	47240081	T/G	0.055	0.03	0.008	9.1e-05	PREX1,LINC00494-PREX1
rs3846947	6	15066724	T/C	0.444	-0.016	0.004	9.1e-05	LINC01108-JARID2

SNPid	CHR	pos	Effect/other alleles	Effect allele Freq	beta	SE	pval	closest gene
rs1571644	9	112351266	C/T	0.14	-0.022	0.005	9.1e-05	MIR3927-PALM2AKAP2
rs884181	10	70586459	T/C	0.129	-0.027	0.006	9.1e-07	STOX1,CCAR1-STOX1
rs283621	5	73197865	A/C	0.443	0.019	0.004	9.1e-07	ARHGEF28
rs7829839	8	105943563	G/A	0.439	0.018	0.004	9.1e-07	LRP12-ZFPM2
rs17517959	1	221346974	T/C	0.377	-0.026	0.004	9.1e-12	HLX-C1orf140
rs9933403	16	1850194	C/T	0.135	-0.022	0.005	9.2e-05	IGFALS-HAGH
rs2821557	1	111218920	C/T	0.448	-0.015	0.004	9.2e-05	KCNA3,KCNA3-CD53
rs1822881	2	146044052	A/G	0.314	-0.016	0.004	9.2e-05	TEX41-PABPC1P2
rs6765810	3	133778591	G/A	0.353	-0.015	0.004	9.2e-05	LINC02000,LINC02000-RYK
rs1035699	11	19756763	G/A	0.496	-0.016	0.004	9.2e-06	NAV2
rs4900592	14	104177098	G/T	0.32	-0.02	0.004	9.2e-07	ZFYVE21,XRCC3
rs10199186	2	28662375	C/T	0.072	-0.035	0.007	9.2e-07	FOSL2-PLB1
rs7633280	3	168581491	A/G	0.371	-0.019	0.004	9.2e-07	EGFEM1P-LINC02082
rs61894512	11	76178672	G/T	0.283	-0.022	0.004	9.2e-08	EMSY
rs76594121	3	128189391	T/G	0.041	0.052	0.009	9.2e-09	DNAJB8,DNAJB8-AS1
rs7810507	7	130437476	G/A	0.28	-0.025	0.004	9.2e-10	KLF14-MIR29A
rs12985940	19	7262734	T/C	0.165	0.055	0.005	9.2e-27	INSR
rs12866098	13	73119617	G/A	0.338	-0.016	0.004	9.3e-05	DACH1-MZT1
rs114185007	15	47563970	A/G	0.031	-0.041	0.011	9.3e-05	SEMA6D
rs143860466	1	163141900	T/C	0.021	0.048	0.012	9.3e-05	RGS5,LOC101928404
rs8115500	20	44121807	G/A	0.124	-0.021	0.006	9.3e-05	WFDC2-SPINT3
rs115796127	2	206889502	C/T	0.076	-0.028	0.007	9.3e-05	INO80D
rs2678481	2	233324491	A/C	0.027	-0.045	0.011	9.3e-05	ALPI
rs906592	3	62108240	A/G	0.355	0.015	0.004	9.3e-05	PTPRG
rs10248539	7	17103753	A/C	0.409	0.015	0.004	9.3e-05	AGR3-AHR
rs7155504	14	36158828	T/C	0.084	0.027	0.007	9.3e-06	RALGAPA1
rs72758941	15	64672082	C/T	0.06	-0.038	0.008	9.3e-06	PCLAF
rs4969531	20	61161246	A/G	0.374	0.017	0.004	9.3e-06	MIR133A2,MIR1-1HG
rs2005613	2	96423982	C/T	0.22	-0.02	0.005	9.3e-06	TRIM43-LINC00342
rs9847186	3	25081857	G/A	0.43	0.017	0.004	9.3e-06	RARB
rs16873941	7	130953631	T/C	0.064	0.036	0.008	9.3e-06	MKLN1
rs7213608	17	21279289	C/T	0.317	0.019	0.004	9.3e-07	KCNJ12,MAP2K3-KCNJ12
rs2821248	1	72575560	A/G	0.172	0.024	0.005	9.3e-07	NEGR1
rs6017308	20	42899340	G/A	0.192	0.023	0.005	9.3e-07	GDAP1L1
rs7781606	7	150496528	C/T	0.171	0.024	0.005	9.3e-07	TMEM176A,TMEM176B
rs9549617	13	113628070	C/A	0.252	-0.016	0.004	9.4e-05	MCF2L
rs4646316	22	19952132	C/T	0.245	0.017	0.004	9.4e-05	MIR4761,COMT
rs2123478	4	41290052	C/T	0.39	0.015	0.004	9.4e-05	UCHL1-LIMCH1
rs12205238	6	154872954	C/T	0.126	-0.023	0.006	9.4e-05	CNKSR3-SCAF8
rs76683802	10	14336554	G/A	0.03	-0.048	0.011	9.4e-06	FRMD4A,FRMD4A

SNPid	CHR	pos	Effect/other alleles	Effect allele Freq	beta	SE	pval	closest gene
rs17191323	15	60576262	G/A	0.152	0.023	0.005	9.4e-06	FOXB1-ANXA2
rs1561103	17	75321964	T/G	0.229	0.019	0.004	9.4e-06	SEPTIN9
rs10409238	19	21888264	T/C	0.349	0.018	0.004	9.4e-06	LOC400682-ZNF100
rs1361697	6	22568534	T/G	0.234	-0.02	0.004	9.4e-06	HDGFL1,PRL-HDGFL1
rs11070245	15	40317792	T/G	0.476	-0.019	0.004	9.4e-07	EIF2AK4
rs938662	2	45864578	A/G	0.283	0.02	0.004	9.4e-07	SRBD1-PRKCE
rs1075440	16	53790906	G/A	0.318	-0.022	0.004	9.4e-08	FTO
rs11996715	8	141647291	C/A	0.494	0.02	0.004	9.4e-08	AGO2,AGO2-PTK2
rs6124629	20	42804988	G/A	0.452	-0.024	0.004	9.4e-11	JPH2
rs10500326	16	4918326	G/T	0.234	0.035	0.004	9.4e-16	UBN1
rs10832728	11	17070581	A/G	0.469	-0.014	0.004	9.5e-05	OR7E14P,PLEKHA7-OR7E14P
rs9521729	13	111029970	G/A	0.313	0.015	0.004	9.5e-05	COL4A2
rs138413	22	38860071	C/T	0.257	-0.016	0.004	9.5e-05	KDELR3,KCNJ4-KDELR3
rs775761	3	77587102	G/A	0.288	0.016	0.004	9.5e-05	ROBO2
rs11241963	5	127906367	A/G	0.135	-0.023	0.006	9.5e-05	FBN2-SLC27A6
rs4524734	7	134382784	C/T	0.347	0.015	0.004	9.5e-05	BPGM-CALD1
rs2948300	8	8106508	C/T	0.454	0.025	0.006	9.5e-05	FAM86B3P-PRAG1
rs325952	1	96819562	A/C	0.183	-0.021	0.005	9.5e-06	LINC01787
rs72874197	6	62026409	A/C	0.222	0.02	0.005	9.5e-06	LINC00680-GUSBP4-KHDRBS2
rs2940584	5	61454880	C/T	0.135	0.027	0.005	9.5e-07	C5orf64-AS1-KIF2A
rs1077015	15	101901273	G/T	0.298	-0.016	0.004	9.6e-05	PCSK6
rs6056043	20	8705496	A/C	0.089	-0.026	0.006	9.6e-05	PLCB1
rs11677670	2	25510276	C/T	0.179	0.019	0.005	9.6e-05	DNMT3A
rs2121152	5	142852051	G/T	0.294	-0.016	0.004	9.6e-05	NR3C1-MIR5197
rs76068602	6	128813594	A/G	0.018	0.053	0.014	9.6e-05	PTPRK
rs304933	18	77164443	A/G	0.244	0.02	0.004	9.6e-06	NFATC1
rs2764839	1	42368704	A/G	0.358	0.016	0.004	9.6e-06	HIVEP3
rs5745437	1	76333352	G/A	0.074	0.031	0.007	9.6e-06	MSH4
rs6040305	20	11003605	G/T	0.059	0.033	0.008	9.6e-06	C20orf187,LOC101929413-C20orf187
rs7900405	10	18765673	A/G	0.392	0.018	0.004	9.6e-07	CACNB2
rs3794730	17	28150359	A/G	0.334	-0.02	0.004	9.6e-07	SSH2
rs6011292	20	62724698	C/T	0.226	-0.023	0.005	9.6e-07	OPRL1
rs6445819	3	56753662	C/T	0.324	0.021	0.004	9.6e-08	TASOR-ARHGEF3
rs12418343	11	55687400	C/A	0.127	0.041	0.006	9.6e-14	OR5W2-OR5I1
rs1981405	11	77976208	C/T	0.12	0.023	0.006	9.7e-05	GAB2
rs1677173	12	31751601	G/A	0.28	-0.016	0.004	9.7e-05	DENND5B-AS1
rs12812760	12	90093194	G/A	0.061	-0.028	0.008	9.7e-05	ATP2B1
rs8069010	17	20989265	T/C	0.168	-0.02	0.005	9.7e-05	LINC01563
rs17056773	18	72988700	T/G	0.181	0.018	0.005	9.7e-05	TSHZ1
rs2244665	20	54153759	A/G	0.343	-0.015	0.004	9.7e-05	LINC01441-CBLN4

SNPid	CHR	pos	Effect/other alleles	Effect allele Freq	beta	SE	pval	closest gene
rs1464907	7	17199435	A/G	0.384	-0.015	0.004	9.7e-05	AGR3-AHR
rs4512901	12	54365236	A/C	0.336	-0.018	0.004	9.7e-06	HOXC11,HOTAIR
rs1268123	6	126343258	C/T	0.16	-0.023	0.005	9.7e-06	TRMT11
rs6462008	7	27349479	G/T	0.372	-0.017	0.004	9.7e-06	EVX1-HIBADH
rs62460739	7	77406747	G/A	0.272	0.018	0.004	9.7e-06	RSBN1L
rs13072556	3	160401027	C/A	0.263	0.022	0.004	9.7e-07	ARL14,ARL14-PPM1L
rs11740542	5	158191768	A/G	0.18	0.027	0.005	9.7e-09	EBF1
rs10941022	5	32691416	C/T	0.36	0.044	0.004	9.7e-29	NPR3
rs1693628	10	126837986	G/A	0.15	-0.019	0.005	9.8e-05	CTBP2
rs4301837	12	102336310	T/C	0.488	-0.015	0.004	9.8e-05	DRAM1-WASHC3
rs1764390	1	35259961	A/G	0.363	0.015	0.004	9.8e-05	GJA4
rs10155342	4	20959274	C/T	0.413	0.014	0.004	9.8e-05	KCNIP4
rs4731776	7	130949410	G/T	0.491	-0.015	0.004	9.8e-05	MKLN1
rs59352280	17	7946297	T/G	0.068	-0.034	0.008	9.8e-06	ALOX15B
rs151356	20	57607949	G/A	0.196	0.029	0.005	9.8e-10	ATP5F1E,PRELID3B,SLMO2-ATP5E
rs10792315	11	61274790	T/G	0.09	0.046	0.007	9.8e-12	MIR4488,LRRC10B,PPP1R32-MIR4488
rs7919338	10	127731365	G/A	0.27	-0.016	0.004	9.9e-05	ADAM12
rs1339945	10	72553426	T/C	0.035	0.041	0.01	9.9e-05	TBATA-SGPL1
rs73205605	12	112225886	G/A	0.043	-0.034	0.009	9.9e-05	ALDH2
rs28759351	15	80352566	T/C	0.326	-0.015	0.004	9.9e-05	ZFAND6
rs8113341	19	10165932	A/G	0.212	-0.019	0.005	9.9e-05	C3P1
rs112146197	19	53606958	A/C	0.077	-0.026	0.007	9.9e-05	ZNF160,ZNF415,ZNF160-ZNF415
rs12151707	2	175330938	T/C	0.206	0.018	0.005	9.9e-05	GPR155
rs1468722	5	148295679	C/A	0.477	0.015	0.004	9.9e-05	ADRB2-SH3TC2
rs998509	5	96132795	G/A	0.129	-0.023	0.006	9.9e-05	ERAP1
rs3784924	16	11644842	A/G	0.315	-0.018	0.004	9.9e-06	LITAF
rs75420517	3	154605196	G/A	0.099	0.028	0.006	9.9e-06	GPR149-MME
rs10857216	4	83929495	A/G	0.263	0.02	0.004	9.9e-06	LIN54
rs12897688	14	100844420	G/A	0.222	0.022	0.005	9.9e-07	WARS1,WDR25
rs80157655	5	107198257	T/C	0.178	-0.023	0.005	9.9e-07	FBXL17
rs1451506	17	57407019	A/G	0.111	0.033	0.006	9.9e-09	YPEL2,GDPD1-YPEL2
rs3867466	11	111668256	A/C	0.278	-0.029	0.004	9.9e-13	ALG9
rs12371292	12	90250993	C/T	0.263	-0.043	0.004	9.9e-24	ATP2B1-AS1-LINC02399

Table S1. List of 2,166 clumped variants of the best fitting model. The statistics are based on the hypertension GWAS performed on UK Biobank dataset including 144,793 cases and 313,761 controls. SNPid, rs code of single nucleotide variant; CHR, chromosome; pos, base pairs position; Effect allele, the allele to which the effect estimate refers; Other allele, baseline allele; Effect allele freq, effect allele frequency; beta, effect estimate; SE, standard error; pval, p-value; closest gene, the gene mapping the nearest region.

		Target			Validation		
KEGG Set	Num SNP	Empirical P Enrichment	P_{FDR}	Zscore	Empirical P Enrichment	P_{FDR}	Zscore
CALCIUM SIGNALING PATHWAY	1669	0.003	0.001*	4.402	0.074	0.001*	4.323
INOSITOL PHOSPHATE METABOLISM	439	0.006	0.01*	3.446	0.096	0.035*	2.945
PHOSPHATIDYLINOSITOL SIGNALING SYSTEM	758	0.011	0.01*	3.429	0.136	0.027*	3.096
FOCAL ADHESION	1575	0.029	0.01*	3.541	0.102	0.014*	3.463
VEGF SIGNALING PATHWAY	439	0.012	0.021*	3.169	0.009	0.004*	3.957
MTOR SIGNALING PATHWAY	278	0.022	0.046*	2.783	0.053	0.033*	2.945
HUNTINGTONS DISEASE	799	0.042	0.037*	2.907	0.061	0.027*	3.124
SYSTEMIC LUPUS ERYTHEMATOSUS	298	0.020	0.046*	2.796	0.186	0.309	1.607
VALINE LEUCINE AND ISOLEUCINE BIOSYNTHESIS	47	0.007	0.039*	2.862	0.998	0.997	-0.004
ERBB SIGNALING PATHWAY	760	0.040	0.037*	2.897	0.339	0.293	1.655
RENAL CELL CARCINOMA	1221	0.019	0.031*	2.985	0.833	0.854	-0.363
RNA DEGRADATION	218	0.009	0.027*	3.048	0.529	0.662	0.752
TOLL LIKE RECEPTOR SIGNALING PATHWAY	437	0.013	0.021*	3.134	0.532	0.629	0.899
RIBOSOME	178	0.007	0.021*	3.165	0.309	0.392	1.419
COLORECTAL CANCER	450	0.010	0.019*	3.240	0.755	0.715	0.657
PANCREATIC CANCER	421	0.005	0.01*	3.500	0.544	0.515	1.100
VASCULAR SMOOTH MUSCLE CONTRACTION	1102	0.018	0.01*	3.527	0.761	0.438	1.288
INSULIN SIGNALING PATHWAY	726	0.006	0.009*	3.650	0.636	0.490	1.169
CHRONIC MYELOID LEUKEMIA	512	0.003	0.009*	3.710	0.668	0.451	1.256
PATHWAYS IN CANCER	2366	0.049	0.009*	3.624	0.908	0.419	1.361
MAPK SIGNALING PATHWAY	1718	0.008	0.002*	4.164	0.808	0.302	1.629
AMYOTROPHIC LATERAL SCLEROSIS ALS	314	0.974	0.976	-0.037	0.010	0.014*	3.486
COMPLEMENT AND COAGULATION CASCADES	337	0.265	0.365	1.432	0.024	0.044*	2.858
CIRCADIAN RHYTHM MAMMAL	79	0.213	0.401	1.347	0.025	0.035*	2.950
TRYPTOPHAN METABOLISM	184	0.656	0.766	0.509	0.017	0.032*	3.017
LEUKOCYTE TRANSENDOTHELIAL MIGRATION	888	0.284	0.218	1.775	0.122	0.027*	3.081
LONG TERM DEPRESSION	755	0.242	0.206	1.846	0.069	0.027*	3.111
GAP JUNCTION	776	0.184	0.151	2.058	0.081	0.027*	3.119
LONG TERM POTENTIATION	685	0.154	0.145	2.118	0.081	0.027*	3.113
MELANOMA	480	0.120	0.145	2.119	0.037	0.021*	3.318
ARGININE AND PROLINE METABOLISM	174	0.045	0.114	2.293	0.008	0.014*	3.504
ENDOMETRIAL CANCER	494	0.152	0.177	1.975	0.006	0.001*	4.269
CHEMOKINE SIGNALING PATHWAY	1127	0.092	0.050	2.712	0.009	0.001*	4.433
LYSINE DEGRADATION	194	0.593	0.720	0.625	0.983	0.984	-0.027
PANTOTHENATE AND COA BIOSYNTHESIS	120	0.403	0.575	0.946	0.974	0.984	0.038
PATHOGENIC ESCHERICHIA COLI INFECTION	240	0.303	0.431	1.253	0.983	0.984	0.031

		Target			Validation		
KEGG Set	Num SNP	Empirical P Enrichment	P_{FDR}	Zscore	Empirical P Enrichment	P_{FDR}	Zscore
BETA ALANINE METABOLISM	136	0.896	0.916	0.157	0.961	0.967	0.067
PROTEASOME	145	0.521	0.692	0.733	0.949	0.966	-0.075
HEDGEHOG SIGNALING PATHWAY	316	0.376	0.464	1.144	0.963	0.966	0.076
NUCLEOTIDE EXCISION REPAIR	180	0.802	0.849	0.278	0.932	0.956	-0.105
ASTHMA	62	0.595	0.742	0.562	0.931	0.956	0.100
ONE CARBON POOL BY FOLATE	96	0.288	0.459	-1.167	0.914	0.956	0.103
FATTY ACID METABOLISM	156	0.306	0.461	-1.159	0.923	0.939	0.140
CYTOKINE CYTOKINE RECEPTOR INTERACTION	923	0.684	0.675	0.765	0.953	0.935	-0.151
JAK STAT SIGNALING PATHWAY	592	0.973	0.976	0.056	0.916	0.931	-0.171
GLYCINE SERINE AND THREONINE METABOLISM	112	0.829	0.868	0.244	0.887	0.931	-0.170
BASAL TRANSCRIPTION FACTORS	130	0.726	0.795	-0.393	0.865	0.931	0.167
SULFUR METABOLISM	87	0.700	0.783	-0.417	0.898	0.931	-0.162
NITROGEN METABOLISM	98	0.359	0.543	1.008	0.889	0.931	0.180
ANTIGEN PROCESSING AND PRESENTATION	154	0.110	0.216	1.806	0.905	0.931	-0.171
VIRAL MYOCARDITIS	374	0.102	0.145	2.110	0.892	0.931	0.193
PYRIMIDINE METABOLISM	414	0.063	0.093	2.422	0.884	0.931	0.188
BASE EXCISION REPAIR	130	0.737	0.801	-0.376	0.824	0.928	0.217
GLYCOSAMINOGLYCAN BIOSYNTHESIS CHONDROITIN SULFATE	218	0.294	0.431	-1.243	0.877	0.927	0.225
NOTCH SIGNALING PATHWAY	306	0.491	0.621	-0.868	0.900	0.922	0.238
DRUG METABOLISM OTHER ENZYMES	225	0.360	0.502	1.089	0.857	0.922	0.236
PROXIMAL TUBULE BICARBONATE RECLAMATION	103	0.853	0.886	0.210	0.790	0.887	-0.289
ARACHIDONIC ACID METABOLISM	243	0.300	0.431	1.251	0.795	0.878	0.307
PROTEIN EXPORT	111	0.038	0.114	2.299	0.781	0.878	0.305
MATURITY ONSET DIABETES OF THE YOUNG	107	0.680	0.767	0.456	0.787	0.874	0.322
FOLATE BIOSYNTHESIS	38	0.372	0.577	-0.929	0.766	0.874	0.323
AMINOACYL TRNA BIOSYNTHESIS	205	0.066	0.139	2.155	0.773	0.855	-0.356
AMINO SUGAR AND NUCLEOTIDE SUGAR METABOLISM	248	0.551	0.692	-0.729	0.748	0.842	0.385
N GLYCAN BIOSYNTHESIS	239	0.258	0.387	1.383	0.736	0.842	-0.383
PPAR SIGNALING PATHWAY	304	0.966	0.976	-0.049	0.765	0.841	0.396
TASTE TRANSDUCTION	203	0.840	0.872	0.233	0.781	0.835	0.409
STEROID BIOSYNTHESIS	69	0.544	0.711	0.655	0.723	0.828	0.430
HYPERTROPHIC CARDIOMYOPATHY HCM	812	0.078	0.065	2.611	0.921	0.828	0.423
BASAL CELL CARCINOMA	277	0.406	0.534	1.030	0.827	0.802	-0.464
CELL ADHESION MOLECULES CAMS	1043	0.301	0.206	1.863	0.759	0.801	-0.471
BIOSYNTHESIS OF UNSATURATED FATTY ACIDS	119	0.440	0.628	-0.848	0.703	0.774	0.510
PURINE METABOLISM	1110	0.308	0.206	1.848	0.862	0.774	0.508
PYRUVATE METABOLISM	173	0.779	0.817	0.323	0.691	0.770	0.525

		Target			Validation		
KEGG Set	Num SNP	Empirical P Enrichment	P_{FDR}	Zscore	Empirical P Enrichment	P_{FDR}	Zscore
GLYCEROPHOSPHOLIPID METABOLISM	413	0.724	0.767	-0.496	0.688	0.770	0.530
THYROID CANCER	186	0.179	0.307	1.562	0.691	0.770	0.524
DNA REPLICATION	142	0.990	0.987	-0.016	0.628	0.765	-0.577
STEROID HORMONE BIOSYNTHESIS	180	0.972	0.976	0.038	0.709	0.765	-0.548
GLYCOSYLPHOSPHATIDYLINOSITOL GPI ANCHOR BIOSYNTHESIS	91	0.748	0.812	0.346	0.625	0.765	-0.550
GLUTATHIONE METABOLISM	167	0.573	0.712	-0.646	0.689	0.765	0.564
PENTOSE PHOSPHATE PATHWAY	86	0.533	0.709	0.677	0.662	0.765	0.554
GRAFT VERSUS HOST DISEASE	95	0.429	0.626	0.857	0.654	0.765	0.571
CELL CYCLE	524	0.333	0.383	1.398	0.850	0.765	-0.548
PRIMARY BILE ACID BIOSYNTHESIS	86	0.861	0.897	-0.190	0.641	0.759	0.596
OTHER GLYCAN DEGRADATION	50	0.348	0.555	-0.981	0.601	0.736	0.627
ASCORBATE AND ALDARATE METABOLISM	54	0.588	0.742	-0.560	0.569	0.699	-0.681
TYROSINE METABOLISM	143	0.168	0.308	1.547	0.579	0.687	0.700
GLYCOSPHINGOLIPID BIOSYNTHESIS GLOBO SERIES	108	0.942	0.963	0.085	0.513	0.670	-0.726
GLYCEROLIPID METABOLISM	295	0.742	0.783	-0.414	0.614	0.669	0.732
DILATED CARDIOMYOPATHY	887	0.073	0.050	2.713	0.866	0.664	0.744
PRION DISEASES	177	0.647	0.757	0.532	0.502	0.662	0.756
TYPE I DIABETES MELLITUS	201	0.954	0.974	0.065	0.490	0.661	-0.776
OLFACTORY TRANSDUCTION	491	0.943	0.956	-0.100	0.529	0.661	0.797
ALPHA LINOLENIC ACID METABOLISM	82	0.689	0.780	-0.430	0.477	0.661	0.800
RENIN ANGIOTENSIN SYSTEM	85	0.631	0.757	-0.525	0.527	0.661	-0.801
ADIPOCYTOKINE SIGNALING PATHWAY	370	0.637	0.720	0.631	0.554	0.661	-0.796
ALLOGRAFT REJECTION	99	0.230	0.405	1.318	0.490	0.661	-0.776
AUTOIMMUNE THYROID DISEASE	143	0.236	0.401	1.334	0.490	0.661	-0.776
INTESTINAL IMMUNE NETWORK FOR IGA PRODUCTION	157	0.242	0.401	1.337	0.572	0.661	0.782
ARRHYTHMOGENIC RIGHT VENTRICULAR CARDIOMYOPATHY ARVC	995	0.169	0.116	2.276	0.850	0.661	0.771
ADHERENS JUNCTION	769	0.105	0.093	2.428	0.834	0.661	0.765
ALANINE ASPARTATE AND GLUTAMATE METABOLISM	156	0.772	0.814	-0.332	0.545	0.648	-0.841
GLYCOSAMINOGLYCAN BIOSYNTHESIS HEPARAN SULFATE	223	0.386	0.536	1.022	0.479	0.648	-0.851
CYSTEINE AND METHIONINE METABOLISM	148	0.161	0.306	1.572	0.506	0.648	0.841
PROPANOATE METABOLISM	190	0.693	0.767	-0.458	0.450	0.645	0.862
VALINE LEUCINE AND ISOLEUCINE DEGRADATION	217	0.425	0.575	0.948	0.450	0.645	0.862
STARCH AND SUCROSE METABOLISM	206	0.685	0.767	-0.478	0.458	0.630	0.891
TAURINE AND HYPOTAURINE METABOLISM	26	0.640	0.767	0.487	0.437	0.629	-0.902
TYPE II DIABETES MELLITUS	419	0.407	0.464	1.147	0.723	0.627	0.914
GLYOXYLATE AND DICARBOXYLATE METABOLISM	83	0.531	0.709	0.675	0.460	0.608	0.942

		Target			Validation		
KEGG Set	Num SNP	Empirical P Enrichment	P_{FDR}	Zscore	Empirical P Enrichment	P_{FDR}	Zscore
REGULATION OF AUTOPHAGY	69	0.603	0.742	0.564	0.423	0.573	0.987
NOD LIKE RECEPTOR SIGNALING PATHWAY	260	0.557	0.693	0.722	0.381	0.573	-1.000
PROGESTERONE MEDIATED OOCYTE MATURATION	539	0.159	0.179	1.961	0.609	0.573	0.990
GLYCOSPHINGOLIPID BIOSYNTHESIS LACTO AND NEOLACTO SERIES	137	0.067	0.151	2.077	0.468	0.573	0.990
NON HOMOLOGOUS END JOINING	69	0.331	0.528	1.044	0.391	0.539	1.057
CITRATE CYCLE TCA CYCLE	133	0.270	0.431	1.243	0.438	0.539	1.052
BLADDER CANCER	218	0.429	0.575	0.939	0.367	0.523	1.084
ENDOCYTOSIS	1140	0.569	0.448	1.200	0.626	0.511	1.112
HISTIDINE METABOLISM	106	0.555	0.711	-0.654	0.353	0.508	-1.122
NATURAL KILLER CELL MEDIATED CYTOTOXICITY	625	0.226	0.216	1.802	0.570	0.497	1.141
DORSO VENTRAL AXIS FORMATION	208	0.892	0.916	0.155	0.307	0.495	1.150
WNT SIGNALING PATHWAY	948	0.167	0.123	2.231	0.825	0.495	1.157
HEMATOPOIETIC CELL LINEAGE	364	0.800	0.812	-0.340	0.331	0.488	-1.178
GLYCOSAMINOGLYCAN DEGRADATION	118	0.683	0.767	0.457	0.280	0.474	-1.202
VIBRIO CHOLERAE INFECTION	366	0.185	0.236	1.718	0.445	0.465	1.220
MELANOGENESIS	661	0.201	0.193	1.921	0.716	0.454	1.241
PRIMARY IMMUNODEFICIENCY	113	0.516	0.695	0.713	0.302	0.451	1.251
B CELL RECEPTOR SIGNALING PATHWAY	504	0.417	0.455	1.179	0.373	0.438	1.281
RETINOL METABOLISM	172	0.190	0.318	1.523	0.288	0.438	-1.288
CARDIAC MUSCLE CONTRACTION	555	0.042	0.050	2.716	0.653	0.438	1.300
SNARE INTERACTIONS IN VESICULAR TRANSPORT	172	0.824	0.868	0.246	0.236	0.427	1.326
MISMATCH REPAIR	118	0.728	0.795	0.389	0.315	0.427	1.343
FC EPSILON RI SIGNALING PATHWAY	563	0.187	0.201	1.895	0.457	0.427	1.323
ACUTE MYELOID LEUKEMIA	381	0.066	0.100	2.372	0.452	0.427	1.335
P53 SIGNALING PATHWAY	319	0.731	0.777	-0.438	0.306	0.397	1.397
ALDOSTERONE REGULATED SODIUM REABSORPTION	324	0.156	0.216	1.792	0.400	0.393	1.410
RIBOFLAVIN METABOLISM	61	0.587	0.742	0.576	0.240	0.392	1.429
EPITHELIAL CELL SIGNALING IN HELICOBACTER PYLORI INFECTION	377	0.119	0.163	2.017	0.349	0.392	1.422
PORPHYRIN AND CHLOROPHYLL METABOLISM	125	0.654	0.767	-0.503	0.181	0.346	-1.506
PENTOSE AND GLUCURONATE INTERCONVERSIONS	75	0.627	0.757	0.531	0.181	0.346	-1.506
PHENYLALANINE METABOLISM	56	0.572	0.739	0.587	0.216	0.346	-1.509
NICOTINATE AND NICOTINAMIDE METABOLISM	118	0.063	0.149	2.090	0.257	0.346	-1.512
O GLYCAN BIOSYNTHESIS	266	0.332	0.446	1.209	0.257	0.340	-1.544
BUTANOATE METABOLISM	133	0.692	0.767	0.462	0.191	0.317	1.588
TIGHT JUNCTION	1148	0.557	0.431	1.248	0.520	0.302	1.626
GNRH SIGNALING PATHWAY	812	0.100	0.091	2.467	0.522	0.293	1.664

		Target			Validation		
KEGG Set	Num SNP	Empirical P Enrichment	P_{FDR}	Zscore	Empirical P Enrichment	P_{FDR}	Zscore
CYTOSOLIC DNA SENSING PATHWAY	145	0.509	0.685	0.748	0.210	0.284	1.699
RNA POLYMERASE	97	0.439	0.630	0.839	0.192	0.284	1.686
VASOPRESSIN REGULATED WATER REABSORPTION	243	0.254	0.387	1.373	0.240	0.284	1.688
T CELL RECEPTOR SIGNALING PATHWAY	622	0.229	0.216	1.787	0.326	0.284	1.694
APOPTOSIS	415	0.385	0.451	1.190	0.198	0.269	1.748
LYSOSOME	557	0.408	0.434	1.232	0.200	0.269	1.744
SPHINGOLIPID METABOLISM	208	0.762	0.810	-0.354	0.140	0.260	1.803
SELENOAMINO ACID METABOLISM	98	0.740	0.806	0.365	0.106	0.260	-1.786
LIMONENE AND PINENE DEGRADATION	31	0.444	0.648	-0.802	0.129	0.260	1.795
PEROXISOME	347	0.534	0.641	-0.816	0.163	0.260	1.811
ABC TRANSPORTERS	360	0.087	0.134	2.179	0.109	0.260	-1.777
PROSTATE CANCER	574	0.113	0.123	2.221	0.328	0.255	1.831
FC GAMMA R MEDIATED PHAGOCYTOSIS	761	0.141	0.123	2.225	0.341	0.254	1.842
ECM RECEPTOR INTERACTION	703	0.412	0.405	1.315	0.278	0.249	1.866
GLIOMA	486	0.206	0.229	1.739	0.307	0.249	1.859
ETHER LIPID METABOLISM	145	0.695	0.767	-0.460	0.093	0.242	1.892
UBIQUITIN MEDIATED PROTEOLYSIS	701	0.548	0.551	0.992	0.311	0.225	1.933
SPLICEOSOME	409	0.430	0.509	1.073	0.132	0.211	1.970
NON SMALL CELL LUNG CANCER	536	0.245	0.263	1.659	0.263	0.211	1.973
RIG I LIKE RECEPTOR SIGNALING PATHWAY	229	0.142	0.222	1.761	0.172	0.211	1.987
LEISHMANIA INFECTION	279	0.250	0.365	1.433	0.101	0.202	-2.019
HOMOLOGOUS RECOMBINATION	181	0.031	0.091	2.480	0.155	0.202	2.028
FRUCTOSE AND MANNOSE METABOLISM	196	0.242	0.387	-1.374	0.128	0.185	2.076
TGF BETA SIGNALING PATHWAY	421	0.064	0.093	2.428	0.212	0.179	-2.101
GLYCOSAMINOGLYCAN BIOSYNTHESIS KERATAN SULFATE	75	0.252	0.431	1.242	0.089	0.174	2.124
OXIDATIVE PHOSPHORYLATION	410	0.160	0.206	1.845	0.091	0.152	2.189
OOCYTE MEIOSIS	661	0.292	0.289	1.606	0.205	0.135	2.247
TERPENOID BACKBONE BIOSYNTHESIS	52	0.140	0.308	1.552	0.059	0.113	2.325
NEUROTROPHIN SIGNALING PATHWAY	762	0.352	0.330	1.499	0.167	0.108	2.354
NEUROACTIVE LIGAND RECEPTOR INTERACTION	1671	0.860	0.720	0.621	0.213	0.100	2.393
LINOLEIC ACID METABOLISM	101	0.102	0.216	1.814	0.058	0.086	2.463
SMALL CELL LUNG CANCER	704	0.165	0.151	2.057	0.191	0.086	2.486
ALZHEIMERS DISEASE	901	0.134	0.104	2.348	0.251	0.086	2.461
PARKINSONS DISEASE	432	0.168	0.206	1.846	0.040	0.073	2.570
METABOLISM OF XENOBIOTICS BY CYTOCHROME P450	196	0.572	0.711	0.659	0.061	0.071	2.603
GALACTOSE METABOLISM	147	0.556	0.709	0.673	0.029	0.071	2.605
GLYCOLYSIS GLUCONEOGENESIS	208	0.552	0.706	0.695	0.036	0.071	2.623

		Target			Validation		
**KEGG Set**	**Num SNP**	**Empirical P Enrichment**	**P_{FDR}**	**Zscore**	**Empirical P Enrichment**	**P_{FDR}**	**Zscore**
AXON GUIDANCE	1374	0.754	0.630	0.834	0.238	0.071	2.642
DRUG METABOLISM CYTOCHROME P450	184	0.426	0.577	0.925	0.060	0.071	2.590
REGULATION OF ACTIN CYTOSKELETON	1404	0.196	0.100	2.372	0.137	0.066	2.692
GLYCOSPHINGOLIPID BIOSYNTHESIS GANGLIO SERIES	142	0.605	0.738	-0.594	0.028	0.057	2.755

Table S2. Results of Pathway-specific PRS analysis for the 186 KEGG (Kyoto Encyclopaedia of Genes and Genomes) pathways considered in the analysis both for target and validation cohort. Set, Name of KEGG Set; Empirical-P, empirical-competitive p-value derived by applying 10k permutation; PFDR, False Discovery Rate p-value; Z score, the ratio between the PRS effect size and the standard error of the corresponding logistic regression, * FDR<0.05

Genes	CALCIUM SIGNALING	FOCAL ADHESION	HUNTINGTONS DISEASE	INOSITOL PHOSPHATE METABOLISM	MTOR SIGNALING PATHWAY	PHOSPHATIDYLINOSITOL	VEGF SIGNALING	Total Pathways Shared
Total genes by pathway	185	218	184	57	50	84	80	
PIK3CA		X		X	X	X	X	5
PIK3CB		X		X	X	X	X	5
PIK3CD		X		X	X	X	X	5
PIK3CG		X		X	X	X	X	5
PRKCA	X	X				X	X	4
PRKCB	X	X				X	X	4
PRKCG	X	X				X	X	4
PLCB1	X		X	X		X		4
PLCB2	X		X	X		X		4
PLCB3	X		X	X		X		4
PLCB4	X		X	X		X		4
PLCG1	X			X		X	X	4
PLCG2	X			X		X	X	4
PIK3R1		X			X	X	X	4
PIK3R2		X			X	X	X	4
PIK3R3		X			X	X	X	4

Genes	CALCIUM SIGNALING	FOCAL ADHESION	HUNTINGTONS DISEASE	INOSITOL PHOSPHATE METABOLISM	MTOR SIGNALING PATHWAY	PHOSPHATIDYLINOSITOL	VEGF SIGNALING	Total Pathways Shared
PIK3R5		X			X	X	X	4
TSPAN1		X			X	X	X	4
ITPR1	X		X			X		3
ITPKA	X			X		X		3
ITPKB	X			X		X		3
PLCD1	X			X		X		3
PLCD3	X			X		X		3
PLCD4	X			X		X		3
PLCE1	X			X		X		3
PLCZ1	X			X		X		3
PSMC1P9	X			X		X		3
AKT1		X			X		X	3
AKT2		X			X		X	3
AKT3		X			X		X	3
MAPK1		X			X		X	3
MAPK3		X			X		X	3
SDCCAG8		X			X		X	3

Genes	CALCIUM SIGNALING	FOCAL ADHESION	HUNTINGTONS DISEASE	INOSITOL PHOSPHATE METABOLISM	MTOR SIGNALING PATHWAY	PHOSPHATIDYLINOSITOL	VEGF SIGNALING	Total Pathways Shared
VEGFA		X			X		X	3
PIP5K1C		X		X		X		3
PTEN		X		X		X		3
CAV3	X	X						2
EGFR	X	X						2
ERBB2	X	X						2
FIP1L1	X	X						2
MIEN1	X	X						2
MYLK	X	X						2
MYLK2	X	X						2
MYLK3	X	X						2
OXTR	X	X						2
PDGFRA	X	X						2
PDGFRB	X	X						2
PGAP3	X	X						2
GNAQ	X		X					2
GRM5	X		X					2

Genes	CALCIUM SIGNALING	FOCAL ADHESION	HUNTINGTONS DISEASE	INOSITOL PHOSPHATE METABOLISM	MTOR SIGNALING PATHWAY	PHOSPHATIDYLINOSITOL	VEGF SIGNALING	Total Pathways Shared
PPID	X		X					2
SLC25A31	X		X					2
SLC25A4	X		X					2
VDAC1	X		X					2
VDAC2	X		X					2
VDAC3	X		X					2
CALM1	X					X		2
CALM2	X					X		2
CALM3	X					X		2
CALML3	X					X		2
CALML5	X					X		2
CALML6	X					X		2
CHP2	X						X	2
GRIN3A	X						X	2
ITPR2	X					X		2
ITPR3	X					X		2
NOS3	X						X	2

Genes	CALCIUM SIGNALING	FOCAL ADHESION	HUNTINGTONS DISEASE	INOSITOL PHOSPHATE METABOLISM	MTOR SIGNALING PATHWAY	PHOSPHATIDYLINOSITOL	VEGF SIGNALING	Total Pathways Shared
PPP3CA	X						X	2
PPP3CB	X						X	2
PPP3CC	X						X	2
PPP3R1	X						X	2
PPP3R2	X						X	2
PRPSAP1	X						X	2
SPHK1	X						X	2
SPHK2	X						X	2
BRAF		X			X			2
IGF1		X			X			2
PDPK1		X			X			2
PGF		X			X			2
VEGFC		X			X			2
BAD		X					X	2
CDC42		X					X	2
GPR137		X					X	2
KDR		X					X	2

Genes	CALCIUM SIGNALING	FOCAL ADHESION	HUNTINGTONS DISEASE	INOSITOL PHOSPHATE METABOLISM	MTOR SIGNALING PATHWAY	PHOSPHATIDYLINOSITOL	VEGF SIGNALING	Total Pathways Shared
MAP2K1		X					X	2
PTK2		X					X	2
PXN		X					X	2
RAC1		X					X	2
RAC2		X					X	2
RAC3		X					X	2
RAF1		X					X	2
SHC2		X					X	2
SRC		X					X	2
CASP9			X				X	2
CDIPT				X		X		2
CENPP				X		X		2
IMPA1				X		X		2
IMPA2				X		X		2
INPP1				X		X		2
INPP4A				X		X		2
INPP4B				X		X		2

Genes	CALCIUM SIGNALING	FOCAL ADHESION	HUNTINGTONS DISEASE	INOSITOL PHOSPHATE METABOLISM	MTOR SIGNALING PATHWAY	PHOSPHATIDYLINOSITOL	VEGF SIGNALING	Total Pathways Shared
INPP5A				X		X		2
INPP5B				X		X		2
INPP5E				X		X		2
INPP5J				X		X		2
INPP5K				X		X		2
INPPL1				X		X		2
IPPK				X		X		2
ITPK1				X		X		2
PI4KA				X		X		2
PI4KB				X		X		2
PIK3C2A				X		X		2
PIK3C2B				X		X		2
PIK3C2G				X		X		2
PIK3C3				X		X		2
PIKFYVE				X		X		2
PIP4K2A				X		X		2
PIP4K2B				X		X		2

Genes	CALCIUM SIGNALING	FOCAL ADHESION	HUNTINGTONS DISEASE	INOSITOL PHOSPHATE METABOLISM	MTOR SIGNALING PATHWAY	PHOSPHATIDYLINOSITOL	VEGF SIGNALING	Total Pathways Shared
PIP4K2C				X		X		2
PIP5K1A				X		X		2
PIP5K1B				X		X		2
RERGL				X		X		2
SERPIND1				X		X		2
SYNJ1				X		X		2
SYNJ2				X		X		2
ADCY1	X							1
ADCY2	X							1
ADCY3	X							1
ADCY4	X							1
ADCY7	X							1
ADCY8	X							1
ADCY9	X							1
ADORA2A	X							1
ADORA2B	X							1
ADRA1A	X							1

Genes	CALCIUM SIGNALING	FOCAL ADHESION	HUNTINGTONS DISEASE	INOSITOL PHOSPHATE METABOLISM	MTOR SIGNALING PATHWAY	PHOSPHATIDYLINOSITOL	VEGF SIGNALING	Total Pathways Shared
ADRA1B	X							1
ADRA1D	X							1
ADRB1	X							1
ADRB2	X							1
ADRB3	X							1
AGTR1	X							1
ATP2A1	X							1
ATP2A2	X							1
ATP2A3	X							1
ATP2B1	X							1
ATP2B2	X							1
ATP2B4	X							1
AVEN	X							1
AVPR1A	X							1
AVPR1B	X							1
BDKRB1	X							1
BDKRB2	X							1

Genes	CALCIUM SIGNALING	FOCAL ADHESION	HUNTINGTONS DISEASE	INOSITOL PHOSPHATE METABOLISM	MTOR SIGNALING PATHWAY	PHOSPHATIDYLINOSITOL	VEGF SIGNALING	Total Pathways Shared
BRD7	X							1
BST1	X							1
C16orf93	X							1
CACNA1A	X							1
CACNA1C	X							1
CACNA1D	X							1
CACNA1E	X							1
CACNA1G	X							1
CACNA1H	X							1
CACNA1I	X							1
CACNA1S	X							1
CAMK2A	X							1
CAMK2B	X							1
CAMK2D	X							1
CAMK2G	X							1
CAMK4	X							1
CCKAR	X							1

Genes	CALCIUM SIGNALING	FOCAL ADHESION	HUNTINGTONS DISEASE	INOSITOL PHOSPHATE METABOLISM	MTOR SIGNALING PATHWAY	PHOSPHATIDYLINOSITOL	VEGF SIGNALING	Total Pathways Shared
CCKBR	X							1
CD38	X							1
CENPO	X							1
CEP85L	X							1
CHRM1	X							1
CHRM2	X							1
CHRM3	X							1
CHRM5	X							1
CHRNA7	X							1
CIDEB	X							1
CYSLTR2	X							1
DCP1B	X							1
DRD1	X							1
EDNRA	X							1
EDNRB	X							1
ERBB3	X							1
ERBB4	X							1

Genes	CALCIUM SIGNALING	FOCAL ADHESION	HUNTINGTONS DISEASE	INOSITOL PHOSPHATE METABOLISM	MTOR SIGNALING PATHWAY	PHOSPHATIDYLINOSITOL	VEGF SIGNALING	Total Pathways Shared
F2R	X							1
FAM200B	X							1
GNA11	X							1
GNA14	X							1
GNA15	X							1
GNAL	X							1
GNAS	X							1
GRIN2A	X							1
GRIN2C	X							1
GRIN2D	X							1
GRM1	X							1
GTF2A1L	X							1
HRH1	X							1
HRH2	X							1
HTR2A	X							1
HTR2B	X							1
HTR4	X							1

Genes	CALCIUM SIGNALING	FOCAL ADHESION	HUNTINGTONS DISEASE	INOSITOL PHOSPHATE METABOLISM	MTOR SIGNALING PATHWAY	PHOSPHATIDYLINOSITOL	VEGF SIGNALING	Total Pathways Shared
HTR5A	X							1
HTR6	X							1
HTR7	X							1
ITFG1	X							1
LHCGR	X							1
LTB4R2	X							1
NOP9	X							1
NOS1	X							1
NOS2	X							1
NTSR1	X							1
P2RX1	X							1
P2RX2	X							1
P2RX3	X							1
P2RX4	X							1
P2RX5	X							1
P2RX6	X							1
P2RX7	X							1

Genes	CALCIUM SIGNALING	FOCAL ADHESION	HUNTINGTONS DISEASE	INOSITOL PHOSPHATE METABOLISM	MTOR SIGNALING PATHWAY	PHOSPHATIDYLINOSITOL	VEGF SIGNALING	Total Pathways Shared
PDE1A	X							1
PDE1B	X							1
PDE1C	X							1
PHKB	X							1
PHKG1	X							1
PHKG2	X							1
PLN	X							1
PPP1R1A	X							1
PRKACA	X							1
PRKACB	X							1
PRKACG	X							1
PSMD1	X							1
PTAFR	X							1
PTGER1	X							1
PTGER3	X							1
PTGFR	X							1
PTK2B	X							1

Genes	CALCIUM SIGNALING	FOCAL ADHESION	HUNTINGTONS DISEASE	INOSITOL PHOSPHATE METABOLISM	MTOR SIGNALING PATHWAY	PHOSPHATIDYLINOSITOL	VEGF SIGNALING	Total Pathways Shared
RYR1	X							1
RYR2	X							1
RYR3	X							1
SLC6A7	X							1
SLC8A1	X							1
SLC8A2	X							1
SLC8A3	X							1
TACR1	X							1
TACR2	X							1
TACR3	X							1
TBXA2R	X							1
TNNC1	X							1
TNNC2	X							1
TRHR	X							1
TRPC1	X							1
TUBA3FP	X							1
ACSF2		X						1

Genes	CALCIUM SIGNALING	FOCAL ADHESION	HUNTINGTONS DISEASE	INOSITOL PHOSPHATE METABOLISM	MTOR SIGNALING PATHWAY	PHOSPHATIDYLINOSITOL	VEGF SIGNALING	Total Pathways Shared
ACTB		X						1
ACTG1		X						1
ACTN1		X						1
ACTN2		X						1
ACTN3		X						1
ACTN4		X						1
ADORA3		X						1
ARHGAP35		X						1
ARHGAP5		X						1
ATF6B		X						1
BCAR1		X						1
BCL2		X						1
BIRC2		X						1
BIRC3		X						1
BUB1B		X						1
CAPN2		X						1
CAV1		X						1

Genes	CALCIUM SIGNALING	FOCAL ADHESION	HUNTINGTONS DISEASE	INOSITOL PHOSPHATE METABOLISM	MTOR SIGNALING PATHWAY	PHOSPHATIDYLINOSITOL	VEGF SIGNALING	Total Pathways Shared
CAV2		X						1
CCND1		X						1
CCND2		X						1
CCND3		X						1
CHAD		X						1
COL11A1		X						1
COL11A2		X						1
COL1A1		X						1
COL1A2		X						1
COL2A1		X						1
COL3A1		X						1
COL4A1		X						1
COL4A2		X						1
COL4A4		X						1
COL5A1		X						1
COL5A2		X						1
COL5A3		X						1

Genes	CALCIUM SIGNALING	FOCAL ADHESION	HUNTINGTONS DISEASE	INOSITOL PHOSPHATE METABOLISM	MTOR SIGNALING PATHWAY	PHOSPHATIDYLINOSITOL	VEGF SIGNALING	Total Pathways Shared
COL6A1		X						1
COL6A2		X						1
COL6A3		X						1
COL6A6		X						1
COMP		X						1
CRK		X						1
CRKL		X						1
CTNNB1		X						1
DIAPH1		X						1
DOCK1		X						1
EFCAB13		X						1
EGF		X						1
EPHA1		X						1
FAM196A		X						1
FLNB		X						1
FLNC		X						1
FLT1		X						1

Genes	CALCIUM SIGNALING	FOCAL ADHESION	HUNTINGTONS DISEASE	INOSITOL PHOSPHATE METABOLISM	MTOR SIGNALING PATHWAY	PHOSPHATIDYLINOSITOL	VEGF SIGNALING	Total Pathways Shared
FLT4		X						1
FN1		X						1
FYN		X						1
GALK1		X						1
GRB2		X						1
GSK3B		X						1
HGF		X						1
IBSP		X						1
IGF1R		X						1
ILK		X						1
ITGA1		X						1
ITGA10		X						1
ITGA11		X						1
ITGA2		X						1
ITGA2B		X						1
ITGA3		X						1
ITGA4		X						1

Genes	CALCIUM SIGNALING	FOCAL ADHESION	HUNTINGTONS DISEASE	INOSITOL PHOSPHATE METABOLISM	MTOR SIGNALING PATHWAY	PHOSPHATIDYLINOSITOL	VEGF SIGNALING	Total Pathways Shared
ITGA5		X						1
ITGA6		X						1
ITGA7		X						1
ITGA8		X						1
ITGA9		X						1
ITGAV		X						1
ITGB1		X						1
ITGB3		X						1
ITGB4		X						1
ITGB5		X						1
ITGB6		X						1
ITGB7		X						1
ITGB8		X						1
JUN		X						1
LAMA1		X						1
LAMA2		X						1
LAMA3		X						1

Genes	CALCIUM SIGNALING	FOCAL ADHESION	HUNTINGTONS DISEASE	INOSITOL PHOSPHATE METABOLISM	MTOR SIGNALING PATHWAY	PHOSPHATIDYLINOSITOL	VEGF SIGNALING	Total Pathways Shared
LAMA4		X						1
LAMA5		X						1
LAMB1		X						1
LAMB2		X						1
LAMB3		X						1
LAMB4		X						1
LAMC1		X						1
LAMC2		X						1
LAMC3		X						1
LINC01160		X						1
MAPK10		X						1
MAPK8		X						1
MAPK9		X						1
MET		X						1
MYL10		X						1
MYL12B		X						1
MYL2		X						1

Genes	CALCIUM SIGNALING	FOCAL ADHESION	HUNTINGTONS DISEASE	INOSITOL PHOSPHATE METABOLISM	MTOR SIGNALING PATHWAY	PHOSPHATIDYLINOSITOL	VEGF SIGNALING	Total Pathways Shared
MYL5		X						1
MYL7		X						1
MYL9		X						1
MYLPF		X						1
ORAOV1		X						1
PAK1		X						1
PAK2		X						1
PAK4		X						1
PAK6		X						1
PAK7		X						1
PARVA		X						1
PARVB		X						1
PARVG		X						1
PDGFB		X						1
PDGFC		X						1
PDGFD		X						1
PELO		X						1

Genes	CALCIUM SIGNALING	FOCAL ADHESION	HUNTINGTONS DISEASE	INOSITOL PHOSPHATE METABOLISM	MTOR SIGNALING PATHWAY	PHOSPHATIDYLINOSITOL	VEGF SIGNALING	Total Pathways Shared
PPP1CA		X						1
PPP1CB		X						1
PPP1CC		X						1
PPP1R12A		X						1
RAD18		X						1
RAD9A		X						1
RAP1A		X						1
RAP1B		X						1
RAPGEF1		X						1
RASGRF1		X						1
RELN		X						1
RHOA		X						1
ROCK1		X						1
ROCK2		X						1
SAMM50		X						1
SARM1		X						1
SHC1		X						1

Genes	CALCIUM SIGNALING	FOCAL ADHESION	HUNTINGTONS DISEASE	INOSITOL PHOSPHATE METABOLISM	MTOR SIGNALING PATHWAY	PHOSPHATIDYLINOSITOL	VEGF SIGNALING	Total Pathways Shared
SHC3		X						1
SHC4		X						1
SOS1		X						1
SOS2		X						1
SPDYA		X						1
SPP1		X						1
SSUH2		X						1
TAF10		X						1
TBC1D10C		X						1
THBS1		X						1
THBS2		X						1
THBS3		X						1
THBS4		X						1
TLN1		X						1
TLN2		X						1
TMEM199		X						1
TNC		X						1

Genes	CALCIUM SIGNALING	FOCAL ADHESION	HUNTINGTONS DISEASE	INOSITOL PHOSPHATE METABOLISM	MTOR SIGNALING PATHWAY	PHOSPHATIDYLINOSITOL	VEGF SIGNALING	Total Pathways Shared
TNN		X						1
TNR		X						1
TNXB		X						1
U4		X						1
ULK4		X						1
VASP		X						1
VAV1		X						1
VAV2		X						1
VAV3		X						1
VCL		X						1
VTN		X						1
VWF		X						1
ZYX		X						1
ADAMTS4			X					1
ADCK2			X					1
ANKS1B			X					1
AP2A1			X					1

Genes	CALCIUM SIGNALING	FOCAL ADHESION	HUNTINGTONS DISEASE	INOSITOL PHOSPHATE METABOLISM	MTOR SIGNALING PATHWAY	PHOSPHATIDYLINOSITOL	VEGF SIGNALING	Total Pathways Shared
AP2A2			X					1
AP2B1			X					1
AP2M1			X					1
APAF1			X					1
ARL10			X					1
ATP5A1			X					1
ATP5B			X					1
ATP5C1			X					1
ATP5E			X					1
ATP5F1			X					1
ATP5G1			X					1
ATP5G2			X					1
ATP5G3			X					1
ATP5H			X					1
ATP5J			X					1
ATP5O			X					1
BAX			X					1

Genes	CALCIUM SIGNALING	FOCAL ADHESION	HUNTINGTONS DISEASE	INOSITOL PHOSPHATE METABOLISM	MTOR SIGNALING PATHWAY	PHOSPHATIDYLINOSITOL	VEGF SIGNALING	Total Pathways Shared
BCO2			X					1
BDNF			X					1
CASP3			X					1
CASP8			X					1
CLTA			X					1
CLTB			X					1
CLTC			X					1
CLTCL1			X					1
COX4I2			X					1
COX5A			X					1
COX5B			X					1
COX6A1			X					1
COX6A2			X					1
COX6B1			X					1
COX6C			X					1
COX7A1			X					1
COX7A2			X					1

Genes	CALCIUM SIGNALING	FOCAL ADHESION	HUNTINGTONS DISEASE	INOSITOL PHOSPHATE METABOLISM	MTOR SIGNALING PATHWAY	PHOSPHATIDYLINOSITOL	VEGF SIGNALING	Total Pathways Shared
COX7A2L			X					1
COX7B2			X					1
COX7C			X					1
COX8C			X					1
CREB1			X					1
CREB3			X					1
CREB3L1			X					1
CREB3L2			X					1
CREB3L3			X					1
CREB3L4			X					1
CREB5			X					1
CREBBP			X					1
CYCS			X					1
DCTN1			X					1
DCTN2			X					1
DCTN4			X					1
DLG4			X					1

Genes	CALCIUM SIGNALING	FOCAL ADHESION	HUNTINGTONS DISEASE	INOSITOL PHOSPHATE METABOLISM	MTOR SIGNALING PATHWAY	PHOSPHATIDYLINOSITOL	VEGF SIGNALING	Total Pathways Shared
DNAH1			X					1
DNAH2			X					1
DNAH3			X					1
DNAI1			X					1
DNAI2			X					1
DNAL1			X					1
DNAL4			X					1
DNALI1			X					1
EIF2B5			X					1
EP300			X					1
FAM219A			X					1
GDF9			X					1
GNE			X					1
GPX1			X					1
GRIN2B			X					1
HAP1			X					1
HDAC1			X					1

Genes	CALCIUM SIGNALING	FOCAL ADHESION	HUNTINGTONS DISEASE	INOSITOL PHOSPHATE METABOLISM	MTOR SIGNALING PATHWAY	PHOSPHATIDYLINOSITOL	VEGF SIGNALING	Total Pathways Shared
HDAC2			X					1
HIP1			X					1
HTT			X					1
IFT57			X					1
IK			X					1
IL18			X					1
JUP			X					1
KCTD2			X					1
MBD3			X					1
MGARP			X					1
MTSS1			X					1
NDUFA10			X					1
NDUFA2			X					1
NDUFA3			X					1
NDUFA4			X					1
NDUFA5			X					1
NDUFA6			X					1

Genes	CALCIUM SIGNALING	FOCAL ADHESION	HUNTINGTONS DISEASE	INOSITOL PHOSPHATE METABOLISM	MTOR SIGNALING PATHWAY	PHOSPHATIDYLINOSITOL	VEGF SIGNALING	Total Pathways Shared
NDUFA7			X					1
NDUFA8			X					1
NDUFA9			X					1
NDUFAB1			X					1
NDUFB1			X					1
NDUFB10			X					1
NDUFB2			X					1
NDUFB3			X					1
NDUFB5			X					1
NDUFB6			X					1
NDUFB7			X					1
NDUFB8			X					1
NDUFB9			X					1
NDUFC1			X					1
NDUFC2			X					1
NDUFS1			X					1
NDUFS2			X					1

Genes	CALCIUM SIGNALING	FOCAL ADHESION	HUNTINGTONS DISEASE	INOSITOL PHOSPHATE METABOLISM	MTOR SIGNALING PATHWAY	PHOSPHATIDYLINOSITOL	VEGF SIGNALING	Total Pathways Shared
NDUFS3			X					1
NDUFS4			X					1
NDUFS5			X					1
NDUFS6			X					1
NDUFS7			X					1
NDUFS8			X					1
NDUFV1			X					1
NDUFV2			X					1
NDUFV3			X					1
NOA1			X					1
NRF1			X					1
POLR2A			X					1
POLR2B			X					1
POLR2C			X					1
POLR2D			X					1
POLR2E			X					1
POLR2F			X					1

Genes	CALCIUM SIGNALING	FOCAL ADHESION	HUNTINGTONS DISEASE	INOSITOL PHOSPHATE METABOLISM	MTOR SIGNALING PATHWAY	PHOSPHATIDYLINOSITOL	VEGF SIGNALING	Total Pathways Shared
POLR2H			X					1
POLR2I			X					1
POLR2J			X					1
POLR2K			X					1
PPARG			X					1
PPARGC1A			X					1
PTPMT1			X					1
PTRH2			X					1
RCOR1			X					1
REST			X					1
RPL29P2			X					1
SDHA			X					1
SDHB			X					1
SDHC			X					1
SDHD			X					1
SEC31B			X					1
SIN3A			X					1

Genes	CALCIUM SIGNALING	FOCAL ADHESION	HUNTINGTONS DISEASE	INOSITOL PHOSPHATE METABOLISM	MTOR SIGNALING PATHWAY	PHOSPHATIDYLINOSITOL	VEGF SIGNALING	Total Pathways Shared
SOD1			X					1
SOD2			X					1
SOX10			X					1
SP1			X					1
SUMO2P17			X					1
SUN2			X					1
TAF4			X					1
TAF4B			X					1
TBCB			X					1
TBP			X					1
TBPL1			X					1
TBPL2			X					1
TEX12			X					1
TFAM			X					1
TFPT			X					1
TGM2			X					1
TP53			X					1

Genes	CALCIUM SIGNALING	FOCAL ADHESION	HUNTINGTONS DISEASE	INOSITOL PHOSPHATE METABOLISM	MTOR SIGNALING PATHWAY	PHOSPHATIDYLINOSITOL	VEGF SIGNALING	Total Pathways Shared
UCP1			X					1
UNC79			X					1
UQCR10			X					1
UQCR11			X					1
UQCRB			X					1
UQCRC1			X					1
UQCRC2			X					1
UQCRFS1			X					1
UQCRH			X					1
UQCRQ			X					1
VPS13B			X					1
WDR77			X					1
WTAP			X					1
ANGPTL7					X			1
CAB39					X			1
CAB39L					X			1
DDIT4					X			1

Genes	CALCIUM SIGNALING	FOCAL ADHESION	HUNTINGTONS DISEASE	INOSITOL PHOSPHATE METABOLISM	MTOR SIGNALING PATHWAY	PHOSPHATIDYLINOSITOL	VEGF SIGNALING	Total Pathways Shared
EIF4B					X			1
EIF4E					X			1
EIF4E1B					X			1
EIF4E2					X			1
EIF4EBP1					X			1
HIF1A					X			1
INS					X			1
MTOR					X			1
PRKAA1					X			1
PRKAA2					X			1
RHEB					X			1
RICTOR					X			1
RPS6					X			1
RPS6KA1					X			1
RPS6KA2					X			1
RPS6KB1					X			1
RPS6KB2					X			1

Genes	CALCIUM SIGNALING	FOCAL ADHESION	HUNTINGTONS DISEASE	INOSITOL PHOSPHATE METABOLISM	MTOR SIGNALING PATHWAY	PHOSPHATIDYLINOSITOL	VEGF SIGNALING	Total Pathways Shared
RPTOR					X			1
STK11					X			1
STRADA					X			1
TSC1					X			1
TSC2					X			1
ULK1					X			1
ULK2					X			1
ULK3					X			1
ALDH6A1				X				1
C17orf67						X		1
CCDC176				X				1
CDS1						X		1
CDS2						X		1
DGKA						X		1
DGKB						X		1
DGKD						X		1
DGKE						X		1

Genes	CALCIUM SIGNALING	FOCAL ADHESION	HUNTINGTONS DISEASE	INOSITOL PHOSPHATE METABOLISM	MTOR SIGNALING PATHWAY	PHOSPHATIDYLINOSITOL	VEGF SIGNALING	Total Pathways Shared
DGKG						X		1
DGKH						X		1
DGKI						X		1
DGKQ						X		1
DGKZ						X		1
ETV5						X		1
HSPB1							X	1
INPP5D						X		1
IPMK				X				1
JMJD7							X	1
KRAS							X	1
LYRM5							X	1
MAP2K2							X	1
MAPK11							X	1
MAPK12							X	1
MAPK13							X	1
MAPK14							X	1

Genes	CALCIUM SIGNALING	FOCAL ADHESION	HUNTINGTONS DISEASE	INOSITOL PHOSPHATE METABOLISM	MTOR SIGNALING PATHWAY	PHOSPHATIDYLINOSITOL	VEGF SIGNALING	Total Pathways Shared
MAPKAPK2							X	1
MAPKAPK3							X	1
MINPP1				X				1
MIOX				X				1
NFAT5							X	1
NFATC1							X	1
NFATC2							X	1
NFATC3							X	1
NFATC4							X	1
NRAS							X	1
NTRK1							X	1
PLA2G10							X	1
PLA2G12A							X	1
PLA2G1B							X	1
PLA2G2A							X	1
PLA2G2C							X	1
PLA2G2D							X	1

Genes	CALCIUM SIGNALING	FOCAL ADHESION	HUNTINGTONS DISEASE	INOSITOL PHOSPHATE METABOLISM	MTOR SIGNALING PATHWAY	PHOSPHATIDYLINOSITOL	VEGF SIGNALING	Total Pathways Shared
PLA2G2E							X	1
PLA2G2F							X	1
PLA2G3							X	1
PLA2G4A							X	1
PLA2G4B							X	1
PLA2G4E							X	1
PLA2G5							X	1
PLA2G6							X	1
PTGS2							X	1
SH2D2A							X	1
SLC26A1						X		1
TPI1				X				1
WIBG						X		1

Table S3. Gene-enrichment analysis in the seven significant pathways. The column “Total Pathways Shared” counts how many times that gene is shared among the pathways, whereas the row “total genes by pathway” counts the number of genes in each pathway.
